# Cross‐continental transmission and host adaptation of *Mycobacterium tuberculosis* in China unveiled by population history reconstruction and adaptive evolution signal detection

**DOI:** 10.1002/imo2.70052

**Published:** 2025-09-20

**Authors:** Wei Wu, Zhuochong Liu, Haiqi Chen, Yuhan Tang, Zhonghua Jiang, Yiyang Zhang, Andong Zhang, Zhiwei Zhou, Robert S. Marks, Fan Zhang, Haibing Yuan, Yan Yu, Kangshan Mao, András Dinnyés, Nalin Rastogi, Jianping Xie, Qun Sun

**Affiliations:** ^1^ Key Laboratory of Bio‐resources and Eco‐environment of the Ministry of Education, College of Life Sciences Sichuan University Chengdu China; ^2^ Institute of Modern Biopharmaceuticals, School of Life Sciences Southwest University Chongqing China; ^3^ Department for Biotechnology Engineering Ben Gurion University of the Negev Be'er Sheva Israel; ^4^ Center for Archaeological Science Sichuan University Chengdu China; ^5^ BioTalentum Ltd. Gödöllő Hungary; ^6^ Department of Physiology and Animal Health, Institute of Physiology and Animal Nutrition Hungarian University of Agriculture and Life Sciences Gödöllő Hungary; ^7^ WHO Supranational TB Reference Laboratory Institut Pasteur de Guadeloupe Guadeloupe France

**Keywords:** DNA information entropy, host adaptation, macrophage, *Mycobacterium tuberculosis*, neutral mutation simulation, transmission

## Abstract

The transmission history and adaptive evolution of *Mycobacterium tuberculosis* complex (MTBC) in China remain underexplored despite its remarkably low diversity and enduring public health burden. Here, we analyzed 23,873 whole genome sequences of MTBC to reconstruct its spread timeline and routes, population dynamics, and host adaptation patterns in China. The Bayesian coalescent models revealed the recurrent introductions of four MTBC sub‐lineages (L2.2, L4.2, L4.4, and L4.5) during 1000–400 years ago, by European‐Chinese transmission networks, which might have triggered the formation of their local genetic clades in China. These local clades underwent three rapid population expansions that temporally aligned with historical climate cooling events and warfare, and displayed convergent adaptation, including shared mutations and structural variations in macrophage‐resistance genes and enhanced genetic diversity in T cell epitopes. The historical mutation rate estimations and neutral mutation simulations indicated that these local clades experienced pronounced macrophage‐induced pressures, likely operative over the past two centuries. The DNA information entropy analysis further revealed their adaptive evolution signatures clustered within macrophage‐resistance pathways. The gene *Rv0801*, computationally predicted to exhibit the most prominent adaptive signatures despite its uncharacterized function, was confirmed through recombinant strain infection to enhance intracellular survival in human macrophages. This integrative approach reveals MTBC's evolutionary trajectory in China, provides novel methods for quantifying selection pressures and detecting adaptive evolution signals in prokaryotes, and constructs a comprehensive framework for investigating pathogen transmission dynamics and host adaptation mechanisms.

## Highlights

- Four sub‐lineages of *Mycobacterium tuberculosis* complex (MTBC) might enter China 1000–400 years ago via dispersal networks linking Europe and China, evolving into local clades, which subsequently underwent three population expansions, temporally coinciding with historical climate cooling and warfare periods.
- MTBC local clades in China exhibited convergent adaptations, characterized by their shared mutations and structural variations in macrophage resistance genes alongside diversified T cell epitopes, reflecting the potential macrophage‐induced selection pressures since the recent centuries, with experimental validation that *Rv0801* can enhance bacterial survival in macrophages.
- Two novel bioinformatics methods were established to estimate the selection pressures via neutral mutation simulation and to detect the adaptive evolution signals based on DNA information entropy.

## Graphical abstract

This study showed that four *Mycobacterium tuberculosis* complex (MTBC) sub‐lineages might be introduced into China 1000–400 years ago via Europe‐China dispersal routes, where they diversified into local clades, and later underwent population expansions that coincided with periods of climatic cooling and warfare. Two novel bioinformatics methods were developed to quantify selection pressures and detect adaptive evolution signals. With these tools, these local clades in China were found to undergo convergent adaptation in macrophage‐resistance genes and T cell epitopes, with experimental validation that *Rv0801* (the strongest adaptive signal gene) can enhance bacterial survival in macrophages. Collectively, this study delineated the cross‐continental transmission, historical population expansions, and host adaptation of MTBC in China.

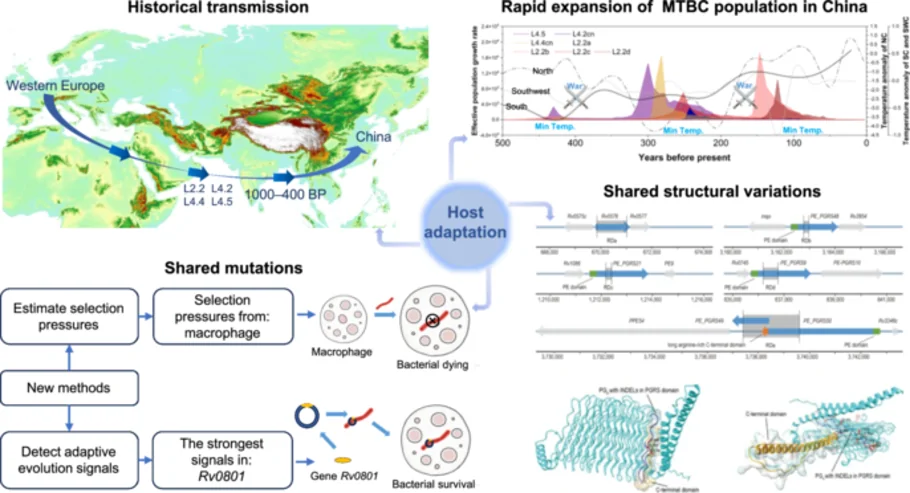

## 1 INTRODUCTION

As an ancient and widespread infectious disease, tuberculosis (TB), once called the “White Plague,” has left a profound imprint on human history. *Mycobacterium tuberculosis* complex (MTBC), the causative agent of TB, claimed around 1 billion lives in the past two centuries [1]. Unfortunately, it continues to afflict people worldwide and is estimated to latently infect 20%–26% of the present global population [2, 3]. In 2023, TB ranked as the most fatal infectious disease, with 10.8 million new cases and 1.25 million deaths globally [4]. China, with about 0.74 million new cases in 2023, had the third‐highest number of new cases in the world [4]. And many MTBC carriers do not manifest clinical symptoms. TB has been a global endemic for at least 3000–6000 years [5], spreading worldwide through human migration, commercial activities, and colonization [6, 7]. In China, the earliest arrival of MTBC might be dated back to at least 2000 years ago, as indicated by suspected skeletal TB lesions [8], and the positive molecular signal of suspected *Mycobacterium* DNA found in archaeological materials [9]. However, despite the long history of MTBC transmission and the large host population in China, the lineage diversity of MTBC in China is presently relatively low [7, 10], which contrasts with the intricate genetic makeup of MTBC in Europe, Southeast Asia, South Asia, and Africa [11, 12, 13, 14]. Even some adjacent countries, such as Vietnam [12], Russia [14], and India [13], currently exhibit a significantly higher lineage diversity of MTBC strains in clinical samples from China [10]. The observed low lineage diversity of MTBC in China suggests distinct transmission dynamics and potential host adaptation mechanisms in this population.

One hypothesis explaining this low lineage diversity may be the recent introduction of specific lineages, which have replaced older lineages of MTBC in China. MTBC is divided into human‐adapted lineages and those adapted to animal hosts [15, 16]. The former, causing human TB, includes nine lineages (L1‐9) [15, 17]. In China, the vast majority of clinically isolated MTBC strains belong to lineages L2 and L4, which are also the main lineages currently widespread globally [18]. These strains are almost exclusively limited to four distinct sub‐lineages (L2.2, L4.2, L4.4, and L4.5) in China [7], even though L2 and L4 are known to comprise at least 10 sub‐lineages. However, the specific time and routes of MTBC introduction to and spread within China remain subjects of ongoing debate [7, 12, 18, 19, 20]. Additionally, MTBC shows rapid population expansion in Chinese history [7, 18], where the past introduction of broadly disseminated MTBC lineages likely reshaped indigenous MTBC population structures [11]. Nevertheless, the contributions of environmental drivers or sociohistorical contingencies to this epidemiological process remain incompletely resolved. An alternative hypothesis posits that select sub‐lineages underwent host adaptation processes following their introduction into China, establishing equilibrium with the indigenous human host population, thereby constraining the proliferation of contemporaneous lineages. Despite globalization, MTBC lineages maintain distinct and stable geographic distributions [21], suggesting long‐term local adaptation to host populations [11]. Considered that human‐specific MTBC lineages exclusively infect humans [15], and vary in tropism, virulence, and immunogenicity, which may be influenced by host genetics [22], the prevalent lineages in China could have co‐evolved with local populations. The genetic characteristics of this adaptation may also be vertically transmitted within certain clades due to the absence of horizontal gene transfer in MTBC [23]. Previous studies suggested MTBC's adaptation to the host's lung environment [24], and the possible adaptation of MTBC to the high‐altitude environment of Tibet [25]. However, genomic evidence for the adaptation of MTBC to local human populations is lacking, as the mixing of host populations with diverse genetic backgrounds could obscure the sympatric relationship between pathogens and their hosts [11]. In China, the Han Chinese constitute more than 90% of the 1.4 billion population, providing both a historically persistent and comparatively homogeneous genetic background [26], and a substantial reservoir for MTBC transmission. This positions the Chinese population as an ideal case for studying the host adaptation of MTBC. Moreover, given the critical role of the human leukocyte antigen (HLA) system in epitope recognition and its allelic diversity across human populations [27], MTBC epitopes are potential markers for host adaptation. Previous studies have shown that lineages with widespread distributions possess a greater number of nonsynonymous mutations in epitopes [11], and the variations in T cell epitopes could affect the response of T cells to MTBC strains [28]. Similarly, macrophages, crucial for immune defense and antigen presentation, may exert selection pressures driving MTBC's adaptation to hosts due to MTBC strains' unique intracellular lifecycle within these cells [29]. Furthermore, MTBC has evolved multiple sophisticated mechanisms to counteract macrophage‐mediated antimicrobial attacks [30, 31]. Thus, the genes involved in virulence, cell envelope formation, and other functions may also be shaped by host adaptation. Therefore, this study also aims to determine whether the adaptation of MTBC to host populations is a significant factor contributing to its low lineage diversity in China, and, if so, to elucidate the temporal framework of this adaptation process and identify potential selective pressures driving its evolutionary trajectory, along with characterizing the principal source of these selective pressures.

To address these questions, we firstly analyzed the global distribution, population dynamics, and diversification patterns of the circulating MTBC sub‐lineages in China, by integrating Bayesian coalescent and phylodynamic analysis with historical mutation rate estimations to reconstruct the transmission pathways and evolutionary trajectories. Then we developed the novel genetic analytical methods, by incorporating neutral mutation simulations with the DNA information entropy analysis to systematically evaluate the selection pressures across immunological epitopes and functionally critical gene groups, and identifying the candidate genes that showed the adaptive evolution signals, among which, *Rv0801* that exhibited the prominent adaptive evolution signals identified was further examined through the recombinant strain infection experiment to assess its potential macrophage survival advantages. We further conducted the comprehensive profiling of the structural variations prevalent in the local MTBC clades in China. This study aims to (1) elucidate the underlying determinants that may contribute to MTBC's low lineage diversity in China, (2) identify critical genomic signatures of host adaptation and sources of selective pressures acting on MTBC populations, (3) reveal the potential correlations between historical anthropogenic activities and climatic variables with MTBC population dynamic, and (4) develop novel methodological methods for detecting adaptive evolution signals in prokaryotic populations.

## 2 RESULTS

### Population structure of MTBC in China

**Figure 1:**
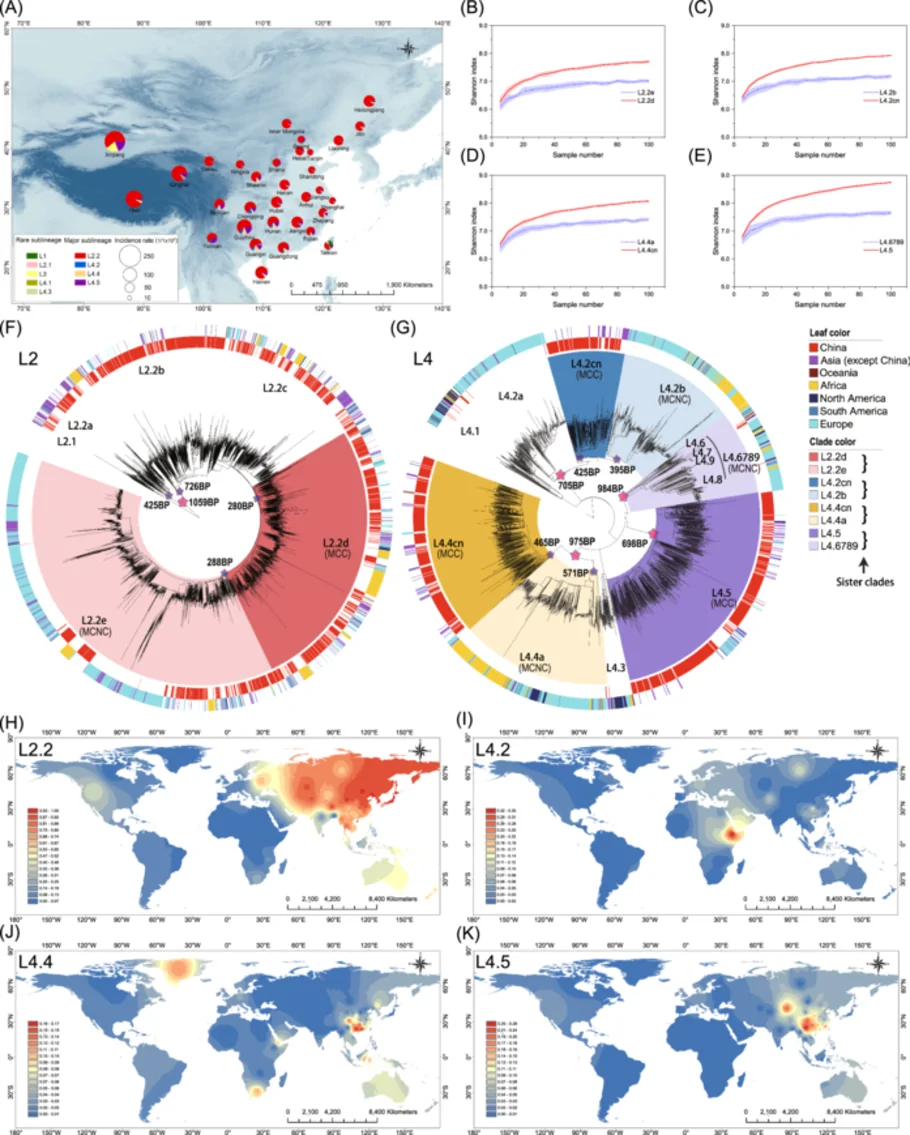
Prevalence of the sub‐lineages currently predominant in China. (A) Prevalence and incidence rate of MTBC sub‐lineages in 32 provinces of China (excluding Hong Kong and Macau). (B−E) Curve of Shannon index (represents genetic diversity) with increasing randomly sampled strains number of four pairs of the MTBC clades predominantly found in China (MCCs) and MCNCs (the MTBC clades predominantly found in non‐China regions) for the whole genome genes. (F−G) Phylogenetic trees of L2 (F) and L4 (G), reconstructed with 4321 and 2626 strains by the maximum likelihood method. The outgroup is L1, and for better displaying the phylogenetic trees' topology in illustrations, the branch length for the outgroup is artificially shortened. And “BP” is short for “years before present,” as the “present” is A.D. 2020. (H−K) Global distributions of the MTBC sub‐lineage L2.2 (H), L4.2 (I), L4.4 (J), and L4.5 (K).

Genotyping results of nationwide MTBC isolates in China revealed a population structure predominantly composed of lineages L2 and L4, with distinct geographical distribution patterns and a homogeneous sub‐lineage composition. For consistency in description, given that they generally aligned with lineage classifications based on the whole‐genome sequencing (WGS) data, the spoligotyping results were assigned to the relevant MTBC lineages according to previously established associations between spoligotyping patterns and phylogenetic lineages [16]. L2 and L4 are the primary MTBC lineages currently prevalent in China (Figure 1A). Based on the genotyping results collected from China, L2 accounts for 78.2% (4530/5793) and L4 for 20.6% (1194/5793) of clinical MTBC isolates, consistent with previous studies [7, 10, 19]. L2 constitutes the largest proportion of MTBC infections across all provinces, while the proportion of L4 decreases from South to North. Additionally, the prevalence of MTBC is relatively higher in West and South China. Despite this, the sub‐lineages of L2 and L4 predominantly distributed in China are relatively homogeneous in composition. L2 comprises sub‐lineages L2.1 (also known as the “proto‐Beijing family”) and L2.2. Here, L2.1 accounts for only 2.7% of all L2 (121/4530), and is only detected in South China, while L2.2 comprises the vast majority (97.3%, 4409/4530) and is widely distributed across various regions. The L4 lineage is represented by only three sub‐lineages in China, with L4.2 accounting for 16.1% (192/1194), L4.4 for 35.5% (424/1194), and L4.5 for 47.9% (572/1194) of clinical MTBC isolates.

### Majority of MTBC strains from China cluster in specific clades

Phylogenetic analysis identified the distinct clades in China, which exhibited higher intra‐clade genetic diversity than their sister clades (Figure 1B–G). From the WGS data of 23,873 MTBC strains, 17,568 strains were classified as L2 or L4, with 6962 strains having associated geographic labels. The phylogenetic trees were constructed using whole‐genome single nucleotide polymorphisms (SNPs) from the L2 and L4 strains with geographic labels, while the remaining strains, including L1‐9, were employed for ancestral sequence reconstruction to establish reference sequences for SNP identification. The phylogenetic trees of L2 and L4 show that the vast majority of MTBC strains from China cluster in specific clades or sub‐lineages. Most L4 strains from China cluster in the sub‐lineage L4.5 and two independent clades: L4.2cn (belonging to the sub‐lineage L4.2) and L4.4cn (belonging to the sub‐lineage L4.4). The L2 strains from China are mainly clustered across the clades L2.2a‐d (belonging to the sub‐lineage L2.2), while those from other countries (primarily European countries) are mostly clustered in the independent clade L2.2e. For clarity in subsequent studies, four pairs of sister clades (or sub‐lineages) within the L2 and L4 phylogenetic trees were identified. Each pair consists of two clades (or sub‐lineages) (Figure 1F,G): L2.2 d/L2.2e, L4.2cn/L4.2b, L4.4cn/L4.4a, and L4.5/L4.6789, designated as the MTBC clade predominantly found in China (MCC) and the MTBC clade predominantly found in non‐China regions (MCNC), respectively. The former predominantly comprises strains from China and represents the local clade, while the latter is characterized by a majority of strains from countries outside China. Interestingly, the intra‐clade genetic diversity of MCCs is all higher than that of their corresponding MCNCs (Figures 1B–E and S1).

### Global distribution and diversification timeline

Bayesian dating with a relaxed molecular clock model identified the multiple MTBC diversification events in China between 1000 and 200 years ago (Figures 1F,G and S2). The worldwide geographic distributions of the L2.2, L4.2, L4.4, and L4.5 sub‐lineages were estimated through the interpolation analysis. The results indicate that, except for L4.5, the other three sub‐lineages show global distribution characteristics (Figure 1H–K). Based on the estimates of the origin time of MTBC and its lineages using ancient DNA collected by Sabin et al. [5], we employed Bayesian methods to estimate the diversification time of the four sub‐lineages using WGS data. The distribution of sampling locations for the WGS data with geographic tags is presented in Figure S3. Our results showed that these four sub‐lineages diversified about 1100–700 years ago (Figure 1F,G), aligning with previous studies [7, 25]. Focusing on the clades or sub‐lineages predominantly composed of strains from China, L2.2b and L4.5 appeared around 700 years ago, while L2.2a, L4.4cn, and L4.2cn appeared 500–400 years ago, and L2.2d around 300 years ago.

### Origin and global spread

**Figure 2:**
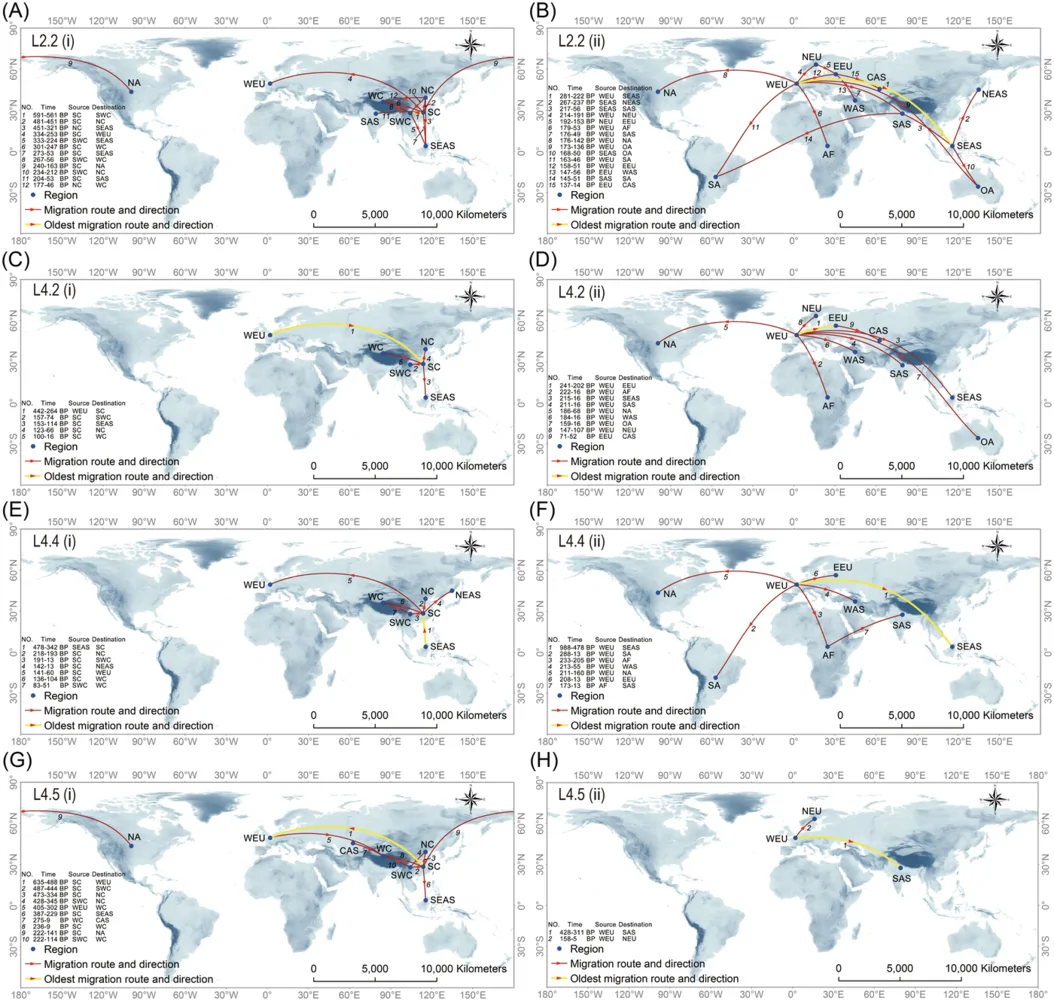
Historical spread of the four sub‐lineages currently predominant in China. (A−H) Historical migration routes with time and directions for L2.2 (A, B), L4.2 (C, D), L4.4 (E, F), L4.5 (G, H) by transmission dynamics methods. The yellow migration route in each figure represents the oldest of all the routes on this figure. And the color of the map represents the elevation. Due to numerous migration routes, each sub‐lineage's migration routes were divided into two parts, based on the two centers, South China (A, C, E, and G) and West Europe (B, D, F, and H), for separate illustration. For the abbreviations on the map, NC stands for North China, SC for South China, WC for West China, SWC for Southwest China, NA for North America, SA for South America, AF for Africa, WEU for West Europe, EEU for East Europe, NEU for North Europe, OA for Oceania, WAS for West Asia, SAS for South Asia, CAS for Central Asia, SEAS for Southeast Asia, NEAS for Northeast Asia. And “BP” is short for “years before present,” as the “present” is A.D. 2020.

Transmission dynamics and geographic tracing revealed the early emergence of South China‐Western Europe transmission events during 600–300 years ago among four MTBC sub‐lineages, with both regions identified as the primary global dispersal hubs over the past 200 years. Transmission dynamics methods estimated the timing and direction of migration routes for the four sub‐lineages (Figure 2A–H). The geographic divisions of China used here are shown in Figure S4. For the sub‐lineage L2.2, it may first appear in South China, spreading to Western Europe around 600 years ago, and gradually diffusing to other areas of China and Asia. Around 200 years ago, Western Europe became an active dispersion center, with L2.2 spreading globally from this region. The sub‐lineage L4.2, likely originated in Western Europe and entered South China around 400 years ago. Its early spread was not active until about 200 years ago when it began to spread extensively from Western Europe to all continents, and from South China to other Chinese regions and Southeast Asia. The sub‐lineage L4.4 also likely originated in Western Europe and did not enter China directly; instead, it first spread to Southeast Asia about 1000–450 years ago, then spread from Southeast Asia to South China around 500–350 years ago. About 200 years ago, it spread from Western Europe to all continents, and from South China to surrounding areas. Finally, the sub‐lineage L4.5 likely originated in South China, primarily spreading within China, with only a few strains entering Western Europe, Southeast Asia, and North America around 600, 300, and 200 years ago, respectively. Moreover, the FEAST algorithm was used to trace the geographic origins of the four sub‐lineages (Figure S5). The results indicated that for sub‐lineage L2.2, there were two major potential source regions, Fujian (0.44 ± 0.24; values following ± represent standard deviation, hereafter the same) and Anhui (0.33 ± 0.24), both provinces in South China. For L4.2, the United Kingdom was identified as the primary potential source region (0.72 ± 0.11), while L4.4 mainly spread from the Netherlands (0.63 ± 0.14). The Zhejiang province in South China was the principal potential source region for L4.5 (0.66 ± 0.11).

### Low temperatures and war facilitated population expansions of local clades

**Figure 3:**
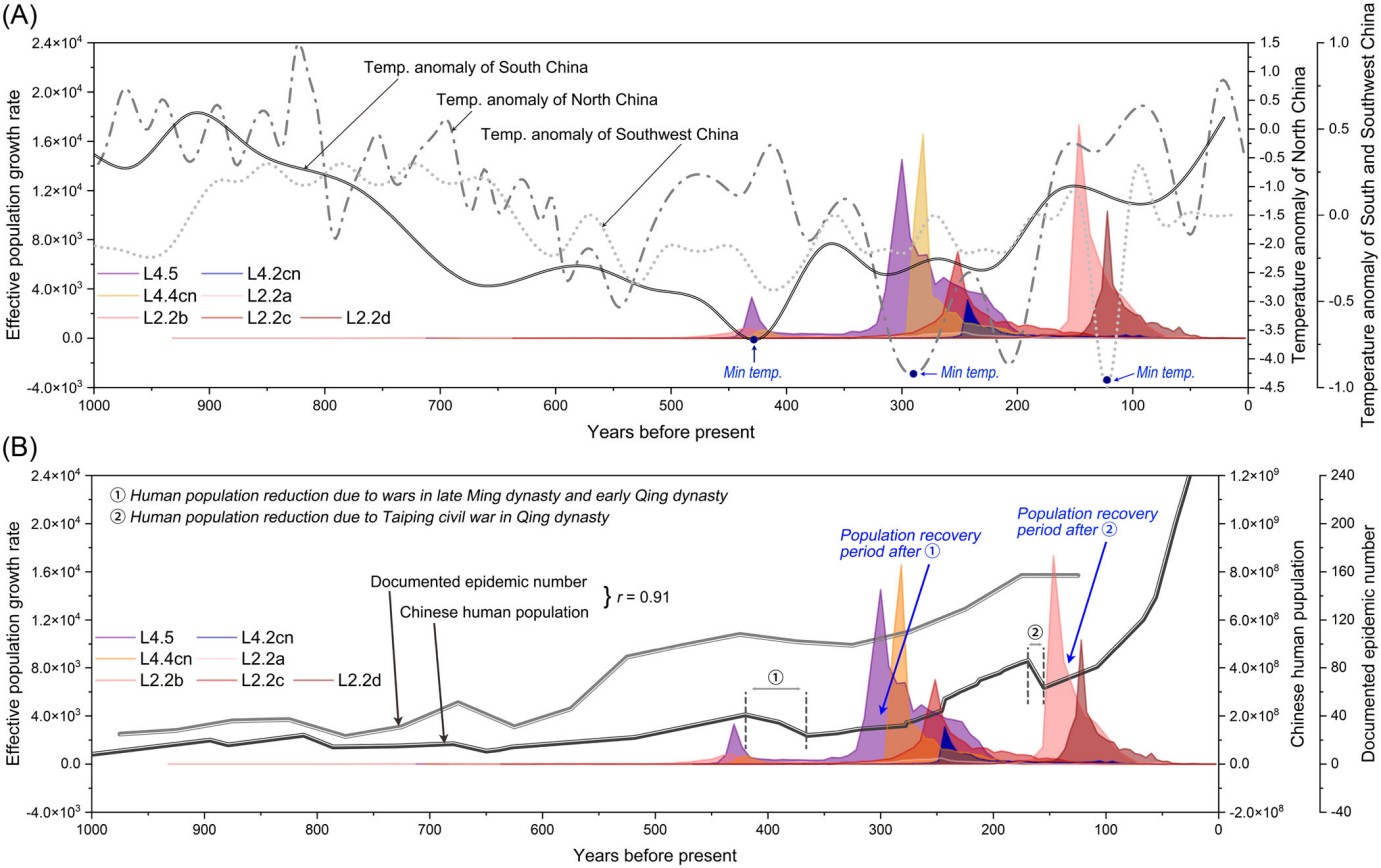
Historical population expansions of MTBC's local clades in China. (A) Changes of effective population growth rate of local MTBC clades in China and reconstructed historical temperature of South, North, and Southeast China in the last 1000 years. (B) Changes of effective population growth rate of local MTBC clades in China, historical Chinese human population, and the number of all types of epidemic outbreaks documented in the ancient Chinese literature. *r* represents the Pearson correlation coefficient. The time scale is expressed as “years before present,” with the most recent time as A.D. 2020. And “temp.” is short for “temperature.”

The Bayesian Skyline Plot (BSP) analysis identified three rapid expansions of MTBC local clades in China, temporally linked to war and climatic cooling over the past millennium. We examined the variation of the effective population size and the growth rate for all local clades/sub‐lineages (L2.2a‐d, L4.2cn, L4.4cn, and L4.5) distributed within China, over time. Three concentrated periods of rapid growth in the effective population size were identified around 450–400, 350–200, and 150–100 years ago, respectively, in South, North, and Southwest China (Figure 3A,B), which coincided with the lowest annual temperatures of these regions over the past 1000 years (Figure 3A). In addition, we examined the potential impact of Chinese population size changes on the expansion of MTBC. We gathered historical Chinese population data and compiled counts of epidemic outbreaks over the past 1000 years from ancient Chinese literature, such as historical records and local chronicles. Since the records don't distinguish between specific epidemic types, they encompass various widespread infectious diseases. We found a positive correlation between the historical changes in the Chinese population and the number of epidemic outbreaks documented (after normalization, *r* = 0.91, *F* = 0.37, *DTW* = 0.49, where *r* represents the Pearson correlation coefficient, *F* represents the Fréchet distance, and *DTW* represents the Dynamic time warping distance). This suggests a positive correlation between the outbreaks of infectious diseases, including TB, and the size of the host population, consistent with the views of Eisen et al. [32]. Moreover, when comparing the human population size changes to the effective population size changes of MTBC in China, results showed that the last two significant periods of rapid MTBC population growth coincided with periods of the Chinese population recovery following large‐scale declines due to war (Figure 3B). This correlation highlights the dynamic interaction between human demographics and MTBC expansion, suggesting that the recovery of host population may facilitate the expansion of MTBC. In summary, these findings suggested that climatic cooling periods and post‐war population recovery periods in Chinese history might have facilitated the three major population expansions of MTBC in China. However, it should be noted that this association we observed merely represents a temporal correlation, and the potential causal relationship between them remains to be established by future studies.

### Historical mutation rate estimation

**Figure 4:**
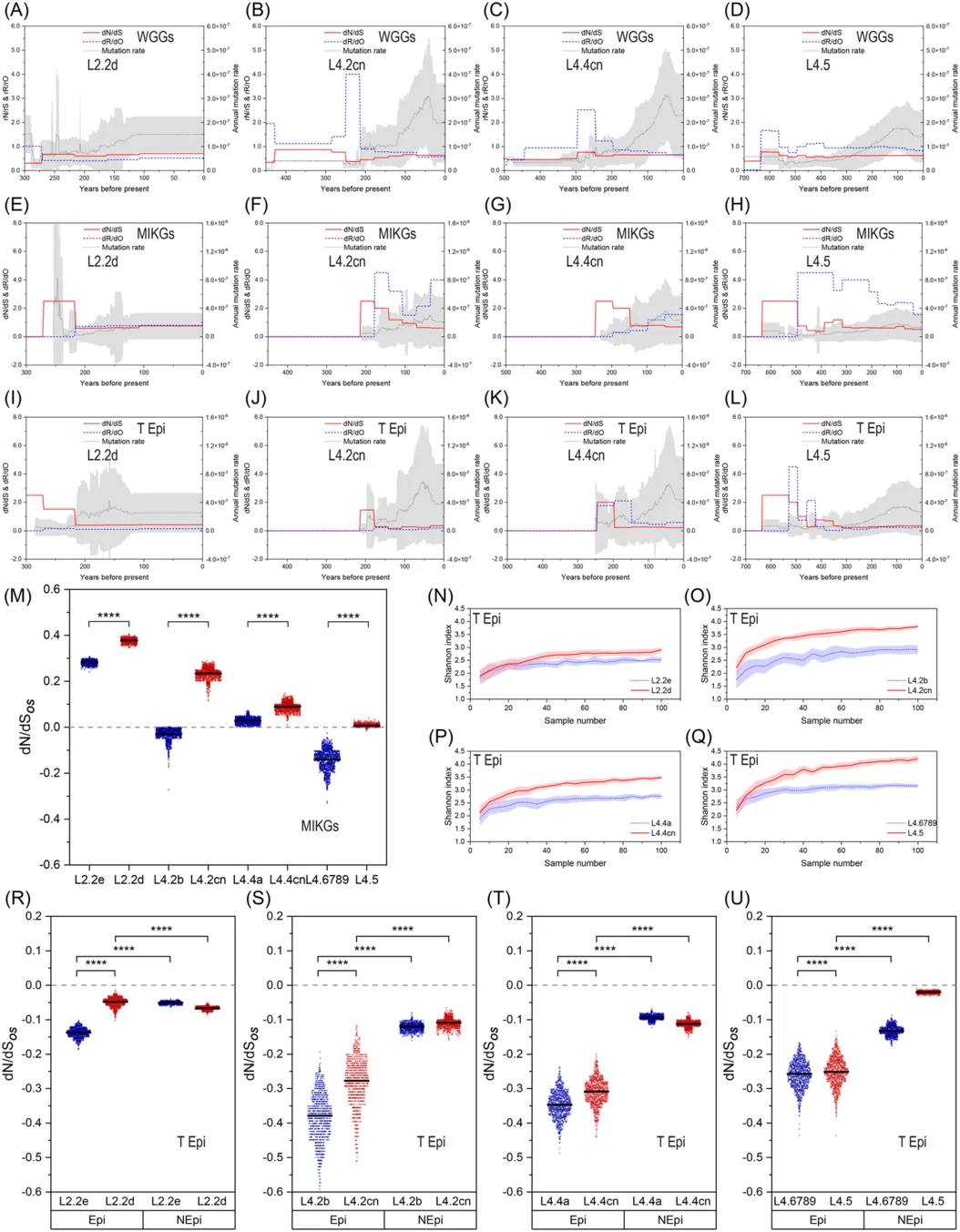
Selection pressures on the macrophage infection key genes (MIKGs) and T cell epitopes (T Epi) of the MTBC clades predominantly found in China (MCCs). (A−L) Mutation rate, dN/dS, and dR/dO changes of the whole genome genes (WGGs, A−D), MIKGs (E−H), and T Epi (I−L) for the four MCCs. (M) MIKGs' dN/dS_*OS*_ of the four MCCs and corresponding MCNCs (the MTBC clades predominantly found in non‐China regions). (N−Q) Curve of Shannon index (represents genetic diversity) with increasing randomly sampled strains number of the four pairs of MCCs and MCNCs for T Epi. (R−U) T Epi's dN/dS_*OS*_ of the four MCCs and corresponding MCNCs. NEpi (non‐epitope) represents the sequence other than Epi within the coding sequence that include Epi. The *p* values are given by the two‐sided Mann–Whitney *U* test, and **** presents *p* < 0.0001. The time scale is expressed as “years before present,” with the most recent time as A.D. 2020.

The macrophage infection key genes (MIKGs) and T cell epitopes of MTBC local clades in China have exhibited the elevated mutation rates over the past 300–200 years. We estimated the historical mutation rates and selection pressures for the four sub‐lineages (Figure S6A–D), MCCs (Figure 4A–D), and MCNCs (Figure S6E–H) across the entire genome over time. The mutation rates increased rapidly from 300 to 200 years ago in all sub‐lineages and MCCs. Throughout this period of accelerated mutation, dN/dS values remained stable and consistently below 1, indicating the genome‐wide evolution dominated by the purifying selection. We further characterized the selection on synonymous mutations by using dR/dO, which was defined as the ratio of optimal‐to‐rare versus rare‐to‐optimal codon transitions. Across all genes, dR/dO exhibited a significantly greater variation than dN/dS (Figure S6A–D), particularly for L4.2 (dR/dO = 0.69 ± 0.52; values following ± represent standard deviation, hereafter the same). And the dR/dO of L2.2 generally maintained relatively low values (0.40 ± 0.17), whereas the dR/dO of L4.5 was consistently relatively high (0.93 ± 0.37). MIKGs are the genes related to MTBC strains' resistance to macrophage attacks, invasion into macrophages, and survival within them. Among the four MCCs, L4.5 showed strong positive selection pressures on MIKGs (dN/dS > 2) as early as 600–500 years ago, while the remaining three MCCs began to show a strong positive selection and a higher mutation rate 250–200 years ago (Figure 4E–H). The same trends were also observed in T cell epitopes (Figure 4I–L).

### Selection pressures on local clades from host macrophages

Neutral mutation simulations revealed the intensified positive selection pressures on the macrophage‐resistance genes of MTBC local clades in China compared to their phylogenetically related sister clades from other geographic regions. We quantified the deviations in actual dN/dS and dR/dO values from those anticipated under 1000 neutral mutation simulations (dN/dS_*OS*_ and dR/dO_*OS*_). This analysis was used to compare selection pressures across various key gene groups among different clades. For MIKGs, the dN/dS_*OS*_ values of all four MCCs were always > 0 and significantly higher (*p* < 0.0001) than that of their corresponding MCNCs (Figure 4M). Conversely, the dR/dO_*OS*_ values of all four MCCs were significantly lower (*p* < 0.0001) than those of their corresponding MCNCs. (Figure S7A). These results indicate that MCCs, after differentiation, were under positive selection on MIKGs, with significantly higher pressures than on the corresponding MCNCs, and exhibited a lower tendency to select rare codons in synonymous mutations. Additionally, the T cell epitopes of all the four MCCs showed a higher genetic diversity than that of the MCNCs (Figures 4N–Q and S1I–P), suggesting that they may have undergone consistent selective pressures. We therefore further calculated the dN/dS*os* values of the T cell epitopes. For the whole genome T cell epitopes, the dN/dS_*OS*_ < 0 ruled for all clades (Figure 4R–U). And the dN/dS_*OS*_ remained significantly lower (*p* < 0.0001) than that for the non‐epitope regions of the whole genome coding sequences (CDSs) containing T cell epitopes, except for L2.2d. This indicated an overall state of purifying selection. However, the dN/dS_*OS*_ values of the four MCCs demonstrated higher values compared to the MCNCs (*p* < 0.0001). This tendency was not observed for the non‐epitope regions, suggesting that it is not related to phylogeny. Therefore, with regard to T cell epitopes, the MCCs were subjected to stronger selection pressures compared to the MCNCs. This manifestation of selective pressures—specifically through genetic alterations in macrophage‐resistance genes and modifications to T‐cell epitopes that confer immune evasion capabilities—likely represents an adaptive evolution signature of MTBC within local clades in China. For the essential genes (EGs), as a control, all clades always had a dN/dS_*OS*_ < 0 (Figure S7B), highlighting the presence of purifying selection, which is consistent with the conservative nature of EGs [33]. For the gene groups related to virulence factors, stress conditions, dormancy and resuscitation regulation, energy metabolism, central carbon metabolism, nitrogen metabolism, amino acid biosynthesis, cofactors and vitamins biosynthesis and metabolism, and cell envelope formation (Figure S7C–P), and the B cell epitopes (Figure S7Q–T), the results showed that overall negative selective pressure active in the MCCs on these gene groups, and do not show consistent characteristics.

### Genes in adaptive evolution identified through information entropy

**Figure 5:**
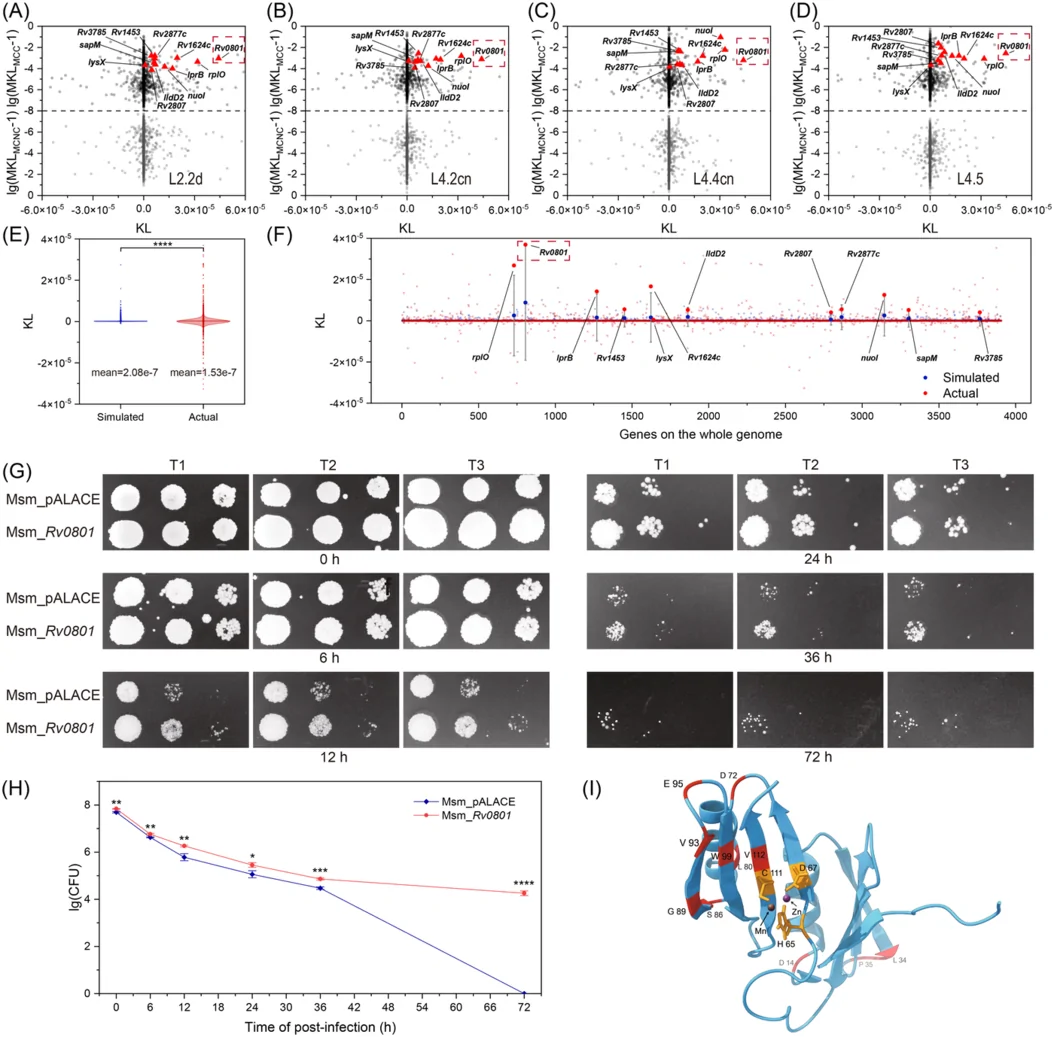
Candidate genes undergoing adaptive evolution of the MTBC clades predominantly found in China (MCCs). (A−D) Genes with the highest KL and MKL shared across four MCCs. Gray dots represent all genes in whole genome, and red triangles represent genes with the highest KL and MKL shared by four MCCs. (E, F) Mean values of KL obtained through random mutations simulations and actual KL of whole genome genes for the sample set including all strains of the four MCCs. KL represents the degree of relative entropy caused by mutations in genes, and the sign of KL indicates whether there is an increase (+) or decrease (−) in information entropy. MKL_MCC_ represents the ratio of KL of the MCC to that of its corresponding MCNC (the MTBC clades predominantly found in non‐China regions), whereas MKL_MCNC_ represents the opposite ratio. The *p* values in (E) are given by the two‐sided Mann–Whitney *U* test, and **** presents *p* < 0.0001; the horizontal lines in (E) denote the mean values of the corresponding data points on the graph. (G) Intracellular survival of Msm in cell lysate at 0–72 h postinfection. Control strains with empty vectors (Msm_pALACE) or recombinant strains (Msm_*Rv0801*) were used to infect 1 × 10^6^ THP‐1 cells/mL at an MOI of 10: 1. For each group, from left to right, colonies of Msm were obtained by cell culture after 10‐fold serial dilutions (1 × 10^1^, 1 × 10^2^, and 1 × 10^3^), and T1‐3 represents the three parallel replicates. (H) The level of infection was assessed by counting bacterial colony numbers at 0–72 h postinfection (*n* = 3) for the results in (G). In the vertical coordinate, CFU stands for colony forming unit, representing the number of intracellular bacteria obtained after cell lysis. The *p* values in (H) are given by the independent samples two‐sided *t*‐test, * presents *p* < 0.05, ** presents *p* < 0.01, *** presents *p* < 0.001, and **** presents *p* < 0.0001. (I) The predicted encoding protein structure of *Rv0801*. Amino acids marked in yellow indicate potential Mn or Zn ion binding sites, while amino acids marked in red represent that they are found to have mutations in MCCs.

The information entropy analysis identified the genes with pronounced adaptive evolution signals shared across MTBC local clades in China. We calculated the degree of relative entropy (KL) caused by mutations in the target CDS of the MCCs, the ratio of relative entropy in the MCCs to that in corresponding MCNCs (MKL_MCC_), and the ratio of relative entropy in MCNCs compared to MCCs (MKL_MCNC_). The KL and MKL values for all genes in the whole genome for the four MCCs are presented in Figure 5A–D. Here, the points in the upper right corner represent genes in each MCC where KL > 0 and is greater than that of the corresponding MCNC (MKL_MCC_ > 0). A total of 12 genes (marked as red triangles in Figure 5A–D) have the highest values of both KL and MKL_MCC_ shared by all four MCCs, with the most significant gene being *Rv0801*. For each of these 12 genes, at least one of the dN/dS_*OS*_ or dR/dO_*OS*_ values is > 0.7, or even approaches 1, across all MCCs (Table S1). Furthermore, we performed 100 random mutation simulations, computing each gene's KL value under random mutation across all the genes of the whole genome. It is observed that the mean simulated KL of all random processes for most genes is > 0 (Figure 5E,F), consistent with the understanding that random mutations lead to an increase in information entropy [34]. Moreover, the simulated KL values are significantly higher than the actual KL values overall (*p* < 0.0001); however, more extreme values are observed in the actual KL values (Figure 5E), and for each of the 12 genes mentioned above, the actual KL is higher than the mean simulated KL (Figure 5F).

### *Rv0801* Confers an intra‐macrophage survival advantage

The recombinant strain infection experiments demonstrated that the gene *Rv0801*, harboring the most pronounced adaptive evolution signals shared by all the MTBC local clades in China, significantly enhanced the intracellular survival duration of recombinant strains within human macrophages. To explore its function, *Rv0801* was heterologously expressed in *Mycobacterium smegmatis* (Msm), which is a phylogenetically proximal species to the MTBC and is a widely adopted nonpathogenic and fast‐growing surrogate used as a substitute for MTBC in molecular experiments. The successful construction (Msm_*Rv0801*) was confirmed by PCR (500 bp band) after the recombinant plasmid was extracted and introduced into Msm (Figure S8). Msm_*Rv0801* and the empty vector strain (Msm_pALACE) were used to infect the THP‐1 cells, which had been differentiated into macrophages. Intracellular bacteria were quantified by lysing cells at various times postinfection, diluting them serially (10‐fold), and plating them (Figure 5G). Then, a survival curve was generated based on colony counts (Figure 5H). The results showed that the intracellular survival rate of Msm_*Rv0801* was significantly higher than Msm_pALACE within 72 h postinfection. By 72 h, Msm_pALACE was eliminated, while Msm_*Rv0801* persisted, indicating that *Rv0801* confers recombinant Msm survival advantage within macrophages.

### Structure and functional domain of the *Rv0801*‐encoded protein

Structural prediction suggested the *Rv0801*‐encoded protein was a putative glyoxalase I [35]. The structure of the *Rv0801*‐encoded protein was predicted using AlphaFold2 (Figure 5I). The 4–115th amino acids of this protein form a Vicinal Oxygen Chelate (VOC) domain, and the 7–112th amino acids form a glyoxalase‐like domain, which were predicted by InterProScan. In addition, the 65th amino acid (H) and the 67th amino acid (D) are the zinc ion binding sites, while the 65th amino acid (H) and the 111th amino acid (C) are the manganese ion binding sites (Figure 5I), with probabilities of 0.87 and 0.85, respectively, which were predicted by MetalNet.

### Structural variations of MTBC local clades in China

**Figure 6:**
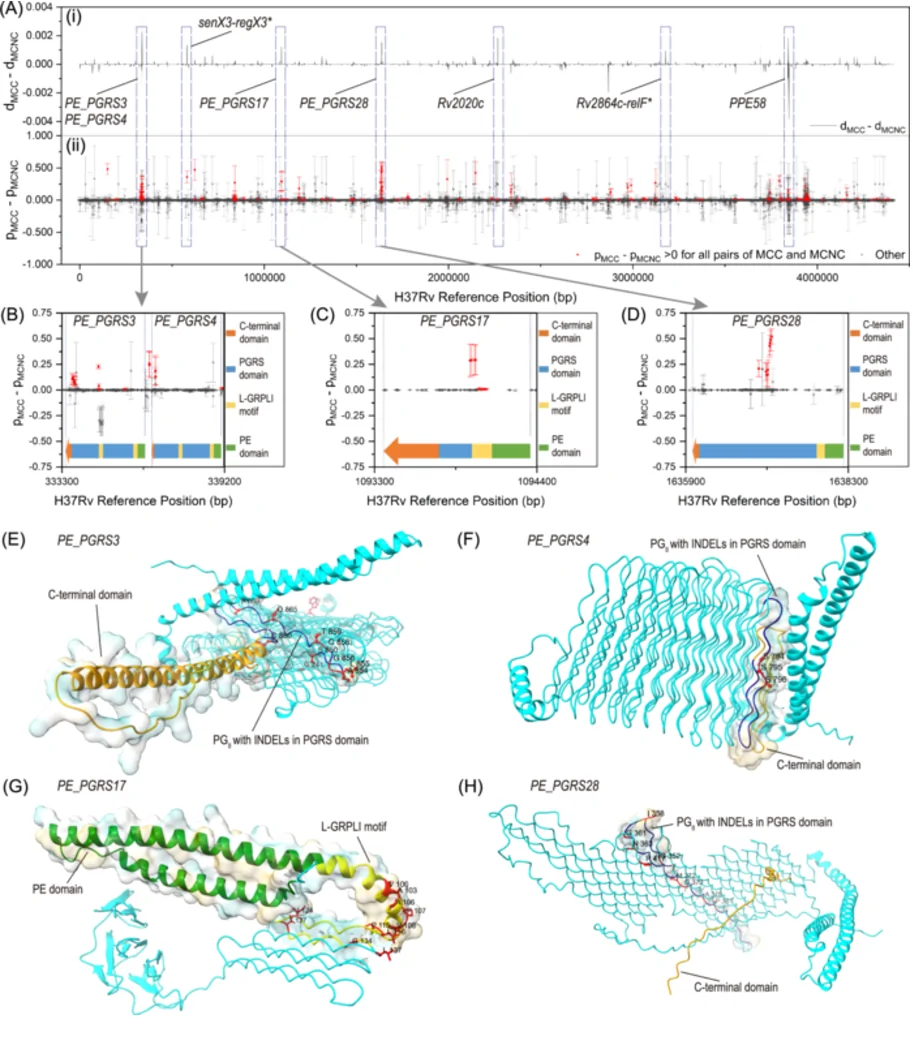
The small segment insertions and deletions (INDELs) of the MTBC clades predominantly found in China (MCCs). (A) The difference in INDELs density between MCCs and MCNCs (the MTBC clades predominantly found in non‐China regions) across the whole genome (*d*_MCC_ − *d*_MCNC_, (i) and the difference between MCCs and MCNCs in the proportion of strains having INDELs at each locus across the whole genome (*p*_MCC_ −* p*_MCNC_, (ii). Blue dashed boxes indicate the region of the coding sequence (CDS) or noncoding sequence (NCS) with high INDEL density. The asterisk (*) marks NCS regions located between the two genes indicated. Red points in (ii) represent genomic loci where *p*_*MCC*_ −* p*_MCNC_ > 0 for all four pairs of MCCs and MCNCs, referred to as “red sites.” (B−D) Magnified views of the regions containing genes *PE_PGRS3* and *PE_PGRS4* (B), *PE_PGRS17* (C), and *PE_PGRS28* (D) from (ii), which exhibit high *d*_MCC_ −* d*_MCNC_ and clusters of “red sites”. Arrows indicate the range of each gene's CDS, with the arrow direction showing the direction of translation. Different colored blocks represent different functional domains. (E−H) The encoding protein structures corresponding to the four genes shown in (B−D). Red highlights indicate amino acid residues with “red sites,” while dark blue highlights indicate poly‐glycine II helices where “red sites” are concentrated. Other colors indicate relevant functional domains.

Besides mutations, the structural variations may also play an important role in the adaptive evolution. Structural variation analysis revealed the shared structural variation signatures in multiple *PE_PGRS* genes of the MTBC local clades in China. Firstly, we analyzed the small segment insertions and deletions (INDELs) enriched in the MCCs (Figure 6A–H). The density of INDELs in each CDS and noncoding sequence (NCS) across the whole genome was calculated for each MCC. To eliminate INDELs due to phylogeny, we used the corresponding MCNC as a baseline and subtracted the density of INDELs of the corresponding MCNC (*d*_MCC_ −* d*_MCNC_). As a result, we identified six CDSs (*PE_PGRS3*, *PE_PGRS4*, *PE_PGRS17*, *PE_PGRS28*, *Rv2020c*, and *PPE58*), and two NCSs (*senX3*‐regX3* and *Rv2864c*‐relF*) with high *d*_MCC_ − *d*_MCNC_ values (Figure 6A). The authenticity of these INDELs was validated by identifying uncovered regions in the raw sequencing reads. For each genomic locus, the frequency of INDEL occurrence (designated as *p*_MCC_ and *p*_MCNC_ for MCCs and MCNCs, respectively) was computed across clades. For each pair of MCC and MCNC, the *p*_MCC_ − *p*_MCNC_ value was calculated. Among the eight CDSs and NCSs with high *d*_MCC_ − *d*_MCNC_ values, *PE_PGRS3*, *PE_PGRS4*, *PE_PGRS17*, and *PE_PGRS28* had clustered genomic loci with INDELs where *p*_MCC_ −* p*_MCNC_ > 0 in all the four MCC and MCNC pairs (Figure 6B–D). The other four CDSs or NCSs did not exhibit this particular characteristic (Figure S9A–D). We also performed 1000 random samplings of 100 strains each to validate that the concentrated occurrence or increase in INDELs in these four genes was a shared feature of MCCs using all the following strain genome sets: (1) all 23,873 MTBC, (2) lineages L1‐6, (3) animal‐adapted lineages (*Mycobacterium bovis*, *M. orygis*, *M. caprae*, *M. microti*, *M. pinnipedii*, *M. tuberculosis dassie bacillus*, *M. suricattae*, *M. mungi*), (4) four MCNCs, (5) four MCCs. For each sampling, we calculated the frequency of INDEL occurrence at each genomic locus within the four genes. We did not sample from lineages L7, L8, and L9 due to the insufficient number of strains available. Our results suggested that the clustered genomic loci with INDELs, which were shared among the MCCs (Figure 6B–D), had fewer occurrences of INDELs among strains found in other clades or lineages (Figures S10–12). This suggested that the propensity of strains within MCCs to experience INDELs at these loci was a unique and shared characteristic. In addition, the functional domains, where these concentrated genomic loci with INDELs were located, were identified (Figure 6B–D) and we marked the amino acid residues with these genomic loci on the protein structures of the four genes (red amino acid residues in Figure 6E–H). In *PE_PGRS3* and *PE_PGRS4*, most loci were concentrated on the last poly‐glycine II (PG_II_) helix within the PGRS domain, connecting the C‐terminal domain. In *PE_PGRS28*, the loci were concentrated on a single PG_II_ helix, which was located in the middle of the PGRS domain. And in *PE_PGRS17*, almost all the loci were concentrated on the l‐GRPLI motif connecting the PE domain and the PGRS domain. We also calculated dN/dS_*OS*_ values for these four genes and found that except for L2.2e, L2.2d, and L4.6789, which showed dN/dS_*OS*_ > 0 on *PE_PGRS28*, thus indicating positive selection, all the MCCs and MCNCs exhibited a purifying selection (dN/dS_*OS*_ < 0) on the other three genes (Figure S9E–H).

**Figure 7:**
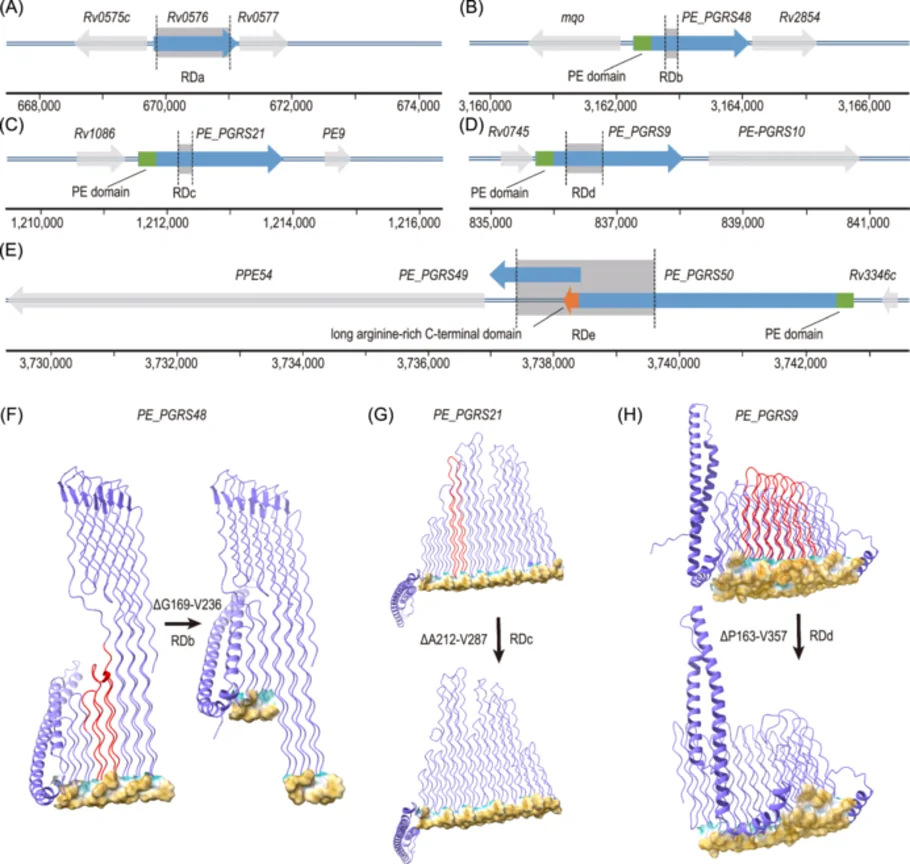
Clade‐specific regions of difference (RDs) of the MTBC clades predominantly found in China (MCCs). (A–E) Position of the five Clade‐specific RDs (RDa‐e) in the reference genome H37Rv. (F−H) Predicted protein structures before and after the deletion of RDb (F), RDc (G), and RDd (H). In the protein structures before the deletion of RDs, the red region represents the region to be lost due to RDs, and yellow regions in all protein structures represent the regions rich in hydrophobic amino acid residues.

In addition, we identified a total of five large segment deletions, also known as regions of difference (RDs), distributed across the L2.2 d, L4.4cn, and L4.5 clades and each occurring uniquely within a separate MCC (Figure 7A–E). The RDa in L4.5 and the RDb in L4.4cn were detected in 92.34% and 84.60% of all strains within these clades, possibly representing fixed evolutionary characteristics. The other three deletions, RDc, RDd, and RDe, were detected in 16.67%, 15.32%, and 10.10% of the strains in the L4.5, L2.2d, and L4.4cn clades, respectively. In L4.5, RDa led to the complete deletion of *Rv0576*. The 1–81th amino acids of the encoding protein of *Rv0576* form an HTH‐type metal‐responsive transcriptional regulator, and the 254–424th amino acids form a mycothiol‐dependent enzyme, as predicted by InterProScan. Additionally, the other four RDs (RDb‐e) occurred within the genes of the PE_PGRS family. Moreover, structural predictions showed that RDb‐RDd resulted in the loss of varying numbers of PG_II_ helices, significantly affecting the regions rich in hydrophobic amino acid residues (yellow regions in Figure 7F–H) of *PE_PGRS9* in L2.2d and *PE_PGRS48* in L4.4cn. As for *PE_PGRS21*, it experienced the loss of an odd number of PG_II_ helices detected in a minority of L4.5 strains (Figure 7G). Additionally, RDe in L4.4cn led to a loss of about 500 amino acids from the C‐terminal region of *PE_PGRS50* and caused the downstream *PE_PGRS49* to lose most of its sequence starting from the N‐terminal (Figure 7E).

## 3 DISCUSSION

Our analysis provided new insights into the eco‐evolutionary dynamics of MTBC in China, integrating historical transmission routes with adaptive mechanisms. While paleopathological evidence indicated ancient overland introductions (~4000–2000 years old) [8, 9], our phylogeographic analysis of 23,873 MTBC's genomes suggested medieval maritime transmissions (1000–400 years old) might have contributed significantly to pathogen distribution patterns, consistent with the models of trade‐associated dispersal [36]. These precolonial introductions of MTBC appear more extensive than previously estimated in colonial‐era frameworks [6], with genomic parallels to Southeast Asian transmission processes [12]. The limited lineage diversity of MTBC observed in China, previously attributed to population bottleneck effects [7, 37], appears to find complementary explanation through sustained selection favoring local MTBC clades under macrophage‐driven selection pressures. We explored preliminary approaches to detect adaptive evolution signals in MTBC, a pathogen characterized by highly conserved genomes [11, 23], through the integration of neutral mutation simulations and analyses of genomic information entropy variations. This methodology seeks to partially mitigate limitations associated with conventional prokaryotic genome analysis methods, such as the dN/dS [38], while incorporating evolutionary considerations of synonymous mutations that are often underemphasized. Our findings suggested some local clades of four MTBC sub‐lineages likely underwent macrophage‐driven evolutionary adaptation enabling sustained dominance in Chinese host populations, even as European colonial expansion (the 19th–20th century) facilitated global dispersion of other MTBC lineages or clades [11, 39]. Our results further proposed a correlation‐based hypothesis that historical cold periods and wars may facilitate MTBC expansion in China, consistent with evolutionary patterns observed in other pathogens [40, 41]. These findings align with growing recognition of regional host‐pathogen coevolution [22, 42], offering complementary perspectives to conventional dispersal‐centric explanations.

### Precolonial MTBC transmission between China and West Europe

The phylogenetic analysis revealed four circulating MTBC sub‐lineages in China with distinct historical dissemination patterns shaped by possible maritime trade routes, colonial activities, and geographical isolation, enabling the localized expansion of specific clades. Four MTBC sub‐lineages were detected in China. Of these, L2.2 has two most probable source regions, namely Fujian and Anhui, both provinces in South China (Figure S5A). Fujian, a coastal province, was home to the Quanzhou port, one of the world's largest and most prosperous ports roughly 1000 years ago. Geographically, Anhui is located relatively close to Fujian, suggesting that L2.2 might enter China through maritime trade about 1000 years ago, which is consistent with Liu et al.'s findings [7]. We clarified the early dissemination pathways of the most prominent globally expanding clades, L2.2d and L2.2e (alternatively classified as L2.3.5 and L2.3.6 [43]), which exhibit high transmission efficacy, virulence, and drug resistance [10, 43]. These sister clades were estimated to have diverged in South China approximately 300 years ago. While L2.2d remained localized in China, L2.2e spread to Western Europe and subsequently dispersed worldwide. It underscored the critical role of historical pathogen transmission routes between Western Europe and China in shaping contemporary global distribution patterns and transmission dynamics of MTBC.

On the other hand, the lineage L4 was generally thought as originating in Europe [11, 39]. Interestingly, the results highlighted the most probable primary source region for L4.2 as the United Kingdom, while for L4.4, it was most likely the Netherlands (Figure S5B,C). Both of these countries were key participants in major maritime exploration and global colonial activities, which probably contributed to the widespread transmission of L4.2 and L4.4 across the world in the last 200 years (Figure 2C–F), which is consistent with observations of other L4 sub‐lineages [39]. The results showed that L4.4 was introduced from Western Europe to Southeast Asia and then to China. This connection between Southeast Asia and China in the spread of L4.4 was also supported by other recent work [12]. Additionally, the most probable source region for L4.5, which is considered a China‐specific sub‐lineage [7], is Zhejiang, a coastal province in South China (Figure S5D). Roughly 700−400 years ago, Zhejiang was a significant area in maritime trade in China. During this period, L4.5 may have spread from South China to Western Europe, most probably via maritime trade. The Qinghai‐Tibet and Mongolian Plateaus geographically isolated China, forming an independent unit that may restrict the continued overland introduction of MTBC, as seen in studies on Greenland and Denmark [44]. Consequently, MTBC likely entered South China sporadically through the limited coastal regions via maritime human activities, potentially resulting in a low lineage diversity due to the restricted number and frequency of introduction events through these geographical barriers. Furthermore, this isolation may have prevented the sustained introduction of MTBC from the adjacent colonial regions, such as India and Vietnam, during the Western colonial period [39], providing the opportunities for the expansion and adaptive evolution of the earlier‐arrived four MTBC sub‐lineages within China.

### MTBC expansion in China temporally aligned with cooling and warfare

Historical effective population size estimation revealed that the local MTBC clades established the endemic transmission in China at least 450 years ago, with three subsequent population expansions temporally associated with the regional climatic cooling events and widespread civil conflicts, which potentially collectively drove the adaptive evolution through the rapid transmission and population expansion in local hosts. The three population expansions of local MTBC clades corresponded to the lowest temperature periods of the South, North, and Southwest China over the last 1000 years (Figure 3A). Although the direct evidence that the cooling periods have directly driven the expansion of MTBC populations still lacks, this notable correlation is unlikely just coincidental. Low temperatures, compared to other climatic factors, such as humidity and air quality, are more likely to prolong the survival of pathogens in the environment and affect the spread range of pathogens [45], impact human immunity and reduce social distancing among people [46], and decrease crop yields leading to more people being displaced due to famine, which might favor the outbreak of respiratory infectious diseases. Additionally, we found that the two most significant population expansions of MTBC in China followed the periods of the Ming dynasty's downfall (A.D. 1600–1644) and the Taiping civil war (A.D. 1851–1864) in the Qing dynasty (Figure 3B). It is reasonable to hypothesize that during these periods, extensive and prolonged wars caused substantial reductions in the Chinese population, and after these wars, the weakened health infrastructure and management system may have struggled to support the recovering human population, likely promoting the spread of MTBC. This coincides with previous findings that both the First World War and the collapse of the Soviet Union facilitated the expansion of MTBC in Europe [20]. It must be emphasized that the observed correlation between MTBC population expansion and historical cooling periods or warfare remains as per statistics, and lacks the direct causal evidence. In future experimental investigations, we will systematically test the survival and transmission dynamics of MTBC under the controlled low‐temperature conditions. Moreover, the widespread use of antibiotics in tuberculosis treatment since the mid‐20th century may have substantially altered MTBC population dynamics; therefore, analyzing recent trends was excluded from this study. Nevertheless, we propose a working hypothesis: following the historical formation of local clades of the four sub‐lineages in China, both regional cooling and warfare likely promoted the population expansion of these clades, which might subsequently enable their establishment in the novel host environments thus facilitated their adaptive evolution.

### MTBC adapted in China via macrophage pressures during recent centuries

The findings above elucidated the historical introduction, dissemination, and population expansion of the currently predominant MTBC sub‐lineages circulating in China. Nevertheless, the successful transmission and rapid population expansion of a pathogen within a single host population usually require adaptive evolution to the host. We therefore investigated whether such evolutionary mechanisms contributed to MTBC dominance in China. Our findings suggested that the adaptive evolution of local MTBC clades in China, characterized by the selection pressures on macrophage‐resistance genes and T cell epitopes, possibly facilitated their sustained dominance and geographic expansion through their enhanced adaptation to host immune responses. We aim to explain why local clades in China persist and exclude subsequent lineages. Long‐term MTBC‐human coexistence may have led to differential transmissibility [42], suggesting that adaptive evolution might enable local clades to dominate and spread widely across China. Firstly, the results of the genetic diversity analysis showed that the local clades underwent activate evolutionary processes following their formation (Figure 1B–E). We observed a significant overall increase in mutation rates from about 300 years ago of MCCs (Figure 1K–N), which aligned with their rapid population expansion and spread centered around South China. However, the dN/dS values suggested an overall purifying selection on the whole genome of these MCCs, consistent with the conservative nature of MTBC [11, 23]. Therefore, it is necessary to identify the specific genes related to adaptive evolution and potential sources of selection pressures. Therefore, we calculated and compared the selection pressures experienced by MCCs and MCNCs across the 15 key functional gene groups, as well as T and B cell epitopes, assuming neutral mutation simulations (Figures 4 and S7). The results indicated that all MCCs experienced stronger positive selection pressures on the gene group MIKGs, the key genetic determinants enabling MTBC to resist macrophage attacks, and these pressures significantly exceeding those experienced by the MCNCs (Figure 4M). Additionally, the MCCs tended to select optimal codons in synonymous mutations on MIKGs compared to the MCNCs (Figure S7A), which may reflect a distinct evolutionary strategy. This suggested consistent selection pressures on the local MTBC clades in China from host macrophages. Although the T cell epitopes were generally under purifying selection, a finding supported by previous studies [47], the MCCs consistently demonstrated a stronger inclination for non‐synonymous mutations and a higher genetic diversity compared to the MCNCs (Figure 4N–U). Considering that the T cell epitopes bind to HLA molecules to induce an immune response, and human populations with similar genetic backgrounds show similar HLA allele distributions [27], the MCCs' consistent characteristics likely reflect their adaptive evolution in the Chinese population. The underlying mechanisms for the inconsistent patterns between T cell and B cell epitopes, and whether epitope convergence across clades reflects the regional patterns of the host genetic diversity, remain to be explored in future studies.

As a control group, the EGs were under purifying selection in all the clades (Figure S7B), aligning with the consensus that the EGs are highly conserved [33]. The absence of consistent characteristics in the EGs of MCCs or MCNCs further confirmed that the observed consistency of the MIKGs and T cell epitopes was not a result of phylogenetic influences. Moreover, for the local clades of L2.2, L4.2, and L4.4 in China, the adaptive evolution appeared to be relatively recent compared to their diversification, as strong positive selection pressures were observed only about 200 years ago on both the MIKGs and T cell epitopes (Figure 4E–L). Additionally, both showed synchrony, further supporting this notion. This timeline coincides with estimates made of the recent rapid spread of these sub‐lineages throughout China. It implied that their ability to establish in new host environments and rapidly expand within the Chinese population might be facilitated by their adaptive evolution to counteract the host immune response. The sub‐lineage L4.5 may have adapted to the Chinese population around 600 years ago (Figure 4H,L), which might explain why L4.5 has remained confined to China and did not become pandemic in Western Europe after its introduction about 600–400 years ago (Figure 2G). The distinctive adaptation of local MTBC clades to the Chinese population, combined with China's relatively limited exposure to demographic disruptions during the Age of Exploration and colonial expansion, may have jointly contributed to the low lineage diversity of MTBC in China observed. This pattern exhibits remarkable contrast to the evolutionary dynamics observed in Europe and several neighboring countries around China [11, 12, 13, 14].

### DNA information entropy dynamics revealed MTBC adaptive genes in China

Our analysis identified the adaptive evolution signals in MTBC local clades through DNA information entropy dynamics, pinpointing 12 genes possibly associated with macrophage survival and immune evasion. Experimental validation demonstrated that *Rv0801*, exhibiting the most pronounced adaptive evolution signals, significantly enhanced the intracellular persistence within human macrophages. We identified potential adaptive evolutionary sites based on DNA information entropy changes of the genes located before and after mutations, assuming that genomes maintain entropy stability to avoid collapse from random mutations [34]. Simulations showed that random mutations tend to increase the information entropy, while fixed mutations in microbial populations often result in smaller increases or even decreases in information entropy (Figure 5E). However, there were mutations with significant entropy increases that were preserved (Figure 5E,F), and as they probably enhanced fitness, so microbial populations were forced to retain them as the lesser of two evils. Using this method, we identified mutations that were both shared and fixed in the MCCs and that caused greater deviations and entropy increases when compared to the MCNCs. Genes with such mutations may thus be related to the adaptive evolution of MTBC in China. A total of 12 genes were identified that not only had high KL values but also had the KL values that were higher in each MCC when compared to the corresponding MCNC (Table S1). Therefore, these genes may represent a shared adaptive characteristic of these local clades in China. A literature review revealed that at least half of these genes were associated with MTBC's ability to invade macrophages, survive within them, or evade their immune defenses (Tables S1, S2). Interestingly, the gene *lldD2* has been proven to be associated with the adaptation to the human lung environment [48]. These genes have also exhibited either a high positive selection or a strong bias for optimal codon to rare codon transition mutations (Table S1), offering another perspective on the potential selection pressures faced by MTBC within the Chinese population, particularly selection pressures from host macrophages. Additionally, it should be noted that repetitive or hypermutable regions (e.g., CpG islands, INDELs) may carry a risk of confounding adaptive evolution signals derived from DNA information entropy dynamics; however, such regions were not detected in these 12 genes identified of local MTBC clades in China.

Among these 12 gene, the *Rv0801* gene exhibited the most pronounced evolutionary characteristics in all MCCs (Figure 5A–D,F). It is a non‐essential gene for the in vitro growth of MTBC [33], encoding an unknown function protein. To preliminarily explore the function of this gene, *Rv0801* was heterologously expressed in Msm, as it is phylogenetically close to MTBC, and a kind of non‐pathogenic *Mycobacteria* commonly used as an accepted surrogate in experiments. Msm typically does not cause human diseases and is rapidly killed in in vitro cultured macrophages [49], yet our experimental results showed that the protein encoded by *Rv0801* significantly enhanced the survival rate of Msm in macrophages (Figure 5G,H). Moreover, *Rv0801* was predicted to encode a putative glyoxalase I, which represents the rate‐limiting step for the detoxification of methylglyoxal [50]. Notably, MTBC‐infected macrophages produce methylglyoxal that induces apoptosis, activation, and immunity [51], and *Listeria monocytogenes* uses glyoxalase to detoxify methylglyoxal, enhancing its survival in macrophages [52]. Thus, *Rv0801* could potentially enhance the survival of Msm or MTBC within macrophages via an analogous mechanism, which we will explore in future studies.

### Structural variations revealed MTBC adaptation in China under macrophage pressures

The findings suggested that the structural variations in *PE_PGRS* family genes, particularly convergent INDELs and lineage‐specific RDs, may reflect the adaptive evolution in MTBC under host immune pressure and macrophage‐mediated selection, potentially influencing their pathogenicity and immune evasion strategies. Firstly, we identified a shared feature among all four MCCs, in that specific regions in their *PE_PGRS* family genes were with concentrated INDELs. The frequency of INDEL occurrence in these regions is significantly higher in the MCCs than in the MCNCs and other lineages (Figures 6A–H and S10–12). Although the short‐read sequencing detection of INDELs in repetitive *PE_PGRS* family genes may raise the false‐positive concerns, the reliability of our findings is supported by two methodological safeguards. Firstly, the use of conservative INDEL identification parameters minimized technical artifacts. Secondly, the observed clade‐specific clustering pattern, where INDEL accumulation occurs exclusively in particular local clades in China compared to other MTBC clades, would not be appropriate if technical artifacts were predominant. Such artifacts would produce uniform INDEL clustering across all lineages, which was, however, not observed in this study. In MTBC, the *PE_PGRS* gene family contains at least 65 genes [53], which are unique to *Mycobacterium*, with their encoded proteins located on the outer membrane and closely linked to mycobacterial pathogenesis [54]. And the structural variations associated with strain surface and immune signaling may play an important role in the evolution of MTBC [55]. However, previous studies found that *PE_PGRS* family genes were highly conserved [56], and our results supported this view (Figure S9E–H), indicating that the selection pressures on these genes cannot be assessed through mutations. Interestingly, we found that, although there were many structurally similar PG_II_ helices, which are special repeat structures in the PGRS domain [54], in the genes *PE_PGRS3*, *PE_PGRS4*, and *PE_PGRS28*, the INDELs for strains of the MCCs tended to concentrate on a single PG_II_ helix (Figure 6E–H). In *PE_PGRS3*, most strains of the MCCs had INDELs on the PG_II_ helix closest to the C‐terminal domain. These INDELs mostly led to frameshift mutations, likely affecting the normal protein structure of the C‐terminal domain located downstream of these INDELs (Figure 6E). Interestingly, *PE_PGRS3* has a unique C‐terminal domain that is surprisingly long (about 80 amino acids, compared to the typical 5–20 amino acids in most genes of *PE_PGRS* family [54]) and is rich in arginine. This domain is important for adhering to macrophages [57], and may influence bacterial entry into macrophages as well as their persistence in low‐phosphate cytoplasmic environments [54, 58]. However, due to the lack of functional studies on *PE_PGRS4*, *PE_PGRS28*, and *PE_PGRS17*, we could not infer the influence of INDELs in these three genes on the strains' survival. Considering that the PGRS domain is the target of the host humoral response in patients [54], these INDELs might also affect immune evasion strategies for strains of the MCCs, implying a possible selection pressure from the host immune response.

Moreover, this study identified five clade‐specific large segment deletions (RDa‐e) within the MCCs (Figure 7A–H). Previous studies supported that some large segment insertions and deletions (usually called RvDs and RDs) were related to the virulence or drug resistance of MTBC [59]. Our previous work showed an absence of RvDs across the four sub‐lineages [60], thereby directing this study's focus towards RDs. The prevalence of RDa in L4.5, which was the most representative MCC, resulted in the deletion of *Rv0576*, which encodes a potential *ArsR* family transcription factor that may regulate *Rv0577* [61]. The *Rv0577* gene is essential for dendritic cell maturation and Th1 immune response activation and is critical for initiating host immunity [62]. Additionally, the protein encoded by *Rv0576* was predicted to have a mycothiol‐dependent enzyme structure in its tail. Given that mycothiol aids in detoxifying reactive oxygen and nitrogen species produced by macrophages [63], *Rv0576* could contribute to MTBC survival in macrophages. The remaining four RDs mostly affected the other five genes of the *PE_PGRS* gene family. RDb‐d led to the loss of PG_II_ helices in these genes (Figure 7F–H). This loss could reduce the initiation of TNF‐α production [64], a tumor necrosis factor secreted by macrophages. At least in the cases of *PE_PGRS9* and *PE_PGRS48*, the loss of PG_II_ helices may significantly alter the structure of regions abundant in hydrophobic amino acids, potentially disrupting the normal cell wall attachment of their encoded proteins [65]. In addition, one of the two genes impacted by RDe, *PE_PGRS49*, is nonfunctional [53]. In contrast, for *PE_PGRS50*, RDe in L4.4cn caused the loss of approximately 500 amino acids from its C‐terminal (Figure 7E), including a specific 80‐amino‐acid arginine‐rich C‐terminal domain homologous to that in *PE_PGRS3* mentioned above [54], and possibly serving the same purpose. Thus, in the MCCs, the observed tendency of structural alterations or deletions in homologous structures of both *PE_PGRS3* and *PE_PGRS50* gene that may both be involved in macrophage adhesion, suggested that this tendency was not coincidental but rather in response to a specific selection. Although the functional changes in genes influenced by the five RDs require further exploration, the findings discovery of these unique RDs also suggested that MCCs might exhibit adaptive evolution under the selection pressure of macrophages in China.

### Limitations and future research perspectives

Despite our efforts to collect the genomes of the targeted four sub‐lineages globally to estimate their historical spread and the genomes of all the nine MTBC lineages for ancestral reconstruction and phylogenetic analysis, unsurprisingly, this study could not fully reconstruct the historical exchange routes of MTBC between Europe and China via maritime trade due to a lack of strains from intermediate regions, particularly the Middle East and Central Asia. Expanding strain collections and genomic surveys, especially in regions with frequent human population exchanges and demographic shifts, will offer valuable insights. Moreover, despite employing multiple tools and conservative parameters for structural variation identification, along with verifying the presence of RDs through sequencing read coverage and confirming that INDEL clustering in the target genes was not attributable to sequencing technology by comparison with other MTBC lineages, we will validate these structural variations by long‐read sequencing and experimental approaches in future studies. And the generalizability and robustness of the newly developed methods for comparing selection pressures in prokaryotic organisms and identifying genes undergoing adaptive evolution will be further validated through application to additional biological populations. Finally, this study was focused on MTBC itself and did not explore how human genetic variations influence MTBC adaptation, which will also be addressed in the future work.

## 4 CONCLUSION

In conclusion, the current study established a comprehensive framework for understanding how MTBC emerged in China through recurrent transcontinental introductions and subsequent adaptive evolution by reconstructing the population history and detecting the adaptive evolution signals. We developed an integrated analytical framework for prokaryotic population adaptive evolution studies that combines lineage dynamics, historical mutation rate estimation, neutral mutation simulations, DNA information entropy analysis and genome structural variation analysis. The novel methods enable quantification of selection pressures and identification of adaptive evolutionary signatures in genes, thereby elucidating the host adaptation mechanism of local MTBC clades in China. Experimental validation through recombinant strain infection confirmed that *Rv0801*, a key adaptive gene of local MTBC clades identified through this framework, significantly enhances intracellular survival capacity within human macrophages. We suggested that the geographic isolation in genetically homogeneous host populations synergized with post‐warfare demographic recovery and temperature‐driven epidemic cycles, enabling the local MTBC clades to dominate through macrophage‐targeted evolutionary adaptations. The distinctive transmission and evolutionary trajectory of MTBC in China reveals how regional coevolution reshapes the global biogeography of infectious pathogens, supplementing the classical paradigm of unidirectional pathogen dissemination during colonial‐era expansions. These findings establish a multidisciplinary model for reconstructing the historical disease spread while providing novel analytical frameworks to predict pathogen's local adaptation in evolving epidemiological contexts.

## 5 METHODS

### Data collection

WGS Data. First, we downloaded the relevant original sequencing reads from NCBI and gathered details about MTBC's WGS data from published publications. For the obtained WGS data, sequencing coverage was determined in Bamdst (version 1.0.9) (https://github.com/shiquan/bamdst), and those samples with coverage below 90% were excluded. Furthermore, these WGS data were retyped using TB‐profiler (version 4.1.1) [66], eliminating strains whose typing results could not be precisely assigned to a single sub‐lineage (possibly mixed DNA samples). After filtering, we retained WGS data for 23,665 MTBC strains, 6804 of which carried explicit geographic tags. Additionally, 228 MTBC isolates from patient sputum samples were collected from Sichuan province in Southwest China during 2015–2020, from which whole‐genome DNA was extracted, and then sequenced using the HiSeq. 2000 platform. After filtering, WGS data for an additional 208 MTBC isolates were included in this study. All WGS data were obtained using Second Generation Sequencing.

Genotyping Results. We collected 5793 genotyping results from all the Chinese provinces except Hong Kong and Macao, and 12,877 genotyping results with explicit geographic tags from published articles from other countries around the globe. Some of these results were directly based on phylogenetic lineages derived from the WGS data, while others were from spoligotyping results. Given that spoligotyping results are generally concordant with lineage classifications based on WGS data, these spoligotyping results were assigned to the relevant MTBC lineage according to previously identified links between spoligotyping results and phylogenetic lineages [16].

TB Case Data. The Data‐center of China Public Health Science (https://www.phsciencedata.cn/Share) and the Taiwan Tuberculosis Control Report (https://www.cdc.gov.tw/InfectionReport/List/uKmf00HvSmkNaX9lNY-raQ) provided the yearly number of new cases and TB incidence rates for every province in China between 2016 and 2018.

Epitopes. Experimentally confirmed human T cell and B cell epitope sequences of MTBC were retrieved from the Immune Epitope Database (https://www.iedb.org) and filtered using the methodology of Stucki et al. [11]. Finally, duplicate epitopes were merged, resulting in 1276 T cell epitopes and 380 B cell epitopes.

Gene groups. In reference to previous studies [29, 67, 68], we gathered identified genes of MTBC linked to the following: energy metabolism, central carbon metabolism, nitrogen metabolism, amino acid biosynthesis, virulence factors, stress, macrophage infection, virulence factors, cell envelope formation, and the metabolism of cofactors and vitamins. As a control, 460 essential genes (EGs) required for in vitro growth were also identified [33].

### Spatial analysis

Based on the typing results and corresponding geographic tags obtained from the “Genotyping Results” section, the global distributions of L2.2, L4.2, L4.4, and L4.5 were estimated using the Inverse Distance Weighted Spatial Analysis (Interpolation) in ArcMap (version 10.8.1) [69]. Additionally, ArcMap was also used to visualize the distribution ratios of the MTBC sub‐lineages and the incidence rate across various provinces in China.

### SNPs and INDELs calling

For the WGS data, quality control was performed using fastp (version 0.20.1) [70], followed by mapping the sequencing reads to the reference genome H37Rv (NCBI assembly number: GCF_000195955.2) with bwa‐mem2 (version 2.2.1) [71]. The PCR duplicates were marked using the “MarkDuplicates” module of GATK (version 4.1.7.0) [72], while samtools (version 1.10) [73] was used for sorting and deduplication. GATK was used for variant calling, which included finding SNPs and INDELs, with hard filtering applied through the “VariantFiltration” module. The hard filtering criteria for SNPs were: “QD” < 2.0, “SOR” > 3.0, “FS” > 60.0, “MQ” < 40.0, “MQRankSum” < −12.5, and “ReadPosRankSum” < −8.0. The hard filtering criteria for INDELs were: “QD” < 2.0, “SOR” > 10.0, “FS” > 200.0, and “ReadPosRankSum” < −20.0. Finally, we excluded all SNPs and INDELs located in direct repeat sequences (e.g., phage sequence insertion or mobile genetic elements) that were challenging to characterize with short‐read sequencing technologies. To further eliminate potential false‐positive INDELs, sequencing coverage gaps on each genome were identified using Bamdst (version 1.0.9) (https://github.com/shiquan/bamdst).

### Phylogenetic construction

The SNPs from the whole genome with a missing rate higher than 20% (possibly due to insertions or deletions, low coverage or mapping quality at those sites) were filtered out, and the identification of missing regions was conducted using Bamdst (version 1.0.9) (https://github.com/shiquan/bamdst). The filtered SNPs from all strains intended for the same phylogenetic tree were concatenated into a sequence of equal length, where invariant sites were complemented, based on the reconstructed ancestral whole‐genome sequence of MTBC. From these sequences, phylogenetic trees were constructed for the L2 strains and L4 strains, including all known sub‐lineages with their corresponding geographic tags, and with the outgroup L1. These two phylogenetic trees were constructed by IQ‐Tree (version 2.2.2.6) [74], with the Site Model “GTR + G”, as the best model estimated by “ModelFinder” module [75]. The number of bootstrap replicates was set to 200. Phylogenetic trees were visualized with iTOL (version 6.9.1) [76]. To display the phylogenetic tree's topology in illustrations, branch lengths for the outgroup were artificially shortened. In addition, to reconstruct the ancestral whole‐genome sequence of MTBC in subsequent sections, a phylogenetic tree was drawn based on 1407 MTBC strains, including all the main sub‐lineages of all the human‐adapted MTBC lineages (L1‐9) reported to date. The tree excluded lineages adapted to animals, and used *Mycobacterium canetti* as the outgroup. Based on the phylogenetic tree of a strain data set including all the MTBC sub‐lineages and the concatenated SNP sequences mentioned above, the ancestral sequence reconstruction of the most recent common MTBC ancestor at these variable sites was conducted using TreeTime (version 0.11.0) [77]. Invariant sites were complemented using the reference genome H37Rv, thus producing the reconstructed ancestral whole‐genome sequence of MTBC. Based on the phylogenetic trees of all samples within each clade, the same method was applied to separately obtain the reconstructed whole‐genome sequences of the most recent common ancestors for samples within the L2.2d, L2.2e, L4.2b, L4.2cn, L4.4a, L4.4cn, L4.6789, and L4.5.

Genetic diversity analysis. Using a custom Python script, random sampling was conducted on the target strain population with an increasing number of samples in gradients. The Shannon, Simpson, and Pielou's evenness indices were computed for SNPs on the whole‐genome CDSs or T cell epitopes of strains for each of the 20 repetitions made for the same gradient. Ultimately, we obtained the diversity index dilution curves that depict how these indices change with the increasing number of random samples. This method is used to characterize the genetic diversity of mutations within a target strain population.

### Bayesian‐based coalescent and phylodynamic analysis

Dating Analysis. The Bayesian coalescent‐based algorithm provides a robust framework for estimating the lineage divergence time in MTBC. Phylogenetic construction, calculation of divergence times for each node, and estimation of spread between different geographical regions were conducted for L2.2, L4.2, L4.4, and L4.5 strains with geographical tags using BEAST2 (version 2.6.6) [78] based on concatenated genome‐wide SNPs. The site model utilized was “GTR + G4,” the clock model used was “relaxed clock log normal,” and the demographic model (tree prior) applied was “coalescent Bayesian skyline.” The MRCA prior was referenced from Sabin et al.'s research [5]. For each sub‐lineage, two chains of 2 × 10^8^ generations were run, and sampled every 10,000 to ensure independent convergence of the chains, with the first 20% discarded as burn‐in and combined by LogCombiner (version 1.10.4) (https://beast.community/logcombiner). Tracer (version 1.7.2) was used to assess convergence (https://github.com/beast-dev/tracer/releases/tag/v1.7.2), while ensuring that the vast majority of relevant parameters exceeded an effective sample size of 200.

Bayesian Phylodynamic Analysis. Based on the results of the dating analysis, historical migration routes, corresponding times and directions were reconstructed for L2.2, L4.2, L4.4, and L4.5 using SpreaD3 (version 0.9.7) [79], and filtering out migration routes with a posterior <0.99. For analysis convenience, the relevant geographical regions were divided into four major areas in China (North China, West China, Southwest China and South China) along with North America, South America, Africa, West Europe, East Europe, North Europe, Oceania, West Asia, South Asia, Central Asia, Southeast Asia, and Northeast Asia (strains from China were no longer duplicated in any of the Asian geographic regions). These routes by which MTBC spread, as well as the times and directions of spread were visualized through ArcMap (version 10.8.1) [69]. The results indicated two main migration centers, and the spread routes for each sub‐lineage were divided into two categories, focusing on South China and Western Europe as the primary migration centers for separate illustrations. The oldest migration routes were also noted.

BSP analysis. Using the same parameters as in the dating analysis, BSP analysis estimated the past effective population size dynamics for each clade of four MTBC sub‐lineages (L2.2, L4.2, L4.4 and L4.5). However, it is important to note that the number of isolates sequenced by WGS collected for each sub‐lineage was not proportional to their epidemiological prevalence, with some sub‐lineages being oversampled while others were undersampled [7]. To reduce this bias, redundant samples from the same country (excluding China) or the same province of China, which were phylogenetically similar, were filtered out, ultimately resulting in a WGS data set of 1405 samples. This filtering method follows the approach of Liu and his colleagues [7]. To avoid biases caused by population structure, we performed separate analyses for each independent clade. Thus, the BSP analysis was conducted on each clade of MTBC from this data set, with the tree height from the dating analysis corresponding to the ages of the most recent common ancestors of the clade. The effective population changes data over time generated by the BSP analysis was converted into a curve of effective population growth rate changes over time using the formula:

$$r=\left(N_{t}-N_{0}\right)/t.$$

In this formula, *r* represents the growth rate of the effective population, *N*_0_ represents the initial effective population size, and *t* represents the time taken for the effective population to change from *N*_0_ to *N*_*t*_.

Along with historical Chinese population data and historical reconstructed temperature data for various Chinese regions, we also gathered historical epidemic outbreak records from ancient Chinese literature. These data were subjected to a comparative analysis with the growth rate of the effective MTBC population over time. The epidemic outbreak data were aggregated on a 50‐year timescale, counting the total number of provinces with recorded outbreaks every 50 year, and a curve was plotted to show the variation over time.

### Source tracking analysis

Using the algorithm of FEAST (version 0.1.0) [80], we traced the potential geographic origins of strains based on whole‐genome SNP data. First, we tallied the number of SNPs within each CDS across all strains within the four relevant sub‐lineages (L2.2, L4.2, L4.4, and L4.5). We obtained a regional SNP characteristic profile for FEAST by averaging the SNP counts per CDS for strains from the same region to prevent bias caused by different strain numbers from different regions. Next, we used the results from the “Bayesian Phylodynamic Analysis” to set the “source regions” and “receiving regions.” For L4.2 and L4.4, selected countries in Western Europe were designated as the “source region.” For L2.2 and L4.5, certain provinces in South China were defined as the “source region,” while other countries and provinces from other regions in China were considered “receiving regions.” Using this method, we determined the putative “source region” for the strains belonging to any of the four sub‐lineages. The results were visualized as chord diagrams using the R package “circlize” (version 0.4.16) [81].

### Historical mutation rate and selection pressure estimation

Using the phylogenetic trees with node divergence time constructed in the section “Bayesian‐based Coalescent and Phylodynamic Analysis,” the ancestral sequences for each node were obtained using the “ancestral sequence reconstruction” method in TreeTime (version 0.11.0) [77]. A Python script was developed to align each node's divergence time with its corresponding reconstructed sequence, obtaining SNPs for each node's reconstructed sequence compared to its parent node's. After that, these SNPs were split equally between the parent and child nodes for every year. The average number of SNPs per year was tallied from the root node to the tips, facilitating the calculation of the annual average mutation rate. Moreover, the timeline from the root node to the present was divided into 20 intervals, within which the dN/dS and dR/dO values were calculated for each interval, ultimately estimating the annual mutation rate, dN/dS, and dR/dO over time from the origin to the present for each target clade. This methodology was applied to all the genes in the whole genome, the T cell epitopes, and MIKGs, respectively, calculating the annual mutation rate and selection pressures from the origin of all the target clades to the present.

### Quantifying selection pressure analysis via neutral mutation simulation

The value of dN/dS (also known as Ka/Ks) aims to calculate the magnitude of selection pressures by determining the ratio of non‐synonymous to synonymous mutations. To address the biases inherent in traditional selection pressure estimation via dN/dS and the difficulty of cross‐population comparisons, we employed the neutral mutation simulations to correct the selection pressure estimates. Firstly, referring to the method used in previous studies [82], we estimated the dN/dS values of strains in each target clade by use of a custom Python script. The nucleotide substitution model employed was the Tamura model, with all the parameters estimated using a strain set that included all the sub‐lineages of MTBC. To explore potential selection pressures on synonymous mutations, we used a method similar to the dN/dS value to calculate the dR/dO value, which represents the ratio of optimal‐to‐rare codon transitions rare‐to‐optimal codon transitions in synonymous mutations. We also performed Bernoulli trials to simulate random mutations for all the actual mutations in each target CDS, with the success probability being the expected probability of nonsynonymous mutations. This allowed us to statistically compare the differences in selection pressures faced by various clades and gauge the degree to which the actual mutations differ from neutral mutations. In the simulation process, the Tamura model was used as the nucleotide substitution model, and parameters were estimated based on the strain set that included all MTBC strains with WGS data collected in this study. We assumed all mutations occurred independently. This method referred to the study by Trauner et al. [83]. The random mutation simulation was repeated 1000 times with different random seeds, resulting in a simulated data set. Then, we calculated the extent to which the actual dN/dS or dR/dO ratio deviated from the results of the random mutation simulations using the following formulas:

$$D_{k}≔\frac{y-\hat{y_{k}}}{\frac{\sum_{c=1}^{n}\hat{y_{c}}}{n}},$$

$$\left\{\begin{array}{l} {OS}_{k}≔-{\left(1+\tanh\left(D_{k}\right)\right)}^{-\ln\left(D_{k}+1\right)\ln\left(R+1\right)}+1\;\left(D_{k}\geq 0\right)\;\\ {OS}_{k}≔{\left(1+\tanh\left(D_{k}\right)\right)}^{\ln\left(-D_{k}+1\right)\ln\left(R^{-1}+1\right)}-1\;\left(D_{k}<0\right). \end{array}\right.$$

In the preceding formulas, *n* = 1000, represents the total number of random mutation simulations, and *k* = 1, 2, 3, …, *n*, represents the *k*th random mutation simulation, *y* represents the actual dN/dS or dR/dO, and *y*_*k*_ represents the dN/dS or dR/dO from the *k*th simulation. *R* represents the ratio of the mutation rate of target CDSs to that of the whole‐genome genes, whereas *OS*_*k*_ is the *k*th simulation's offset score, which denotes the degree of the actual values that deviate from the simulated values, ranging between (−1, 1), with the sign indicating positive selection or purifying selection, and the magnitude representing the strength of the selection pressures. In the “Results” and “Discussion” sections, dN/dS_*OS*_ or dR/dO_*OS*_ were used to represent the *OS* value of dN/dS or dR/dO, respectively.

### DNA information entropy analysis

Detecting signals of adaptive evolution in conserved genomes poses a challenge. To address this, we developed a new method based on DNA Information Entropy. For a given DNA sequence, if the nucleotides, including A, T, C, and G, and considered signals, along with the frequency *P* of each nucleotide, this data will form an information source [84]. To find genes in a given target clade that experienced a significant increase in information entropy due to mutations based on this information source, we first calculated the relative entropy (*D*), which represents the degree of disturbance to the information source due to mutations, this was done for each CDS in the whole genome in each target clade with following the formula:

$$D\left(P∥Q\right)=\sum_{i=1}^{4}(P_{i}\cdot \left(-\ln Q_{i}\right)-P_{i}\cdot \left(-\ln P_{i}\right)).$$

Since *D* (*P*||*Q*) is non‐negative and does not distinguish between entropy increase or decrease, we also calculated the change in information entropy (*ΔS*) with this formula:

$$\Delta S=-\sum_{i=1}^{4}Q_{i}\ln Q_{i}-(-\sum_{i=1}^{4}P_{i}\ln P_{i}).$$

*Q*_*i*_ represents the nucleotide frequency of the target CDS (*i* = 1, 2, 3, and 4, corresponding to A, T, C and G, respectively), and *P*_*i*_ represents nucleotide frequency of the same CDS of the reconstructed ancestral reference sequence.

Based on the sign of *ΔS*, we defined KL as follows:

$$\left\{\begin{array}{l} \mathrm{KL}≔D\left(P∥Q\right)\left(\Delta S\geq 0\right)\\ \mathrm{KL}≔-D\left(P∥Q\right)\left(\Delta S<0\right) \end{array}\right..$$

The sign of KL indicates whether there is an increase or decrease in information entropy, while the magnitude of the absolute value indicates the size of the relative entropy.

Next, to identify genes with significantly higher KL in all the MCCs when compared to their corresponding MCNCs, the following formula was used:

$$\left\{\begin{array}{l} {\mathrm{MKL}}_{\mathrm{MCC}}≔\frac{{\mathrm{KL}}_{\mathrm{MCC}}-{\mathrm{KL}}_{\min}}{{\mathrm{KL}}_{\mathrm{MCNC}}-{\mathrm{KL}}_{\min}}\\ {\mathrm{MKL}}_{\mathrm{MCNC}}≔\frac{{\mathrm{KL}}_{\mathrm{MCNC}}-{\mathrm{KL}}_{\min}}{{\mathrm{KL}}_{\mathrm{MCC}}-{\mathrm{KL}}_{\min}} \end{array}\right.,$$

where KL_min_ is the minimum value of KL across all genes in the whole genome in a specific MCC and its corresponding MCNC, whereas KL_MCC_ represents the KL of the MCC, and KL_MCNC_ represents the KL of the corresponding MCNC.

For each MCC, genes with KL_MCC_ and MKL_MCC_ > 1 that were shared across all four MCCs was selected first. Then, genes with significantly high KL_MCC_ and MKL_MCC_ values that were shared across all four MCCs were selected, with the criterion being that both the KL_MCC_ and MKL_MCC_ values ranked in the top 20% of all the selected genes and across all four MCCs. Then, the functions of the selected genes were clarified through a literature review. The dN/dS_*OS*_ and the dR/dO_*OS*_ values for each selected gene were also calculated with the same method mentioned in the “Quantifying Selection Pressure Analysis via Neutral Mutation Simulation” section.

We merged all the samples from the four MCCs into a sample set to test the hypothesis that mutations that result in appreciable entropy increases in the genome sequence are typically preferentially eliminated by biological populations in the real world. For this set, across every CDS in the whole genome, we conducted 100 random mutation simulations, each based on the same number of actual mutations. We calculated the average KL value of all these simulations and compared it to the actual KL value for each CDS.

### Recombinant strain construction

The restriction enzyme sites of *Rv0801* were identified, selecting *Bam*H I and *Cla* I as the restriction endonucleases. Primers were designed using Primer Premier 5 (version 5.5.0) [85], and a PCR amplification was performed (see Table S3 for more details). The PCR products were verified by 1% (w/v) nucleic acid gel, and the target gene fragments were retrieved and purified. Then *Bam*H I and *Cla* I were used to digest the pALACE plasmid at 37°C for 4 h, with the enzyme digestion system shown in Table S4. The digestion product was purified and ligated with *Rv0801* in a water bath at 50°C for 15 min, and the ligation system is shown in Table S5. The construction of the recombinant plasmids utilized the seamless cloning method. *Escherichia coli* DH5α and wild‐type Msm competent cells were grown fresh in the lab. The recombinant plasmid was then transferred into DH5α competent cells by heat shock transformation. Colonies were selected for positive screening and grown in culture. Sanger sequencing was used to confirm that the *Rv0801* in the recombinant plasmid had no mutations. Electroporation was then used to introduce the same recombinant plasmid into the wild‐type Msm‐competent cells. After colonies were chosen, they were cultivated and PCR‐confirmed.

### Intracellular survival analysis

Macrophage Cell culture. (1) THP‐1 Cell Recovery: THP‐1 cells were thawed quickly in water at 37°C. Then, the cells were slowly added into a centrifuge tube containing 5 mL RPMI 1640, centrifuged at 1000 rpm for 5 min, and the supernatant was then discarded. A solution of RPMI 1640 medium containing 20% (v/v) fetal bovine serum (FBS) (10% (v/v) FBS was used after cell recovery), penicillin (100 U/mL)‐streptomycin (100 μg/mL) mixture, and glutamine (2 mM) was added to the centrifuge tube containing the cells. After the THP‐1 cells were uniformly dispersed, they were transferred to T25 cell culture flasks and then cultured at 37°C in an incubator with 5% CO_2_ and 95% air. (2) Cell Passage: THP‐1 cells were transferred to a centrifuge tube, centrifuged at 1000 rpm for 5 min, and the supernatant discarded. Then, 2 mL of cell culture medium was added, and the THP‐1 cells were uniformly dispersed. The cell suspension was expanded and cultured at a ratio of 1: 2. (3) Cell Plating: Cells were collected, and 3 mL of cell culture medium (containing 10% FBS) was added to fully suspend the cells. Then, 100 μL cell suspension was added to a centrifuge tube, and 200 μL medium was added and mixed thoroughly for dilution. A Countess cell counting chamber slide was used to count the diluted cells in suspension using a microscope, and the cell density was adjusted to 1 ×10^6^ cells/mL. This cell suspension was mixed evenly to a final concentration of 100 ng/mL PMA and then transferred to six‐well plates (2 mL/well) or 12‐well plates (1 mL/well). After 48 h, the cells were induced into adherent macrophages for subsequent experiments.

Recombinant strains infection of macrophages. Protein expression was induced by inoculating recombinant strains into the 7H9 liquid medium, which contained 75 μg/mL Hygromycin B and 0.05% (w/v) Tween 80. The bacteria were collected, centrifuged and washed twice with 1 × PBS. Then, 10 mL RPMI 1640 medium was added for cell resuspension, and the mixture was centrifuged at 1000 rpm for 10 min. The upper bacterial suspension was retained. After turning on the spectrophotometer and selecting the RPMI 1640 medium for zero adjustment, the *OD*_600_ value of the recombinant strain suspension and the control strain suspension with empty vectors was adjusted to 0.4. The bacterial suspension was added to 12‐well plates already containing induced THP‐1 cells and was allowed to infect the THP‐1 cells for 4 h. Then, the media in the plate was removed and the cells were gently washed three times with 1 × PBS. After washing, the residual PBS was discarded, while RPMI 1640 complete culture medium containing 10% (v/v) FBS was prepared, and gentamicin (100 μg/mL) was added and mixed evenly. The prepared medium was added to the 12‐well plates, and the cells were continued to be cultured until the specified time point.

Intracellular survival experiment. After the cells were infected with the recombinant strains and control strains, the medium in the 12‐well plates was discarded at specified time points (6 h, 12 h, 24 h, 36 h, and 72 h) and washed with 1 × PBS three times. After washing, 1 mL of 0.025% (w/v) SDS solution was added to each well, and the cells were lysed for 10 min. Five dilution gradients were created using the 10‐fold dilution method, and 10 μL of the solution was titrated onto 7H9 solid plates for each gradient. After the plates were air‐dried, they were placed in a constant temperature incubator at 37°C for cell culture. The results were observed with a microscope and recorded after 3–4 d.

### Rv0801 protein structure and domain prediction

The functional domains of the protein‐coding region of the *Rv0801* gene were predicted by InterProScan (version 5.52_86.0) [86], and the protein structure was predicted using Alphafold2 (version 2.3.1) (https://github.com/lucidrains/alphafold2). In addition, the metal ion‐binding amino acid residues were predicted using MetalNet [87], and the protein structure was visualized by ChimeraX (version 1.7.1) [88].

### Structural variation analysis

INDELs Analysis. The methods for obtaining INDELs were described in the “SNPs and INDELs Calling” section. At first, we used custom Python scripts to calculate the density of INDELs (*d*) in each CDS and NCS across the whole genome for both MCCs and MCNCs. We eliminated INDELs found in over 95% of the MTBC strains that were gathered as they might be indicative of the traits of the reference genome H37Rv. Additionally, we excluded NCSs with length <200 bp. The calculation formula was as follows:

$$d=\frac{N_{\mathrm{INDELs}}}{N_{\mathrm{sample}}\cdot L_{\mathrm{seq}}},$$

where *N*_INDELs_ represents the total number of INDELs that occurred in the target CDS or NCS across all strains within the target clade, *N*_sample_ represents the number of strains in the target clade, and *L*_seq_ represents the length of the nucleotide sequence of the target CDS or NCS. Furthermore, we calculated the difference in INDELs density between MCC and the corresponding MCNC (*d*_MCC_ −* d*_MCNC_), and identified regions across the genome where this difference was significantly high. Next, for each locus on the whole genome where at least one strain had INDELs, we randomly sampled 100 strains from all the strains within the target lineage or clade, and calculated the proportion of strains with INDELs at that locus (*p*). This random sampling process was repeated 1000 times with different random seeds. Then the mean and standard deviation of *p* across all random processes were calculated. For the pairs of MCCs and MCNCs, we calculated the difference between the MCC and the MCNC (*p*_*MCC*_ −* p*_MCNC_) in the proportion of strains with INDELs at each locus on the whole genome, where at least one strain had INDELs, and identified loci where the *p*_MCC_ −* p*_MCNC_ value was high across the whole genome. For the key genes identified with a high *d*_MCC_ −* d*_MCNC_ value and possessing loci with high *p*_MCC_ −* p*_MCNC_ values, we annotated functional domains using the literature and InterProScan (version 5.52_86.0) [86]. The protein structure data were obtained from UniProtKB (https://www.uniprot.org), and visualized in ChimeraX (version 1.7.1) [88].

Large segment deletions (Regions of Difference, RDs) analysis. For the WGS data, quality control was performed using fastp (version 0.20.1) [70], followed by mapping of the sequencing reads to the reconstructed ancestral whole‐genome sequence of MTBC using bwa‐mem2 (version 2.2.1) [71]. The sequences were sorted and deduplicated using samtools (version 1.10) [73], and PCR duplicates were marked using sambamba (version 0.6.6) [89]. Since the second‐generation sequencing's short read lengths may raise the possibility of discovering false‐positive structural variations in repetitive genomic regions. Therefore, multiple methods were used to verify the true presence of RDs in the current study. The processed bam files were imported into Delly (version 0.7.6) [90], Manta (version 1.6.0) [91], and SvABA (version 1.1.0) [92], for deletion detection using parameters with minimum SV sizes of 50, 30, and 25 bp respectively, mapping quality thresholds of ≥ 20, and confidence intervals for breakpoint identification ranging from ±50 to ±300 bp depending on read support and local sequence complexity. To maximize reliability, these results were validated and merged by SURVIVOR (version 1.0.7) [93], using conservative parameters: a maximum distance threshold of 100 bp between breakpoints, requiring support from all the detection tools with adequate coverage depth (typically ≥ 20 supporting reads), enforcing strand consistency, and applying a stringent minimum SV length threshold of 200 bp. Only the RDs meeting these stringent criteria were retained. Using Bamdst (version 1.0.9) (https://github.com/shiquan/bamdst), regions of the genome not covered by sequencing were identified, and the RDs where the coverage overlapped with deletions detected by Bamdst at a rate of less than 75% were removed to ensure sufficient sequencing coverage for reliable variant calling. Furthermore, a custom Python script was used to merge duplicate RDs, filtering out those present in less than 10% of all samples in a single clade and those covering direct repeat sequences. The RDs unique to the four MCCs were then selected, and the genes associated with these RDs were identified. These genes' functional domains were annotated or predicted by literature and InterProScan (version 5.52_86.0) [86], and their functions were clarified through literature. Additionally, Alphafold2 (version 2.3.1) (https://github.com/lucidrains/alphafold2) was used to predict the protein structures of the involved genes both before and after deletion. The reliability of the predictions was assessed by both the predicted local distance difference test score and the predicted aligned error score in ChimeraX (version 1.7.1) [88]. The resulting protein structures were visualized using ChimeraX, which was also used to predict hydrophobic residues.

## AUTHOR CONTRIBUTIONS

**Wei Wu**: Study design; methodology; data collection; experimentation; data analysis; writing—original draft; writing—review and editing; visualization; funding acquisition. **Zhuochong Liu**: Data collection; data analysis. **Haiqi Chen**: Experimentation; data analysis; writing—original draft. **Yuhan Tang**: Data collection; data analysis. **Zhonghua Jiang**: Data collection. **Yiyang Zhang**: Data analysis. **Andong Zhang**: Writing—review and editing. **Zhiwei Zhou**: Writing—review and editing. **Robert S. Marks**: Writing—review and editing. **Fan Zhang**: Writing—review and editing. **Haibing Yuan**: Writing—review and editing; funding acquisition. **Yan Yu**: Writing—review and editing. **Kangshan Mao**: Writing—original draft; writing—review and editing. **András Dinnyés**: Writing—review and editing. **Nalin Rastogi**: Writing—original draft; writing—review and editing. **Jianping Xie**: Writing—review and editing; supervision. **Qun Sun**: Study design; writing—original draft; writing—review and editing; funding acquisition, supervision. All authors have read the final manuscript and approved it for publication.

## CONFLICT OF INTEREST STATEMENT

The authors declare no conflicts of interest.

## AI STATEMENT

A portion of the text refinement was performed by DeepSeek (R1, DeepSeek Inc.) and ChatGPT (o3, OpenAI), with the original drafts being processed using prompts dedicated solely to grammar standardization and sentence structure optimization. All the outputs underwent manual verification by authors to ensure factual consistency and eliminate potential AI‐induced bias. No AI tools were used in data generation, analysis, or visual content creation.

## ETHICS STATEMENT

No animals or humans were involved in this study.

## Supporting information

The online version contains supplementary figures and tables available.
**Figure S1:** Other indices representing the genetic diversity.
**Figure S2:** The MTBC maximum clade credibility trees.
**Figure S3:** Sampling locations of MTBC strains with geographic tags, whose whole‐genome sequencing data were used in this study.
**Figure S4:** The geographic divisions of China used in this study.
**Figure S5:** The geographic origins of the four sub‐lineages.
**Figure S6:** Mutation rate, dN/dS, and dR/dO historical changes of sub‐lineages and the MTBC clades predominantly found in non‐China regions (MCNCs).
**Figure S7:** Selection pressures on different gene groups of the MTBC clades predominantly found in China (MCCs) and the MTBC clades predominantly found in non‐China regions (MCNCs).
**Figure S8:** Verification of recombinant plasmid by PCR.
**Figure S9:** *p*_*MCC*_ *− p*_*MCNC*_ of other CDSs and NCSs with high value of *d*_*MCC*_ *− d*_*MCNC*_, and selection pressures on these genes shown in Figure 6.
**Figure S10:** The mean proportion of strains having INDELs at each base site in the genes, *PE_PGRS3* and *PE_PGRS4*, through random sampling process.
**Figure S11:** The mean proportion of strains having INDELs at each base site in the gene, *PE_PGRS17*, through random sampling process.
**Figure S12:** The mean proportion of strains having INDELs at each base site in the gene, *PE_PGRS28*, through random sampling process.

**Table S1:** Genes with high KL and MKL across the MTBC clades predominantly found in China.
**Table S2:** Genes with high KL and MKL across China's epidemic MTBC clades and their references.
**Table S3:** PCR amplification system for obtaining the sequence of *Rv0801*.
**Table S4:** The enzyme‐digest system of pALACE.
**Table S5:** The ligation system of *Rv0801* and pALACE.

## Data Availability

All the whole genome sequencing data have been deposited in GSA with the submission number CRA017764 (https://ngdc.cncb.ac.cn/gsa/browse/CRA017764). The whole genome sequencing data collected from public databases utilized in this study can be accessed from NCBI or GSA using the provided run accession numbers in the supplemental materials. Other raw data collected can also be obtained from the supplemental materials. The supplemental data are saved in GitHub https://github.com/WeiWuOpen/MTBCinChina-SupplementalData, and the scripts used are saved in GitHub https://github.com/WeiWuOpen/MTBCinChina. Supplementary materials (figures, tables, graphical abstract, slides, videos, Chinese translated version and update materials) may be found in the online DOI or iMeta Science http://www.imeta.science/imetaomics/.

## References

[1] Paulson, Tom . 2013. “Epidemiology: A Mortal Foe.” Nature 502: S2–S3. 10.1038/502S2a 24108078

[2] Houben, Rein M. G. J. , and Peter J. Dodd . 2016. “The Global Burden of Latent Tuberculosis Infection: A Re‐Estimation Using Mathematical Modelling.” PLoS Medicine 13: e1002152. 10.1371/journal.pmed.1002152 PMC5079585 27780211

[3] Goig, Galo A. , Etthel M. Windels , Chloé Loiseau , Christoph Stritt , Loza Biru , Sonia Borrell , Daniela Brites , and Sebastien Gagneux . 2025. “Ecology, Global Diversity and Evolutionary Mechanisms in the *Mycobacterium tuberculosis* Complex.” Nature Reviews Microbiology 23: 602–614. 10.1038/s41579-025-01159-w 40133503

[4] WHO . 2024. Global Tuberculosis Report 2024, Geneva: World Health Organization. https://www.who.int/teams/global-programme-on-tuberculosis-and-lung-health/tb-reports/global-tuberculosis-report-2024

[5] Sabin, Susanna , Alexander Herbig , Åshild J. Vågene , Torbjörn Ahlström , Gracijela Bozovic , Caroline Arcini , Denise Kühnert , and Kirsten I. Bos . 2020. “A Seventeenth‐Century *Mycobacterium tuberculosis* Genome Supports a Neolithic Emergence of the *Mycobacterium tuberculosis* Complex.” Genome Biology 21: 201. 10.1186/s13059-020-02112-1 PMC7418204 32778135

[6] O'Neill, Mary B. , Abigail Shockey , Alex Zarley , William Aylward , Vegard Eldholm , Andrew Kitchen , and Caitlin S. Pepperell . 2019. “Lineage Specific Histories of *Mycobacterium tuberculosis* Dispersal in Africa and Eurasia.” Molecular Ecology 28: 3241–3256. 10.1111/mec.15120 PMC6660993 31066139

[7] Liu, Qingyun , Aijing Ma , Lanhai Wei , Yu Pang , Beibei Wu , Tao Luo , Yang Zhou , et al. 2018. “China's Tuberculosis Epidemic Stems From Historical Expansion of Four Strains of *Mycobacterium tuberculosis* .” Nature Ecology & Evolution 2: 1982–1992. 10.1038/s41559-018-0680-6 PMC6295914 30397300

[8] Li, Mocen , Charlotte A. Roberts , Liang Chen , and Dongyue Zhao . 2019. “A Male Adult Skeleton From the Han Dynasty in Shaanxi, China (202 BC–220 AD) With Bone Changes That Possibly Represent Spinal Tuberculosis.” International Journal of Paleopathology 27: 9–16. 10.1016/j.ijpp.2019.08.003 31494353

[9] Fusegawa, Hisae , Bing‐Hua Wang , Kiyohiko Sakurai , Kazutoshi Nagasawa , Mitsuzane Okauchi , and Kouichi Nagakura . 2003. “Outbreak of Tuberculosis in a 2000‐year‐old Chinese Population.” Kansenshogaku Zasshi 77: 146–149. 10.11150/kansenshogakuzasshi1970.77.146 12708007

[10] Li, Yi‐Fan , Yang Yang , Xiang‐Long Kong , Wan‐Mei Song , Ya‐Meng Li , Ying‐Ying Li , Wei‐Wei Fang , et al. 2024. “Transmission Dynamics and Phylogeography of *Mycobacterium tuberculosis* in China Based on Whole‐Genome Phylogenetic Analysis.” International Journal of Infectious Diseases 140: 124–131. 10.1016/j.ijid.2023.10.015 37863309

[11] Stucki, David , Daniela Brites , Leïla Jeljeli , Mireia Coscolla , Qingyun Liu , Andrej Trauner , Lukas Fenner , et al. 2016. “ *Mycobacterium tuberculosis* Lineage 4 Comprises Globally Distributed and Geographically Restricted Sublineages.” Nature Genetics 48: 1535–1543. 10.1038/ng.3704 PMC5238942 27798628

[12] Ashton, Philip M. , Jaeyoon Cha , Catherine Anscombe , Nguyen T. T. Thuong , Guy E. Thwaites , and Timothy M. Walker . 2023. “Distribution and Origins of *Mycobacterium tuberculosis* L4 in Southeast Asia.” Microbial Genomics 9: 000955. 10.1099/mgen.0.000955 PMC9997747 36729036

[13] Shanmugam, Siva Kumar , Narender Kumar , Tamilzhalagan Sembulingam , Suresh Babu Ramalingam , Ashok Selvaraj , Udhayakumar Rajendhiran , Sudha Solaiyappan , et al. 2022. “ *Mycobacterium tuberculosis* Lineages Associated with Mutations and Drug Resistance in Isolates From India.” Microbiology Spectrum 10: e0159421. 10.1128/spectrum.01594-21 PMC9241780 35442078

[14] Casali, Nicola , Vladyslav Nikolayevskyy , Yanina Balabanova , Simon R. Harris , Olga Ignatyeva , Irina Kontsevaya , Jukka Corander , et al. 2014. “Evolution and Transmission of Drug‐Resistant Tuberculosis in a Russian Population.” Nature Genetics 46: 279–286. 10.1038/ng.2878 PMC3939361 24464101

[15] Brites, Daniela , and Sebastien Gagneux . 2015. “Co‐Evolution of *Mycobacterium tuberculosis* and *Homo sapiens* .” Immunological Reviews 264: 6–24. 10.1111/imr.12264 PMC4339235 25703549

[16] Barbier, Maxime , and Thierry Wirth . 2017. “The Evolutionary History, Demography, and Spread of the *Mycobacterium tuberculosis* Complex.” Tuberculosis and the Tubercle Bacillus 4: 453–473. 10.1128/9781555819569.ch20 27726798

[17] Coscolla, Mireia , Sebastien Gagneux , Fabrizio Menardo , Chloé Loiseau , Paula Ruiz‐Rodriguez , Sonia Borrell , Isaac Darko Otchere , et al. 2021. “Phylogenomics of Mycobacterium Africanum Reveals a New Lineage and a Complex Evolutionary History.” Microbial Genomics 7: 000477. 10.1099/mgen.0.000477 PMC8208692 33555243

[18] Wu, Beibei , Yue Wenlong Zhu , Qi Wang , Lin Wang , Zhengwei Zhou , Lijun Liu , Mathema Bi , et al. 2021. “Genetic Composition and Evolution of the Prevalent *Mycobacterium tuberculosis* Lineages 2 and 4 in the Chinese and Zhejiang Province Populations.” Cell & Bioscience 11: 162. 10.1186/s13578-021-00673-7 PMC8379736 34419157

[19] Luo, Tao , Iñaki Comas , Dan Luo , Bing Lu , Jie Wu , Lanhai Wei , Chongguang Yang , et al. 2015. “Southern East Asian Origin and Coexpansion of *Mycobacterium tuberculosis* Beijing Family With Han Chinese.” Proceedings of the National Academy of Sciences 112: 8136–8141. 10.1073/pnas.1424063112 PMC4491734 26080405

[20] Merker, Matthias , Camille Blin , Stefano Mona , Nicolas Duforet‐Frebourg , Sophie Lecher , Eve Willery , Michael G. B. Blum , et al. 2015. “Evolutionary History and Global Spread of the *Mycobacterium tuberculosis* Beijing Lineage.” Nature Genetics 47: 242–249. 10.1038/ng.3195 PMC11044984 25599400

[21] Reed, Michael B. , Victoria K. Pichler , Fiona McIntosh , Alicia Mattia , Ashley Fallow , Speranza Masala , Pilar Domenech , et al. 2009. “Major *Mycobacterium tuberculosis* Lineages Associate With Patient Country of Origin.” Journal of Clinical Microbiology 47: 1119–1128. 10.1128/JCM.02142-08 PMC2668307 19213699

[22] Luo, Yang , Chuan‐Chin Huang , Nicole C. Howard , Xin Wang , Qingyun Liu , Xinyi Li , Junhao Zhu , et al. 2024. “Paired Analysis of Host and Pathogen Genomes Identifies Determinants of Human Tuberculosis.” Nature Communications 15: 10393. 10.1038/s41467-024-54741-w PMC11607449 39613754

[23] Xia, Xuhua . 2023. “Horizontal Gene Transfer and Drug Resistance Involving *Mycobacterium tuberculosis* .” Antibiotics 12: 1367. 10.3390/antibiotics12091367 PMC10526031 37760664

[24] Allué‐Guardia, Anna , Juan I. García , and Jordi B. Torrelles . 2021. “Evolution of Drug‐Resistant *Mycobacterium tuberculosis* Strains and Their Adaptation to the Human Lung Environment.” Frontiers in Microbiology 12: 612675. 10.3389/fmicb.2021.612675 PMC7889510 33613483

[25] Liu, Qingyun , Haican Liu , Li Shi , Mingyu Gan , Xiuqin Zhao , Liang‐Dong Lyu , Howard E. Takiff , Kanglin Wan , and Qian Gao . 2021. “Local Adaptation of *Mycobacterium tuberculosis* on the Tibetan Plateau.” Proceedings of the National Academy of Sciences 118: e2017831118. 10.1073/pnas.2017831118 PMC8092575 33879609

[26] Chiang, Charleston W. K. , Serghei Mangul , Christopher Robles , and Sriram Sankararaman . 2018. “A Comprehensive Map of Genetic Variation in the World's Largest Ethnic Group—Han Chinese.” Molecular Biology and Evolution 35: 2736–2750. 10.1093/molbev/msy170 PMC6693441 30169787

[27] Arrieta‐Bolaños, Esteban , Diana Iraíz Hernández‐Zaragoza , and Rodrigo Barquera . 2023. “An HLA Map of the World: A Comparison of HLA Frequencies in 200 Worldwide Populations Reveals Diverse Patterns for Class I and Class II.” Frontiers in Genetics 14: 866407. 10.3389/fgene.2023.866407 PMC10076764 37035735

[28] Axelsson‐Robertson, Rebecca , André G. Loxton , Gerhard Walzl , Marthie M. Ehlers , Marleen M. Kock , Alimuddin Zumla , and Markus Maeurer . 2013. “A Broad Profile of Co‐Dominant Epitopes Shapes the Peripheral *Mycobacterium tuberculosis* Specific CD8+ T‐Cell Immune Response in South African Patients With Active Tuberculosis.” PLoS One 8: e58309. 10.1371/journal.pone.0058309 PMC3608651 23555576

[29] Gorna, Alina E. , Richard P. Bowater , and Jaroslaw Dziadek . 2010. “DNA Repair Systems and the Pathogenesis of *Mycobacterium tuberculosis*: Varying Activities at Different Stages of Infection.” Clinical Science 119: 187–202. 10.1042/CS20100041 20522025

[30] Behar, Samuel M. , and Volker Briken . 2019. “Apoptosis Inhibition by Intracellular Bacteria and Its Consequence on Host Immunity.” Current Opinion in Immunology 60: 103–110. 10.1016/j.coi.2019.05.007 PMC6800630 31228759

[31] Quigley, Jeff , V. Keith Hughitt , Carlos A. Velikovsky , Roy A. Mariuzza , Najib M. El‐Sayed , and Volker Briken . 2017. “The Cell Wall Lipid PDIM Contributes to Phagosomal Escape and Host Cell Exit of *Mycobacterium tuberculosis* .” mBio 8: e00148‐17. 10.1128/mbio.00148-17 PMC5340868 28270579

[32] Eisen, Rebecca J. , and Kenneth L. Gage . 2012. “Transmission of Flea‐Borne Zoonotic Agents.” Annual Review of Entomology 57: 61–82. 10.1146/annurev-ento-120710-100717 21888520

[33] DeJesus, Michael A. , Elias R. Gerrick , Weizhen Xu , Sae Woong Park , Jarukit E. Long , Cara C. Boutte , Eric J. Rubin , et al. 2017. “Comprehensive Essentiality Analysis of the *Mycobacterium tuberculosis* Genome via Saturating Transposon Mutagenesis.” mBio 8: e02133‐16. 10.1128/mBio.02133-16 PMC5241402 28096490

[34] Ma, Guoji , Lijing Liang , Yanhui Fan , Wenjuan Wang , Jiaqing Dai , and Zhifa Yuan . 2008. “Entropy Properties of Point Mutations [In Chinese].” Chinese Science Bulletin 53: 2318–2323. 10.1360/csb2008-53-19-2318

[35] He, Yujiao , Chunyan Zhou , Maolin Huang , Chunyan Tang , Xiao Liu , Yan Yue , Qingchun Diao , Zhebin Zheng , and Deming Liu . 2020. “Glyoxalase System: A Systematic Review of Its Biological Activity, Related‐Diseases, Screening Methods and Small Molecule Regulators.” Biomedicine & Pharmacotherapy 131: 110663. 10.1016/j.biopha.2020.110663 32858501

[36] Pepperell, Caitlin S. , Julie M. Granka , David C. Alexander , Marcel A. Behr , Linda Chui , Janet Gordon , Jennifer L. Guthrie , et al. 2011. “Dispersal of *Mycobacterium tuberculosis* via the Canadian fur trade.” Proceedings of the National Academy of Sciences 108: 6526–6531. 10.1073/pnas.1016708108 PMC3080970 21464295

[37] Pepperell, Caitlin S. , Amanda M. Casto , Andrew Kitchen , Julie M. Granka , Omar E. Cornejo , Eddie C. Holmes , Bruce Birren , James Galagan , and Marcus W. Feldman . 2013. “The Role of Selection in Shaping Diversity of Natural *M. tuberculosis* Populations.” PLoS Pathogens 9: e1003543. 10.1371/journal.ppat.1003543 PMC3744410 23966858

[38] Rahman, Shakibur , Sergei L. Kosakovsky Pond , Andrew Webb , and Jody Hey . 2021. “Weak Selection on Synonymous Codons Substantially Inflates dN/Ds Estimates in Bacteria.” Proceedings of the National Academy of Sciences 118: e2023575118. 10.1073/pnas.2023575118 PMC8157954 33972434

[39] Brynildsrud, Ola B. , Caitlin S. Pepperell , Philip Suffys , Louis Grandjean , Johana Monteserin , Nadia Debech , Jon Bohlin , et al. 2018. “Global Expansion of *Mycobacterium tuberculosis* Lineage 4 Shaped by Colonial Migration and Local Adaptation.” Science Advances 4: eaat5869. 10.1126/sciadv.aat5869 PMC6192687 30345355

[40] Altizer, Sonia , Richard S. Ostfeld , Pieter T. J. Johnson , Susan Kutz , and C. Drew Harvell . 2013. “Climate Change and Infectious Diseases: From Evidence to a Predictive Framework.” Science 341: 514–519. 10.1126/science.1239401 23908230

[41] Sharara, Sima L. , and Souha S. Kanj . 2014. “War and Infectious Diseases: Challenges of the Syrian Civil War.” PLoS Pathogens 10: e1004438. 10.1371/journal.ppat.1004438 PMC4231133 25393545

[42] Gröschel, Matthias I. , Francy J. Pérez‐Llanos , Roland Diel , Roger Vargas, Jr. , Vincent Escuyer , Kimberlee Musser , Lisa Trieu , et al. 2024. “Differential Rates of *Mycobacterium tuberculosis* Transmission Associate With Host–Pathogen Sympatry.” Nature Microbiology 9: 2113–2127. 10.1038/s41564-024-01758-y 39090390

[43] Zhu, Chendi , Tingting Yang , Jinfeng Yin , Hui Jiang , Howard E. Takiff , Qian Gao , Qingyun Liu , and Weimin Li . 2023. “The Global Success of *Mycobacterium tuberculosis* Modern Beijing Family Is Driven by a Few Recently Emerged Strains.” Microbiology Spectrum 11: e03339‐22. 10.1128/spectrum.03339-22 PMC10434187 37272796

[44] Yang, Z. H. , P. E. De Haas , D. Van Soolingen , J. D. Van Embden , and A. B. Andersen . 1994. “Restriction Fragment Length Polymorphism *Mycobacterium tuberculosis* Strains Isolated From Greenland During 1992: Evidence of Tuberculosis Transmission between Greenland and Denmark.” Journal of Clinical Microbiology 32: 3018–3025. 10.1128/jcm.32.12.3018-3025.1994 PMC264218 7883893

[45] Baker, Rachel E. , Ayesha S. Mahmud , Ian F. Miller , Malavika Rajeev , Fidisoa Rasambainarivo , Benjamin L. Rice , Saki Takahashi , et al. 2022. “Infectious Disease in an Era of Global Change.” Nature Reviews Microbiology 20: 193–205. 10.1038/s41579-021-00639-z PMC8513385 34646006

[46] Dopico, Xaquin Castro , Marina Evangelou , Ricardo C. Ferreira , Hui Guo , Marcin L. Pekalski , Deborah J. Smyth , Nicholas Cooper , et al. 2015. “Widespread Seasonal Gene Expression Reveals Annual Differences in Human Immunity and Physiology.” Nature Communications 6: 7000. 10.1038/ncomms8000 PMC4432600 25965853

[47] Comas, Iñaki , Jaidip Chakravartti , Peter M. Small , James Galagan , Stefan Niemann , Kristin Kremer , Joel D. Ernst , and Sebastien Gagneux . 2010. “Human T Cell Epitopes of *Mycobacterium tuberculosis* Are Evolutionarily Hyperconserved.” Nature Genetics 42: 498–503. 10.1038/ng.590 PMC2883744 20495566

[48] Stanley, Sydney , Xin Wang , Qingyun Liu , Young Yon Kwon , Abigail M. Frey , Nathan D. Hicks , Andrew J. Vickers , Sheng Hui , and Sarah M. Fortune . 2024. “Ongoing Evolution of the *Mycobacterium tuberculosis* Lactate Dehydrogenase Reveals the Pleiotropic Effects of Bacterial Adaption to Host Pressure.” PLoS Pathogens 20: e1012050. 10.1371/journal.ppat.1012050 PMC10931510 38422159

[49] Singh, Niti , Nishant Sharma , Padam Singh , Manitosh Pandey , Mohd Ilyas , Lovely Sisodiya , Tejaswini Choudhury , et al. 2022. “HupB, a Nucleoid‐Associated Protein, Is Critical for Survival of *Mycobacterium tuberculosis* under Host‐Mediated Stresses and for Enhanced Tolerance to Key First‐Line Antibiotics.” Frontiers in Microbiology 13: 937970. 10.3389/fmicb.2022.937970 PMC9441915 36071978

[50] Morgenstern, Jakob , Marta Campos Campos , Peter Nawroth , and Thomas Fleming . 2020. “The Glyoxalase System—New Insights into an Ancient Metabolism.” Antioxidants 9: 939. 10.3390/antiox9100939 PMC7600140 33019494

[51] Rachman, Helmy , Nayoung Kim , Timo Ulrichs , Sven Baumann , Lydia Pradl , Ali Nasser Eddine , Matthias Bild , et al. 2006. “Critical Role of Methylglyoxal and AGE in Mycobacteria‐Induced Macrophage Apoptosis and Activation.” PLoS One 1: e29. 10.1371/journal.pone.0000029 PMC1762319 17183656

[52] Anaya‐Sanchez, Andrea , Ying Feng , John C. Berude , and Daniel A. Portnoy . 2021. “Detoxification of Methylglyoxal by the Glyoxalase System Is Required for Glutathione Availability and Virulence Activation in Listeria Monocytogenes.” PLOS Pathogens 17: e1009819. 10.1371/journal.ppat.1009819 PMC8372916 34407151

[53] Delogu, Giovanni , Stewart T. Cole , and Roland Brosch . 2008. “The PE and PPE Protein Families of *Mycobacterium tuberculosis* .” In Handbook of Tuberculosis, 131–150, Weinheim, Germany: Wiley. 10.1002/9783527611614.ch7

[54] De Maio, Flavio , Rita Berisio , Riccardo Manganelli , and Giovanni Delogu . 2020. “PE_PGRS Proteins of *Mycobacterium tuberculosis*: A Specialized Molecular Task Force at the Forefront of Host–Pathogen Interaction.” Virulence 11: 898–915. 10.1080/21505594.2020.1785815 PMC7550000 32713249

[55] Orgeur, Mickael , and Roland Brosch . 2018. “Evolution of Virulence in the *Mycobacterium tuberculosis* Complex.” Current Opinion in Microbiology 41: 68–75. 10.1016/j.mib.2017.11.021 29216510

[56] Copin, Richard , Mireia Coscollá , Salome N. Seiffert , Graham Bothamley , Jayne Sutherland , Georgetta Mbayo , Sebastien Gagneux , and Joel D. Ernst . 2014. “Sequence Diversity in the Pe_Pgrs Genes of *Mycobacterium tuberculosis* Is Independent of Human T Cell Recognition.” mBio 5: e00960‐13. 10.1128/mbio.00960-13 PMC3903279 24425732

[57] De Maio, Flavio , Basem Battah , Valentina Palmieri , Linda Petrone , Francesco Corrente , Alessandro Salustri , Ivana Palucci , et al. 2018. “PE_PGRS3 of *Mycobacterium tuberculosis* Is Specifically Expressed at Low Phosphate Concentration, and Its Arginine‐Rich C‐Terminal Domain Mediates Adhesion and Persistence in Host Tissues When Expressed in *Mycobacterium smegmatis* .” Cellular Microbiology 20: e12952. 10.1111/cmi.12952 30192424

[58] Tian, Chen , and Xie Jian‐Ping . 2010. “Roles of PE_PGRS Family in *Mycobacterium tuberculosis* Pathogenesis and Novel Measures Against Tuberculosis.” Microbial Pathogenesis 49: 311–314. 10.1016/j.micpath.2010.07.004 20638467

[59] Qin, Lianhua , Jie Wang , Junmei Lu , Hua Yang , Ruijuan Zheng , Zhonghua Liu , Xiaochen Huang , et al. 2019. “A Deletion in the RD105 Region Confers Resistance to Multiple Drugs in *Mycobacterium tuberculosis* .” BMC Biology 17: 7. 10.1186/s12915-019-0628-6 PMC6347829 30683096

[60] Liu, Zhuochong , Zhonghua Jiang , Wei Wu , Xinyi Xu , Yudong Ma , Xiaomei Guo , Senlin Zhang , and Qun Sun . 2022. “Identification of Region of Difference and H37Rv‐Related Deletion in *Mycobacterium tuberculosis* Complex by Structural Variant Detection and Genome Assembly.” Frontiers in Microbiology 13: 984582. 10.3389/fmicb.2022.984582 PMC9493256 36160240

[61] Andreu, Núria , Carlos Y. Soto , Ignasi Roca , Carlos Martín , and Isidre Gibert . 2004. “ *Mycobacterium smegmatis* Displays the *Mycobacterium tuberculosis* Virulence‐Related Neutral Red Character When Expressing the Rv0577 Gene.” FEMS Microbiology Letters 231: 283–289. 10.1016/S0378-1097(04)00008-4 14987776

[62] Byun, Eui‐Hong , Woo Sik Kim , Jong‐Seok Kim , In Duk Jung , Yeong‐Min Park , Hwa‐Jung Kim , Sang‐Nae Cho , and Sung Jae Shin . 2012. “ *Mycobacterium tuberculosis* Rv0577, a Novel TLR2 Agonist, Induces Maturation of Dendritic Cells and Drives Th1 Immune Response.” The FASEB Journal 26: 2695–2711. 10.1096/fj.11-199588 22415304

[63] Sao Emani, C. , J. L. Gallant , I. J. Wiid , and B. Baker . 2019. “The Role of Low Molecular Weight Thiols in *Mycobacterium tuberculosis* .” Tuberculosis 116: 44–55. 10.1016/j.tube.2019.04.003 31153518

[64] Camassa, Serena , Ivana Palucci , Raffaella Iantomasi , Tiziana Cubeddu , Mariachiara Minerva , Flavio De Maio , Samuel Jouny , et al. 2017. “Impact of *pe_pgrs33* Gene Polymorphisms on *Mycobacterium tuberculosis* Infection and Pathogenesis.” Frontiers in Cellular and Infection Microbiology 7: 137. 10.3389/fcimb.2017.00137 PMC5399086 28484686

[65] Berisio, Rita , and Giovanni Delogu . 2022. “PGRS Domain Structures: Doomed to Sail the Mycomembrane.” PLoS Pathogens 18: e1010760. 10.1371/journal.ppat.1010760 PMC9436101 36048802

[66] Phelan, Jody E. , Denise M. O'sullivan , Diana Machado , Jorge Ramos , Yaa E. A. Oppong , Susana Campino , Justin O'grady , et al. 2019. “Integrating Informatics Tools and Portable Sequencing Technology for Rapid Detection of Resistance to Anti‐Tuberculous Drugs.” Genome Medicine 11: 41. 10.1186/s13073-019-0650-x PMC6591855 31234910

[67] Vilchèze, Catherine , Bo Yan , Rosalyn Casey , Suzie Hingley‐Wilson , Laurence Ettwiller , and William R. Jacobs, Jr. 2022. “Commonalities of *Mycobacterium tuberculosis* Transcriptomes in Response to Defined Persisting Macrophage Stresses.” Frontiers in Immunology 13: 909904. 10.3389/fimmu.2022.909904 PMC9283954 35844560

[68] Zhai, Weijie , Fengjuan Wu , Yiyuan Zhang , Yurong Fu , and Zhijun Liu . 2019. “The Immune Escape Mechanisms of *Mycobacterium tuberculosis* .” International Journal of Molecular Sciences 20: 340. 10.3390/ijms20020340 PMC6359177 30650615

[69] Scott, Lauren M. , and Mark V. Janikas . 2010. “Spatial Statistics in ArcGIS.” In Handbook of Applied Spatial Analysis: Software Tools, Methods and Applications (pp. 27–41). Berlin Heidelberg: Springer. 10.1007/978-3-642-03647-7_2

[70] Chen, Shifu , Yanqing Zhou , Yaru Chen , and Jia Gu . 2018. “fastp: An Ultra‐Fast All‐In‐One FASTQ Preprocessor.” Bioinformatics 34: i884–i890. 10.1093/bioinformatics/bty560 PMC6129281 30423086

[71] Vasimuddin, Md , Sanchit Misra , Heng Li , and Srinivas Aluru . 2019. “Efficient Architecture‐Aware Acceleration of BWA‐MEM for Multicore Systems.” *2019 IEEE International Parallel and Distributed Processing Symposium (IPDPS)*, 314–324. 10.1109/IPDPS.2019.00041

[72] DePristo, Mark A. , Eric Banks , Ryan Poplin , Kiran V. Garimella , Jared R. Maguire , Christopher Hartl , Anthony A. Philippakis , et al. 2011. “A Framework for Variation Discovery and Genotyping Using Next‐Generation DNA Sequencing Data.” Nature Genetics 43: 491–498. 10.1038/ng.806 PMC3083463 21478889

[73] Danecek, Petr , James K. Bonfield , Jennifer Liddle , John Marshall , Valeriu Ohan , Martin O. Pollard , Andrew Whitwham , et al. 2021. “Twelve Years of SAMtools and BCFtools.” GigaScience 10: giab008. 10.1093/gigascience/giab008 PMC7931819 33590861

[74] Nguyen, Lam‐Tung , Heiko A. Schmidt , Arndt von Haeseler , and Bui Quang Minh . 2014. “IQ‐TREE: A Fast and Effective Stochastic Algorithm for Estimating Maximum‐Likelihood Phylogenies.” Molecular Biology and Evolution 32: 268–274. 10.1093/molbev/msu300 PMC4271533 25371430

[75] Kalyaanamoorthy, Subha , Bui Quang Minh , Thomas K. F. Wong , Arndt von Haeseler , and Lars S. Jermiin . 2017. “ModelFinder: Fast Model Selection for Accurate Phylogenetic Estimates.” Nature Methods 14: 587–589. 10.1038/nmeth.4285 PMC5453245 28481363

[76] Letunic, Ivica , and Peer Bork . 2024. “Interactive Tree of Life (iTOL) v6: Recent Updates to the Phylogenetic Tree Display and Annotation Tool.” Nucleic Acids Research 52: W78–W82. 10.1093/nar/gkae268 PMC11223838 38613393

[77] Sagulenko, Pavel , Vadim Puller , and Richard A. Neher . 2018. “TreeTime: Maximum‐Likelihood Phylodynamic Analysis.” Virus Evolution 4: vex042. 10.1093/ve/vex042 PMC5758920 29340210

[78] Bouckaert, Remco , Joseph Heled , Denise Kühnert , Tim Vaughan , Chieh‐Hsi Wu , Dong Xie , Marc A. Suchard , Andrew Rambaut , and Alexei J. Drummond . 2014. “BEAST 2: A Software Platform for Bayesian Evolutionary Analysis.” PLoS Computational Biology 10: e1003537. 10.1371/journal.pcbi.1003537 PMC3985171 24722319

[79] Bielejec, Filip , Guy Baele , Bram Vrancken , Marc A. Suchard , Andrew Rambaut , and Philippe Lemey . 2016. “SpreaD3: Interactive Visualization of Spatiotemporal History and Trait Evolutionary Processes.” Molecular Biology and Evolution 33: 2167–2169. 10.1093/molbev/msw082 PMC6398721 27189542

[80] Shenhav, Liat , Mike Thompson , Tyler A. Joseph , Leah Briscoe , Ori Furman , David Bogumil , Itzhak Mizrahi , Itsik Pe'er , and Eran Halperin . 2019. “FEAST: Fast Expectation‐Maximization for Microbial Source Tracking.” Nature Methods 16: 627–632. 10.1038/s41592-019-0431-x PMC8535041 31182859

[81] Gu, Zuguang , Lei Gu , Roland Eils , Matthias Schlesner , and Benedikt Brors . 2014. “Circlize Implements and Enhances Circular Visualization in R.” Bioinformatics 30: 2811–2812. 10.1093/bioinformatics/btu393 24930139

[82] Liu, Qingyun , Junhao Zhu , Charles L. Dulberger , Sydney Stanley , Sean Wilson , Eun Seon Chung , Xin Wang , et al. 2022. “Tuberculosis Treatment Failure Associated With Evolution of Antibiotic Resilience.” Science 378: 1111–1118. 10.1126/science.abq2787 PMC9968493 36480634

[83] Trauner, Andrej , Qingyun Liu , Laura E. Via , Xin Liu , Xianglin Ruan , Lili Liang , Huimin Shi , et al. 2017. “The Within‐Host Population Dynamics of *Mycobacterium tuberculosis* Vary With Treatment Efficacy.” Genome Biology 18: 71. 10.1186/s13059-017-1196-0 PMC5395877 28424085

[84] Sherwin, William B . 2010. “Entropy and Information Approaches to Genetic Diversity and Its Expression: Genomic Geography.” Entropy 12: 1765–1798. 10.3390/e12071765

[85] Singh, Vinay K. , A. K. Mangalam , S. Dwivedi , and S. Naik . 1998. “Primer Premier: Program for Design of Degenerate Primers From a Protein Sequence.” BioTechniques 24: 318–319. 10.2144/98242pf02 9494736

[86] Jones, Philip , David Binns , Hsin‐Yu Chang , Matthew Fraser , Weizhong Li , Craig McAnulla , Hamish McWilliam , et al. 2014. “InterProScan 5: Genome‐Scale Protein Function Classification.” Bioinformatics 30: 1236–1240. 10.1093/bioinformatics/btu031 PMC3998142 24451626

[87] Cheng, Yao , Haobo Wang , Hua Xu , Yuan Liu , Bin Ma , Xuemin Chen , Xin Zeng , et al. 2023. “Co‐Evolution‐Based Prediction of Metal‐Binding Sites in Proteomes by Machine Learning.” Nature Chemical Biology 19: 548–555. 10.1038/s41589-022-01223-z 36593274

[88] Meng, Elaine C. , Thomas D. Goddard , Eric F. Pettersen , Greg S. Couch , Zach J. Pearson , John H. Morris , and Thomas E. Ferrin . 2023. “UCSF ChimeraX: Tools for Structure Building and Analysis.” Protein Science 32: e4792. 10.1002/pro.4792 PMC10588335 37774136

[89] Tarasov, Artem , Albert J. Vilella , Edwin Cuppen , Isaac J. Nijman , and Pjotr Prins . 2015. “Sambamba: Fast Processing of NGS Alignment Formats.” Bioinformatics 31: 2032–2034. 10.1093/bioinformatics/btv098 PMC4765878 25697820

[90] Rausch, Tobias , Thomas Zichner , Andreas Schlattl , Adrian M. Stütz , Vladimir Benes , and Jan O. Korbel . 2012. “DELLY: Structural Variant Discovery by Integrated Paired‐End and Split‐Read Analysis.” Bioinformatics 28: i333–i339. 10.1093/bioinformatics/bts378 PMC3436805 22962449

[91] Chen, Xiaoyu , Ole Schulz‐Trieglaff , Richard Shaw , Bret Barnes , Felix Schlesinger , Morten Källberg , Anthony J. Cox , Semyon Kruglyak , and Christopher T. Saunders . 2016. “Manta: Rapid Detection of Structural Variants and Indels for Germline and Cancer Sequencing Applications.” Bioinformatics 32: 1220–1222. 10.1093/bioinformatics/btv710 26647377

[92] Wala, Jeremiah A. , Pratiti Bandopadhayay , Noah F. Greenwald , Ryan O'Rourke , Ted Sharpe , Chip Stewart , Steve Schumacher , et al. 2018. “SvABA: Genome‐Wide Detection of Structural Variants and Indels by Local Assembly.” Genome Research 28: 581–591. 10.1101/gr.221028.117 PMC5880247 29535149

[93] Jeffares, Daniel C. , Clemency Jolly , Mimoza Hoti , Doug Speed , Liam Shaw , Charalampos Rallis , Francois Balloux , et al. 2017. “Transient Structural Variations Have Strong Effects on Quantitative Traits and Reproductive Isolation in Fission Yeast.” Nature Communications 8: 14061. 10.1038/ncomms14061 PMC5286201 28117401

