# Supplementary material for: Cross‐continental transmission and host adaptation of Mycobacterium tuberculosis in China unveiled by population history reconstruction and adaptive evolution signal detection
Source: IMetaOmics. 2025 Sep 20;2(4):e70052. doi: 10.1002/imo2.70052 (PMC12806118; doi:10.1002/imo2.70052)
Supplement: Supplementary file 1 — The online version contains supplementary figures and tables available. Figure S1: Other indices representing the genetic diversity. Figure S2: The MTBC maximum clade credibility trees. Figure S3: Sampling locations of MTBC strains with geographic tags, whose whole‐genome sequencing data were used in this study. Figure S4: The geographic divisions of China used in this study. Figure S5: The geographic origins of the four sub‐lineages. Figure S6: Mutation rate, dN/dS, and dR/dO historical changes of sub‐lineages and the MTBC clades predominantly found in non‐China regions (MCNCs). Figure S7: Selection pressures on different gene groups of the MTBC clades predominantly found in China (MCCs) and the MTBC clades predominantly found in non‐China regions (MCNCs). Figure S8: Verification of recombinant plasmid by PCR. Figure S9: p MCC − p MCNC of other CDSs and NCSs with high value of d MCC − d MCNC , and selection pressures on these genes shown in Figure 6. Figure S10: The mean proportion of strains having INDELs at each base site in the genes, PE_PGRS3 and PE_PGRS4, through random sampling process. Figure S11: The mean proportion of strains having INDELs at each base site in the gene, PE_PGRS17, through random sampling process. Figure S12: The mean proportion of strains having INDELs at each base site in the gene, PE_PGRS28, through random sampling process. [file IMO2-2-e70052-s001.docx]

Supporting information to

**Cross-Continental Transmission and Host Adaptation of *Mycobacterium tuberculosis* in China Unveiled by Population History Reconstruction and Adaptive Evolution Signal Detection**

**Running title:** MTBC Cross-Continental Transmission and Host Adaptation in China

Wei Wu^1^, Zhuochong Liu^1^, Haiqi Chen^2^, Yuhan Tang^1^, Zhonghua Jiang^3^, Yiyang Zhang^1^, Andong Zhang^1^, Zhiwei Zhou^1^, Robert S. Marks^4^, Fan Zhang^5^, Haibing Yuan^5^, Yan Yu^1^, Kangshan Mao^1^, András Dinnyés^1,6,7^, Nalin Rastogi^8^, Jianping Xie^2^*****, Qun Sun^1^*****

^1^ Key Laboratory of Bio-resources and Eco-environment of the Ministry of Education, College of Life Sciences, Sichuan University, Chengdu 610064, China.

^2^ Institute of Modern Biopharmaceuticals, School of Life Sciences, Southwest University, Chongqing 400715, China.

^3^ Respiratory and Critical Care Medicine, Molecularly Targeted Research and Development Laboratory, West China Hospital, Sichuan University, Chengdu 610041, China.

^4^ Department for Biotechnology Engineering, Ben Gurion University of the Negev, Be'er Sheva 84105, Israel.

^5^ Center for Archaeological Science, Sichuan University, Chengdu 610064, China.

^6^ BioTalentum Ltd., Aulich Lajos Str. 26, 2100 Gödöllő, Hungary.

^7^ Department of Physiology and Animal Health, Institute of Physiology and Animal Nutrition, Hungarian University of Agriculture and Life Sciences, H-2100, Gödöllő, Hungary.

^8^ WHO Supranational TB Reference Laboratory, Institut Pasteur de Guadeloupe, Abymes 97139, Guadeloupe, France.

* Correspondence: georgex@swu.edu.cn (Jianping Xie), qunsun@scu.edu.cn (Qun Sun).

The Supplementary Information file includes:

(1) Figure S1 to S12;

(2) Titles for Table S1 to S5;

(3) Legends for Data S1 to S8;

(4) Supplemental References.

Other Supplemental Materials for this manuscript include the following:

(1) Table S1 to S5;

(2) Data S1 to S8.


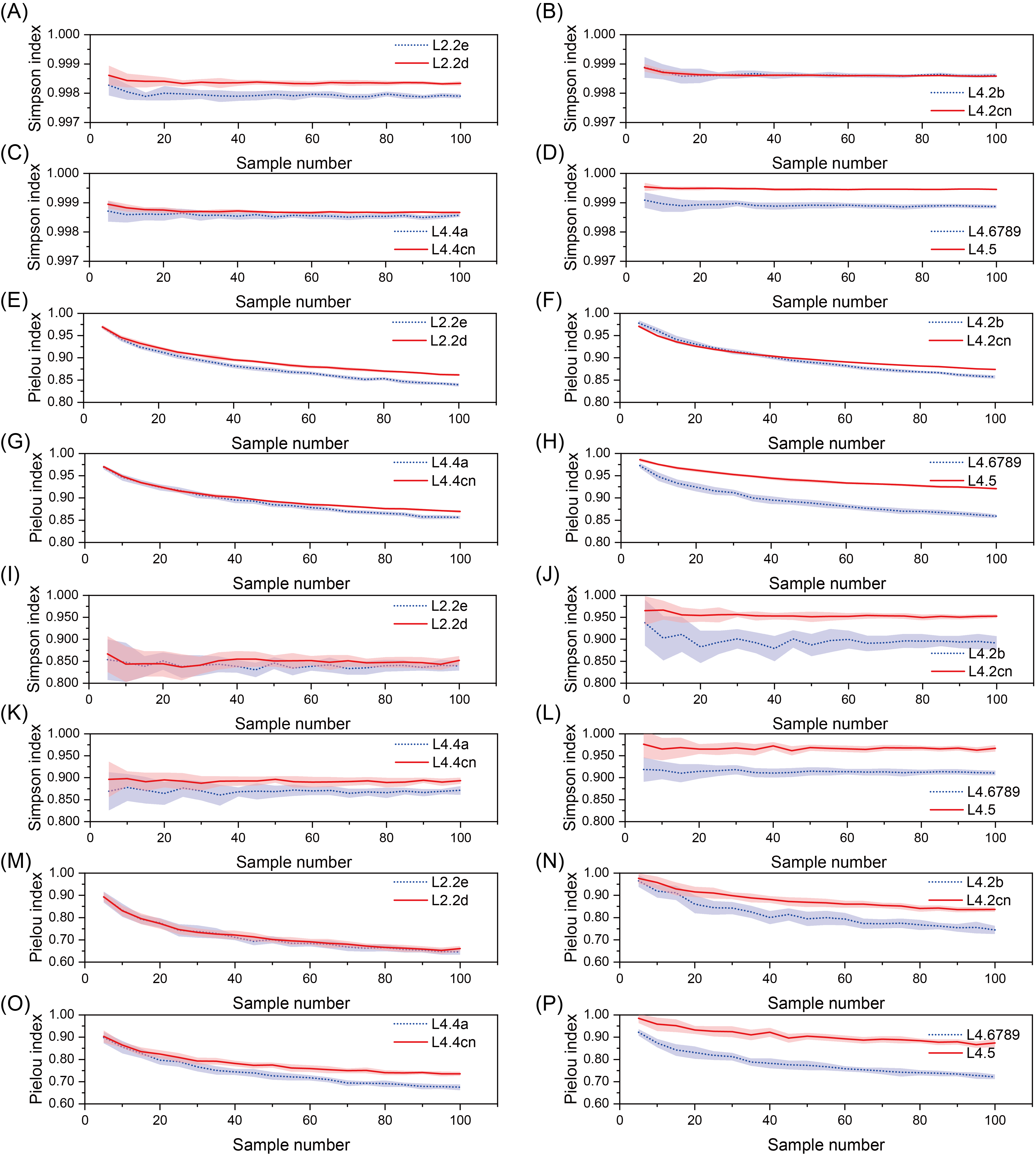


Figure S1. Other indices representing the genetic diversity. (A−H) Curve of Simpson index (A−D) and pielou index (E−H) with increasing randomly sampled strains number of four pairs of the MTBC clades predominantly found in China (MCCs) and the MTBC clades predominantly found in non-China regions (MCNCs) for all genes of the whole genome. (I−P) Curve of Simpson index (I−L) and pielou index (M−P) with increasing randomly sampled strains number of four pairs of MCCs and MCNCs for T cell epitopes.


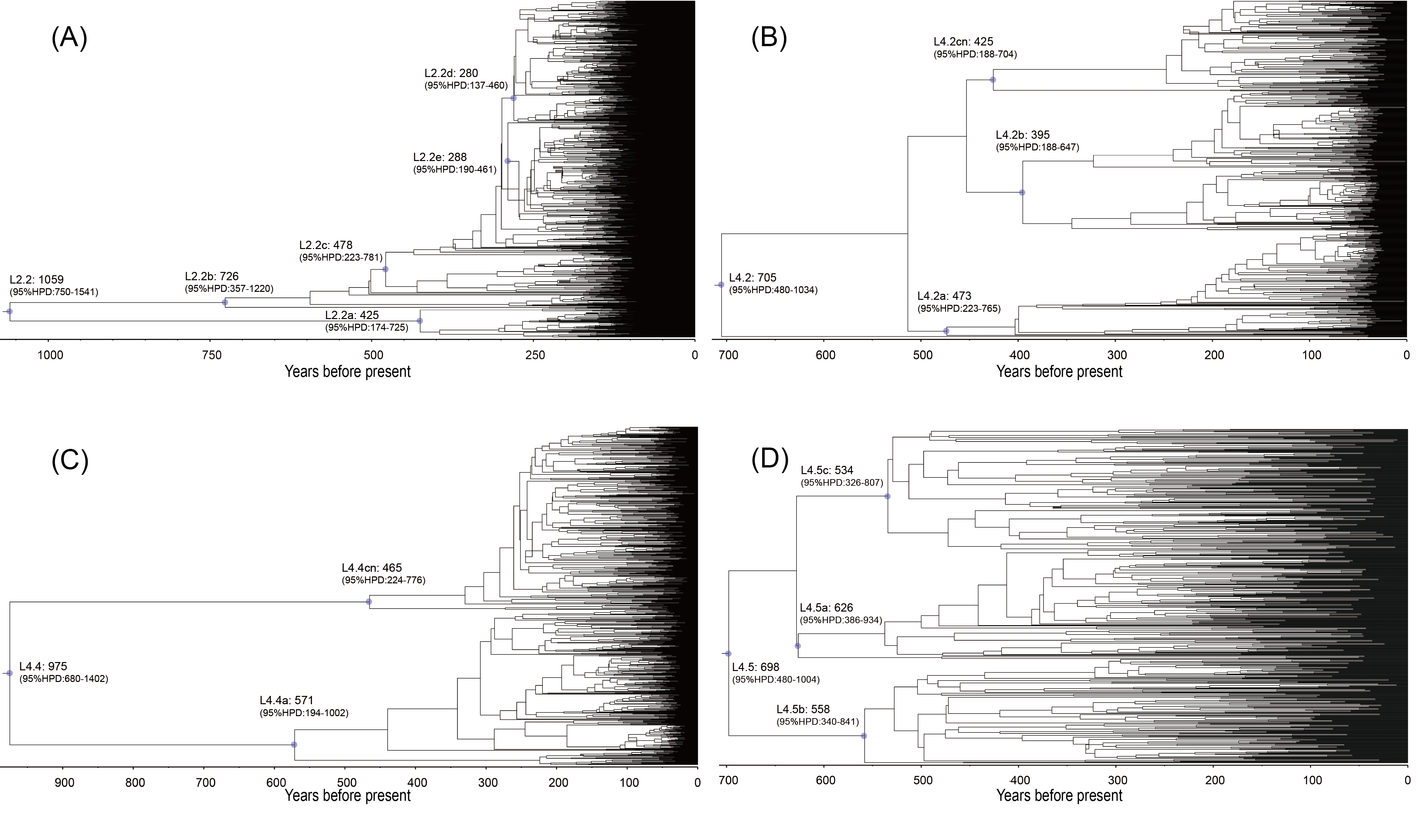


Figure S2. The MTBC maximum clade credibility trees. (A−D) The trees of L2.2 (A), L4.2 (B), L4.4(C), and L4.5(D). The heights of these trees were generated by BEAST2. The mean and 95% HPD intervals of the heights of nodes ancestral to root and important clades are indicated. The time scale is expressed as “years before present”, with the most recent time as A.D. 2020.


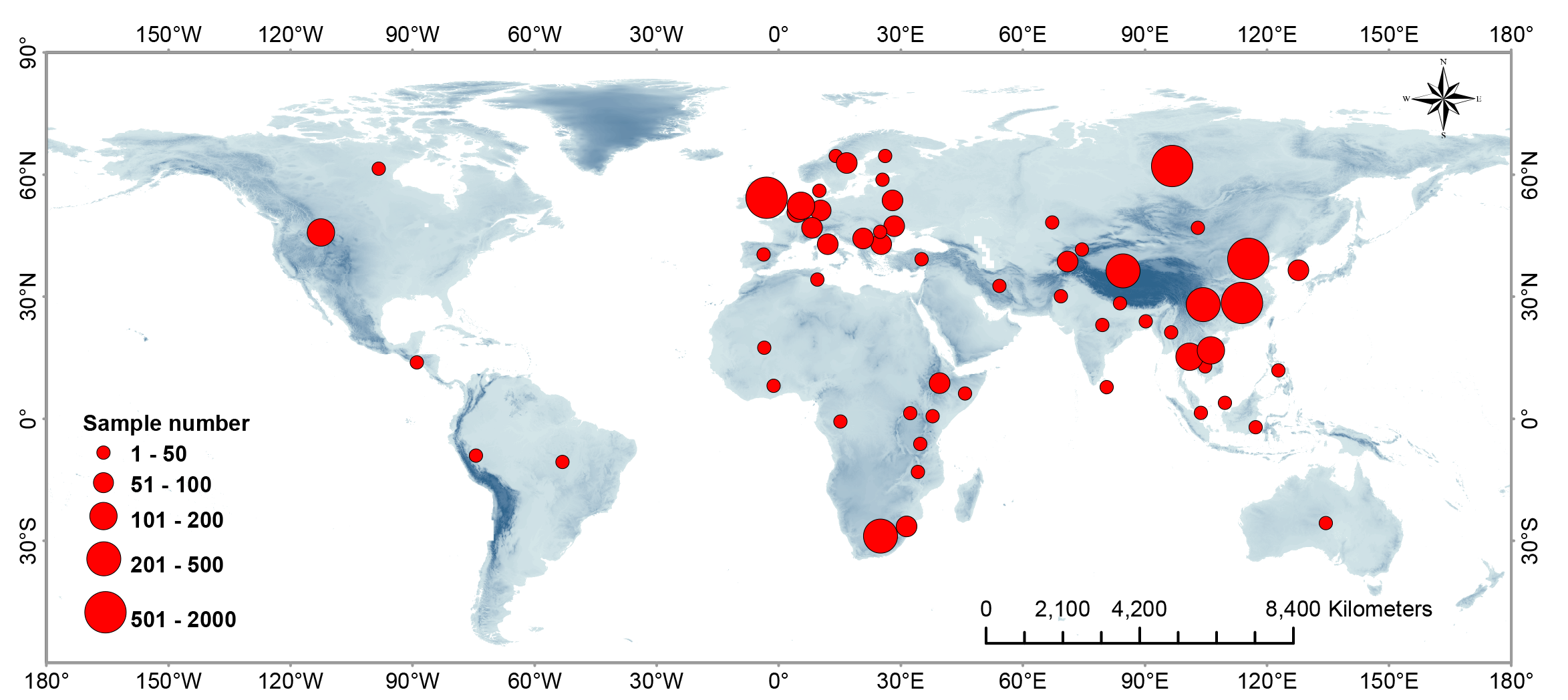


Figure S3. Sampling locations of MTBC strains with geographic tags, whose whole-genome sequencing data were used in this study.


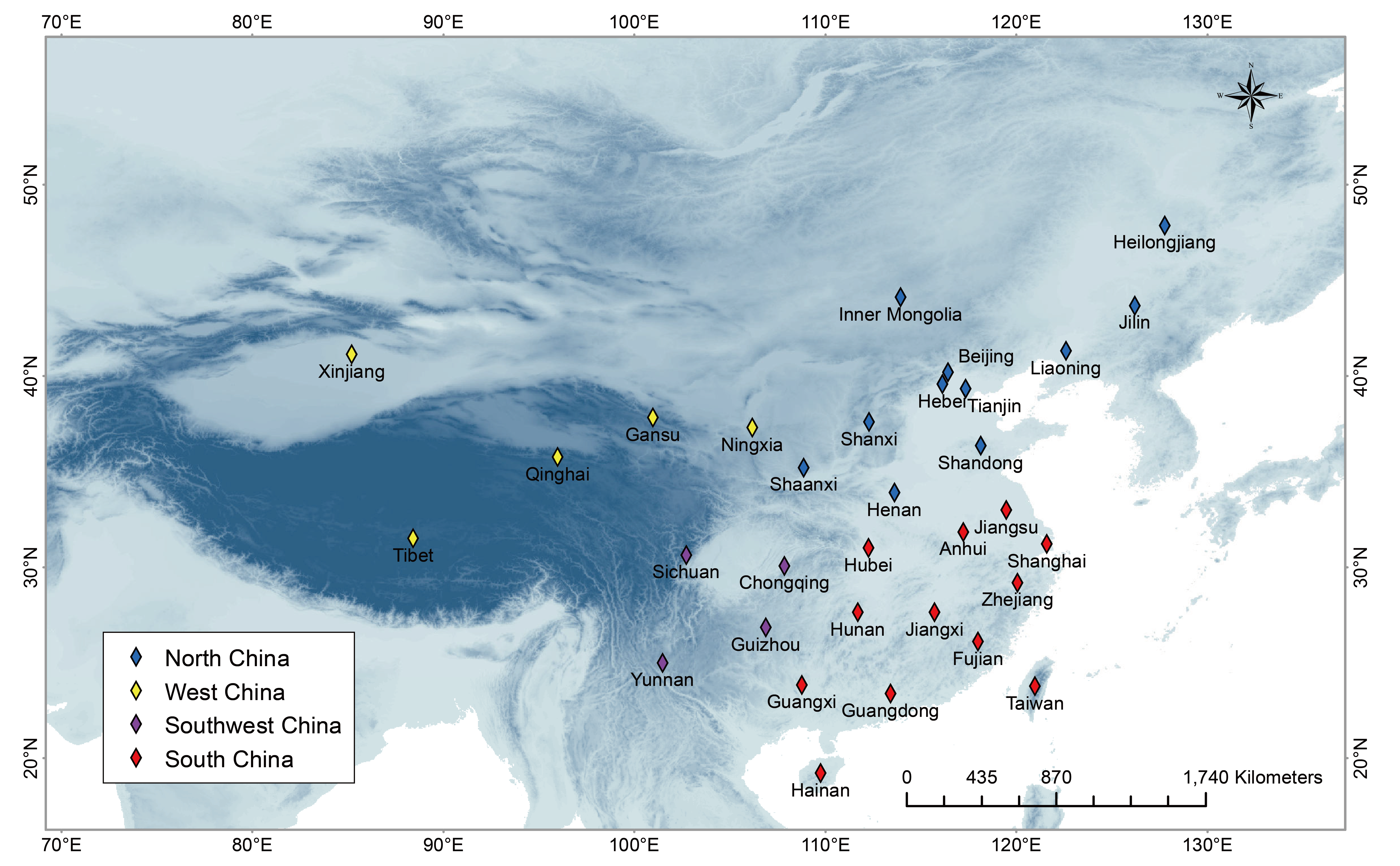


Figure S4. The geographic divisions of China used in this study. All provinces, except Hong Kong and Macao, of China are classified into four regions, as North China (blue), West China (yellow), South China (red), and Southwest China (purple).


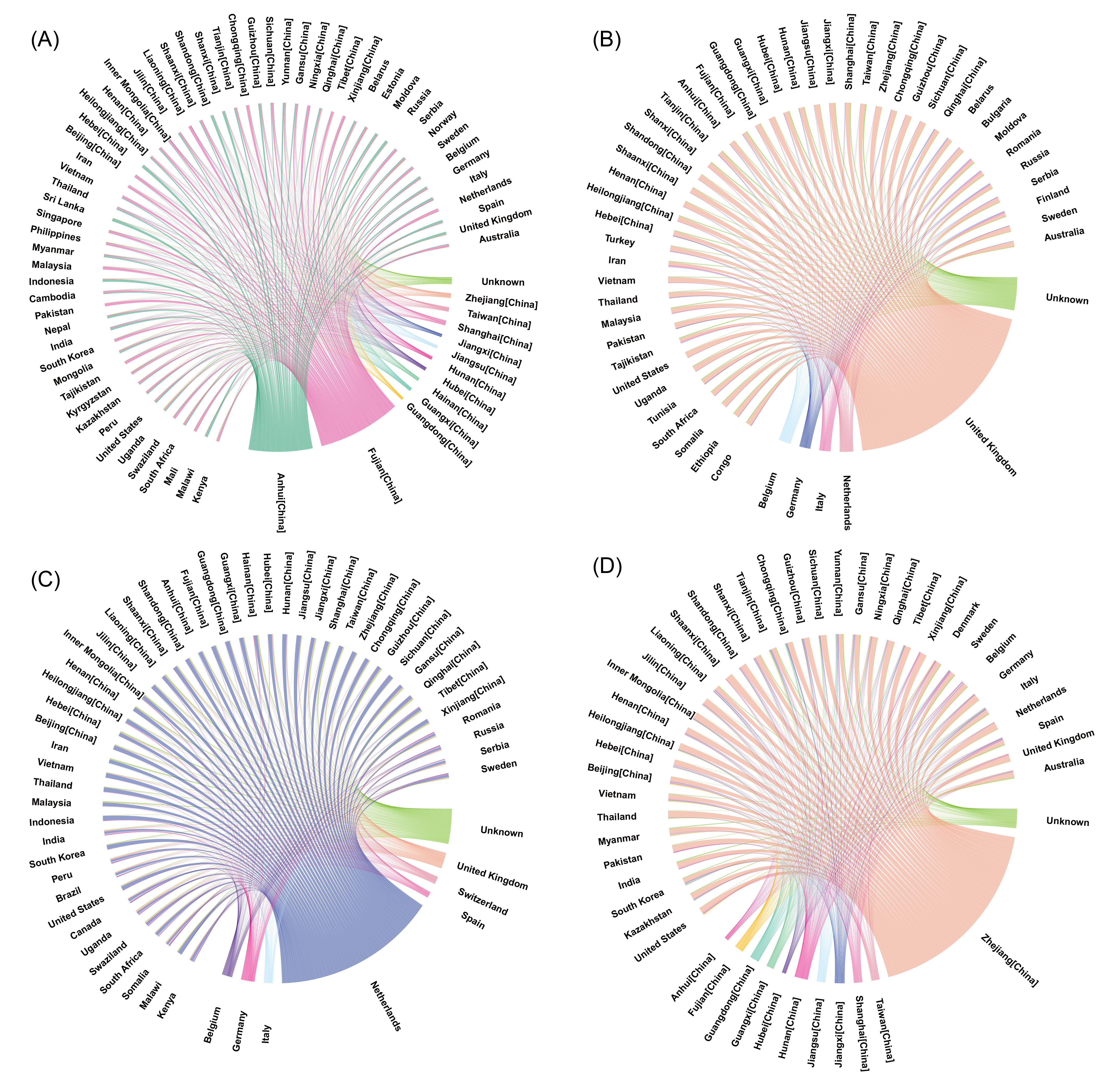


Figure S5. The geographic origins of the four sub−lineages. (A−D) Estimated geographic origin composition of L2.2 (A), L4.2 (B), L4.4 (C), and L4.5 (D), based on their whole-genome SNPs characteristics through the source-tracking algorithm. In each diagram, regions marked with “[China]” indicate provinces of China, and the regions in lower right are “source regions”, and those in the upper left section are “receiving regions”.


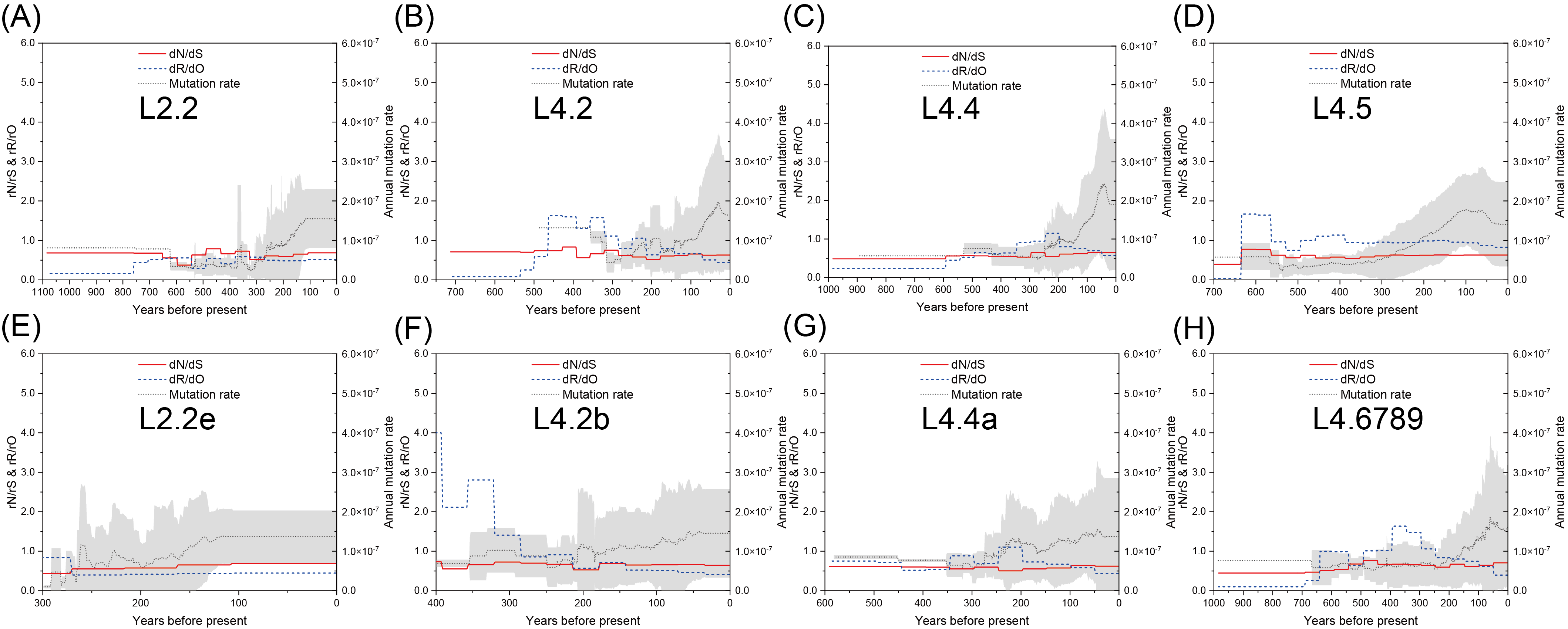


Figure S6. Mutation rate, dN/dS, and dR/dO historical changes of sub-lineages and the MTBC clades predominantly found in non-China regions (MCNCs). (A−D) Mutation rate, dN/dS, and dR/dO historical changes of the four sub-lineages for all genes on the whole genome. (E−H) Mutation rate, dN/dS, and dR/dO historical changes of the four MCNCs for all genes on the whole genome. The time scale is expressed as “years before present”, with the most recent time as A.D. 2020.


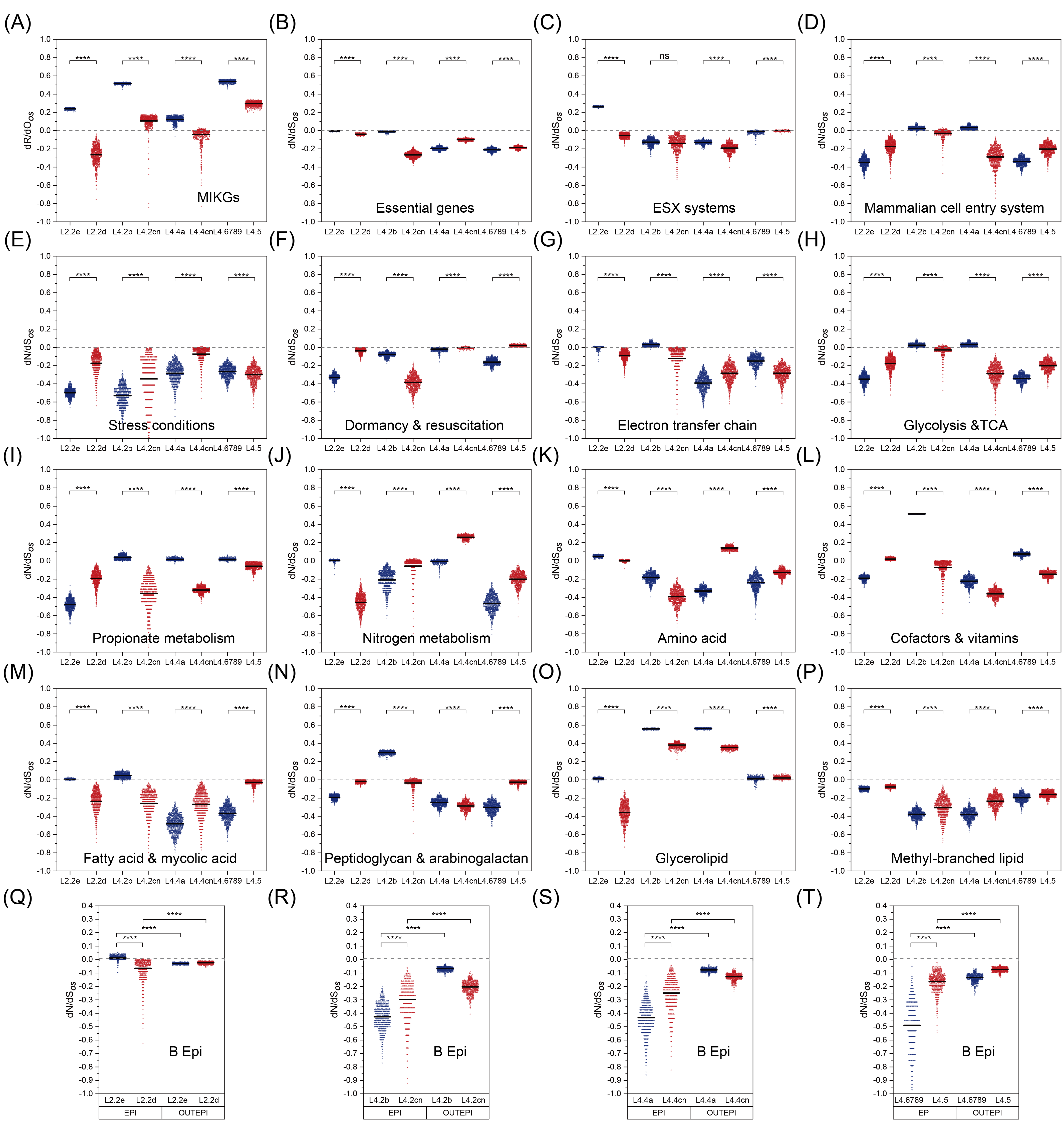


Figure S7. Selection pressures on different gene groups of the MTBC clades predominantly found in China (MCCs) and the MTBC clades predominantly found in non-China regions (MCNCs). (A) dR/dO*_OS_* of the macrophage infection key genes (MIKGs) for the four MCCs and corresponding MCNCs. (B−P) dN/dS*_OS_* of essential genes (B), genes associated with virulence factors (C, D), stress conditions (E), dormancy and resuscitation regulation (F), energy metabolism (G), central carbon metabolism (H, I), nitrogen metabolism (J), amino acid biosynthesis (K), cofactors and vitamins metabolism (L) and cell envelopes formation (M−P) for the four MCCs and corresponding MCNCs. (Q−T) dN/dS*_OS_* of B cell epitopes (B Epi) for the four MCCs and corresponding MCNCs. NEpi (non-epitope) represents the sequences other than Epi within coding sequences that include Epi. The *p* values are given by the two-sided Mann-Whitney *U* test, **** presents *p* < 0.0001, ns presents *p* > 0.05.


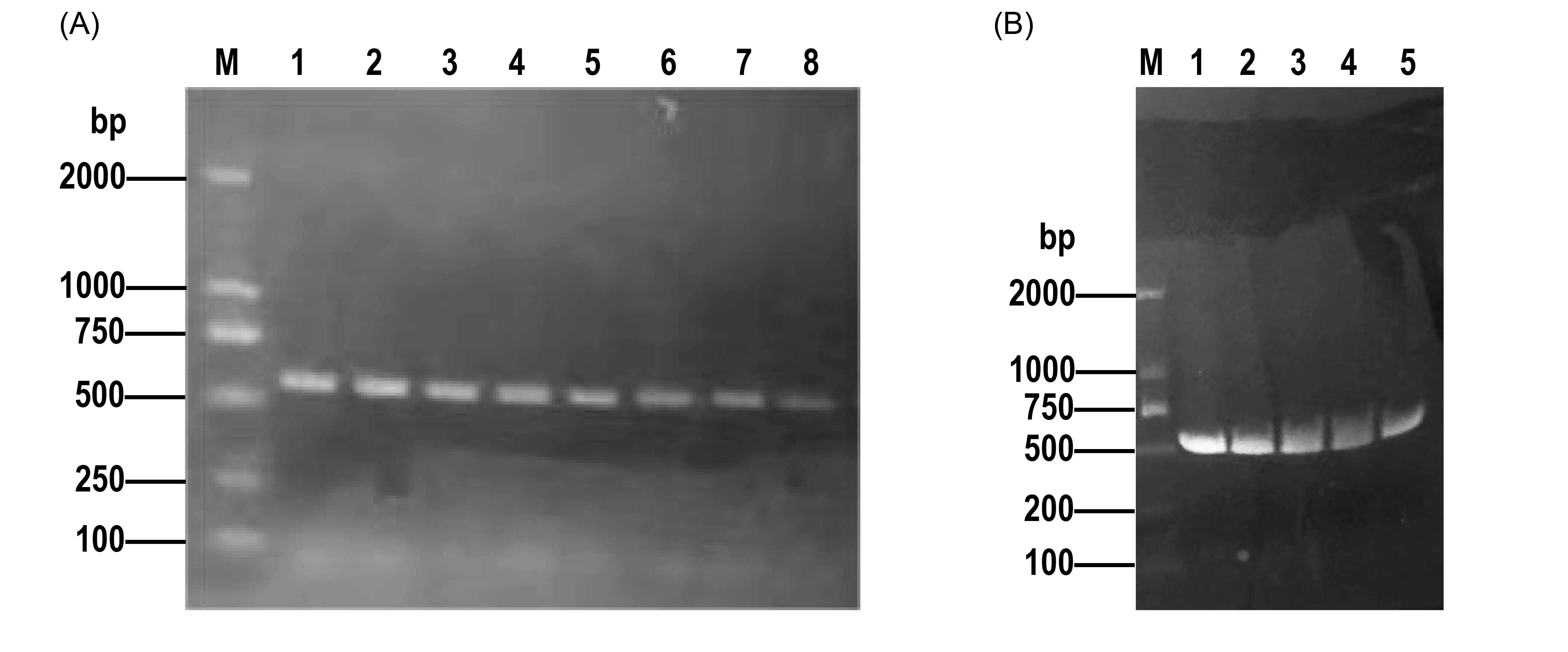


Figure S8. Verification of recombinant plasmid by PCR. (A) *Rv0801* was ligated into the pALACE plasmid and successfully expressed in Escherichia coli DH5α which was verified by bacterial liquid PCR with lanes 1-8 of recombinant strains and lane M of DL 2000 marker. (B) The recombinant plasmid was successfully transferred into *Mycobacterium smegmatis*.


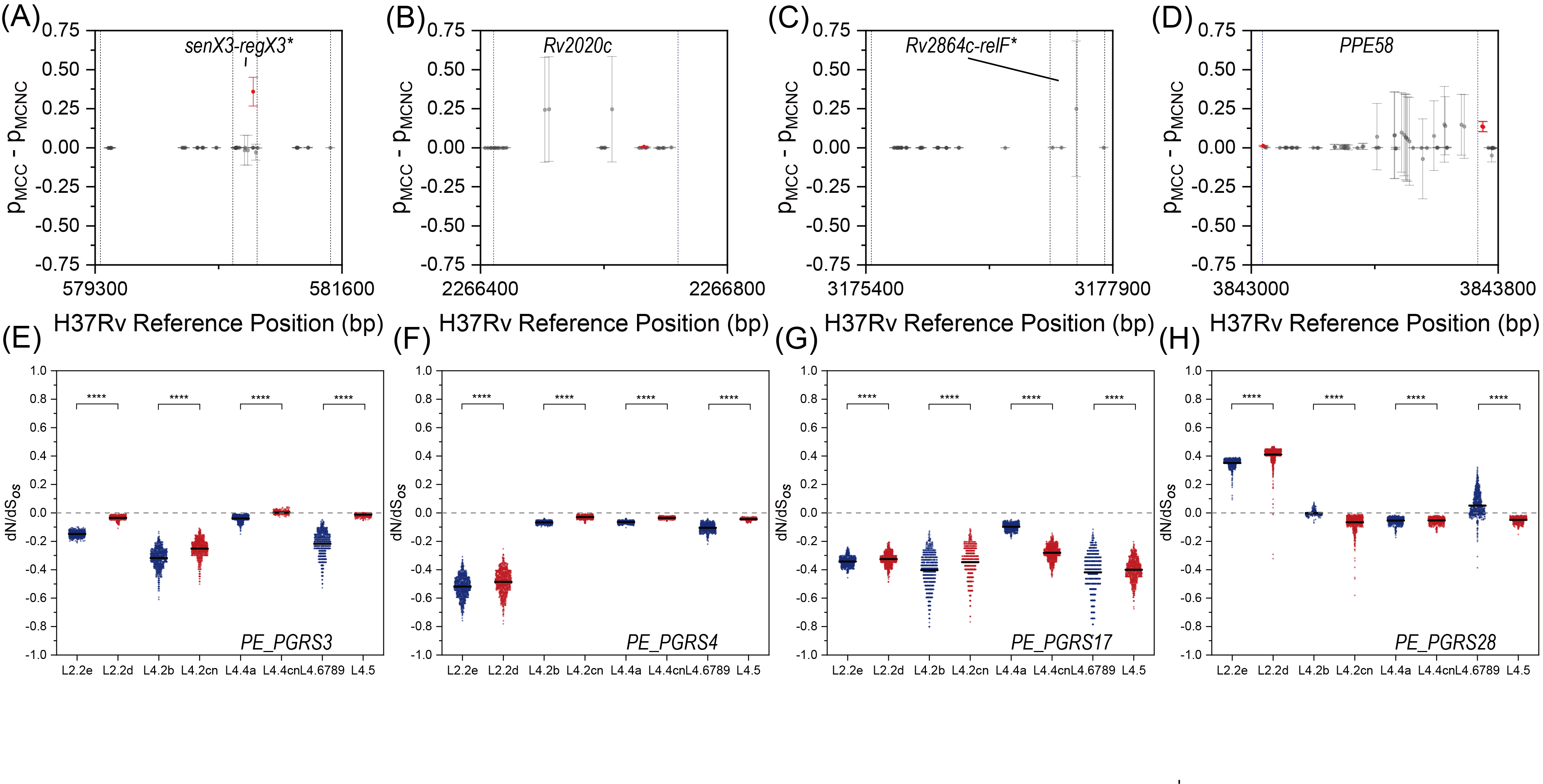


Figure S9. *p_MCC_ - p_MCNC_* of other CDSs and NCSs with high value of *d_MCC_ - d_MCNC_*, and selection pressures on these genes shown in Figure 6. (A−D) *p_MCC_ - p_MCNC_* at each base site in the NCS between *senX3* and *regX3* (A), *p_MCC_ - p_MCNC_* at each base site in the CDS of *Rv2020c* (B), *p_MCC_ - p_MCNC_* at each base site in the NCS between *Rv2864c* and *relF* (C), *p_MCC_ - p_MCNC_* at each base site in the CDS of *PPE58* (D). Red points represent base sites where > 0 for all four pairs of MCCs and MCNCs. The range of each CDS or NCS represents between the dotted lines. (E−H) dN/dS*_OS_* of the four MCCs and corresponding MCNCs for *PE_PGRS3* (E), *PE_PGRS4* (F), *PE_PGRS17* (G) and *PE_PGRS28* (H). CDS is short for coding sequence, NCS for non-coding sequence, MCC for the MTBC clade predominantly found in China, MCNC for the MTBC clade predominantly found in China. The *p* values are given by the two-sided Mann-Whitney *U* test, and **** presents *p* < 0.0001.


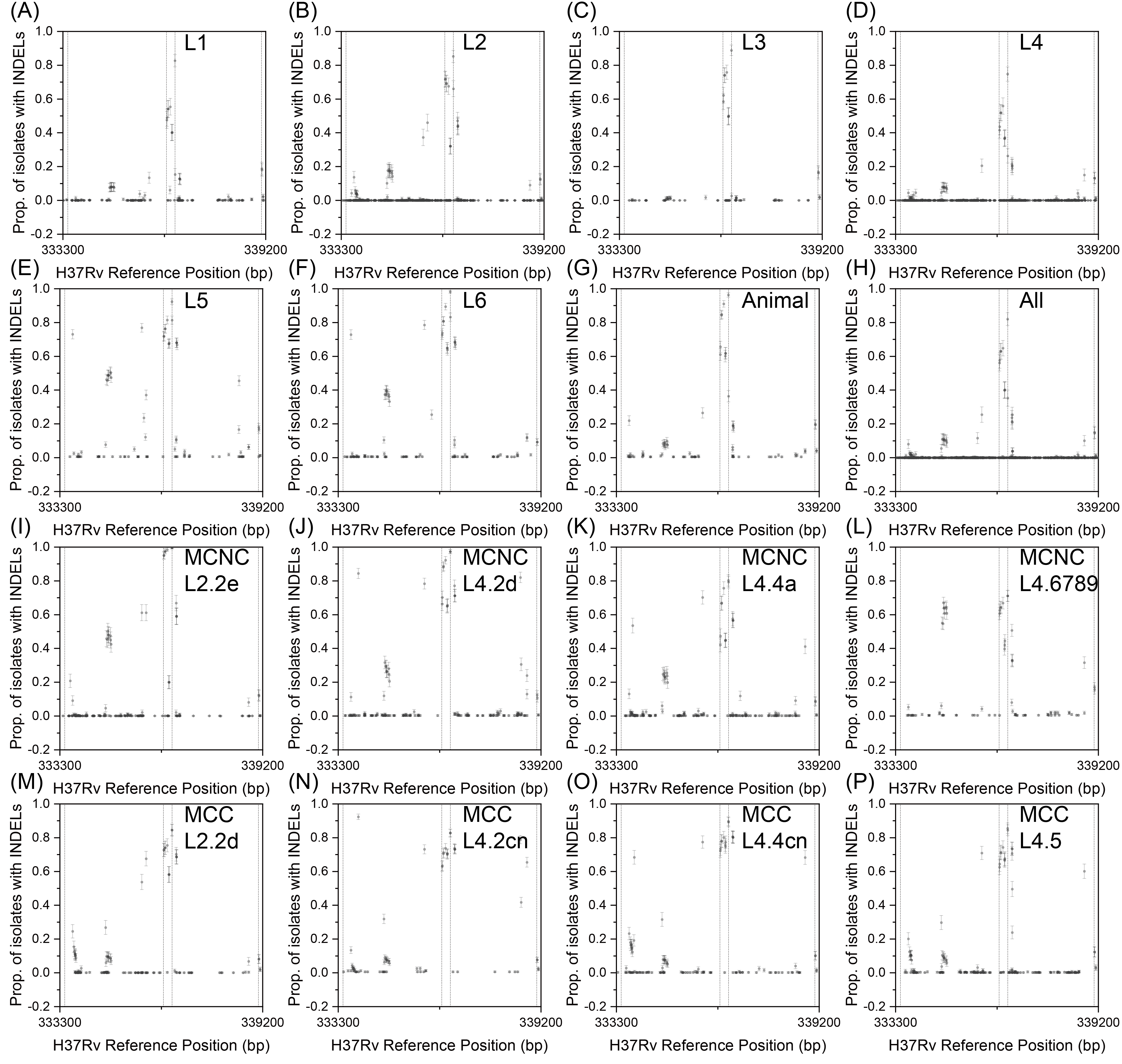


Figure S10. The mean proportion of strains having INDELs at each base site in the genes, *PE_PGRS3* and *PE_PGRS4*, through random sampling process. (A−F) Randomly sampled strains come from lineages L1-6. (G) Strains from animal lineages. (H) Strains from all 23,873 MTBC strains. (I−L) Strains from four MCNCs (the MTBC clades predominantly found in non-China regions). (M−P) Strains from four MCCs (the MTBC clades predominantly found in China). The range of genes represents between the dotted lines, where *PE_PGRS3* on the left side and *PE_PGRS4* on the right side.


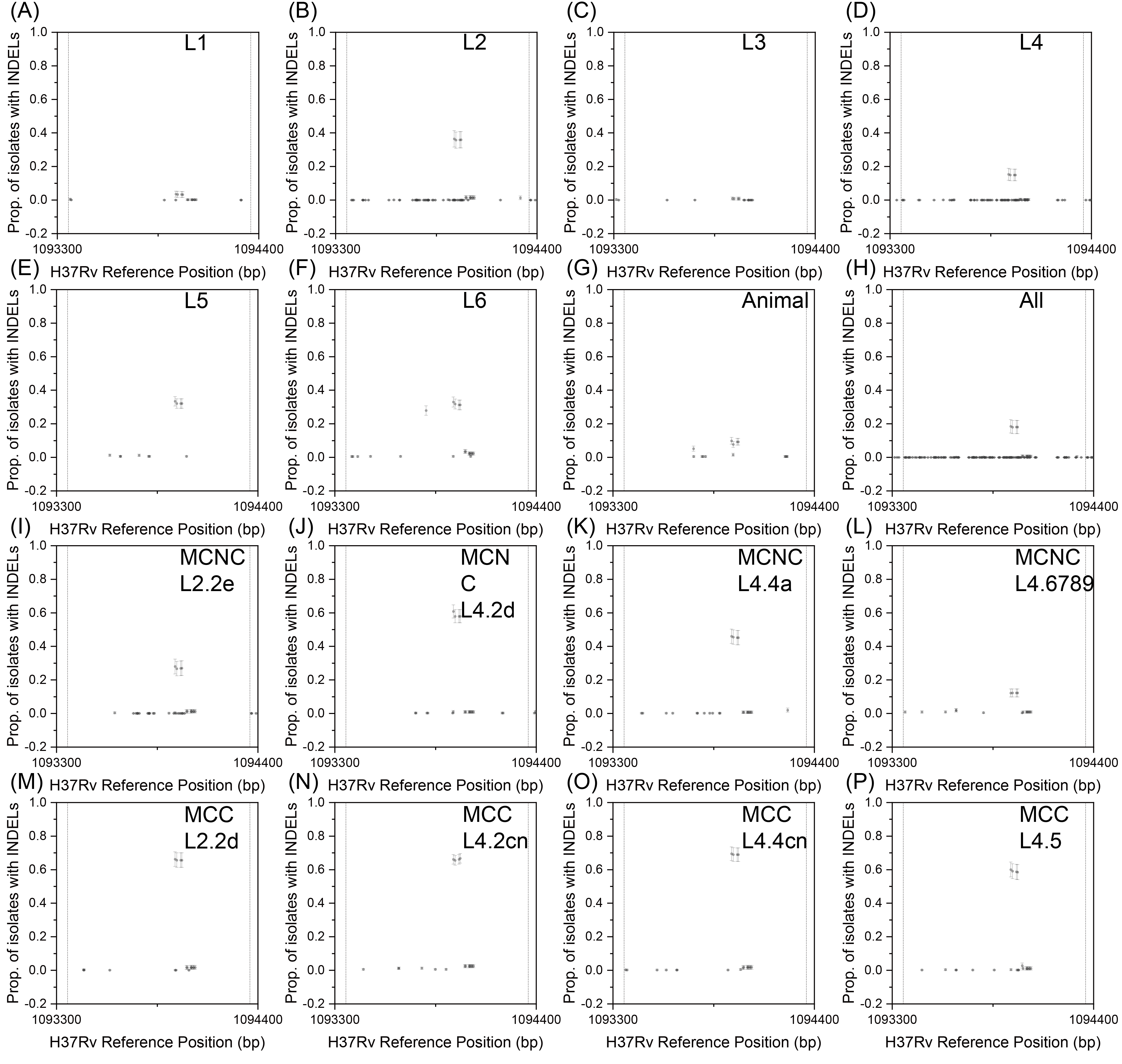


Figure S11. The mean proportion of strains having INDELs at each base site in the gene, *PE_PGRS17*, through random sampling process. (A−F) Randomly sampled strains come from lineages L1−6. (G) Strains from animal lineages. (H) Strains from all 23,873 MTBC strains. (I−L) Strains from four MCNCs (the MTBC clades predominantly found in China). (M−P) Strains from four MCCs (the MTBC clades predominantly found in China). The range of *PE_PGRS17* represents between the dotted lines.


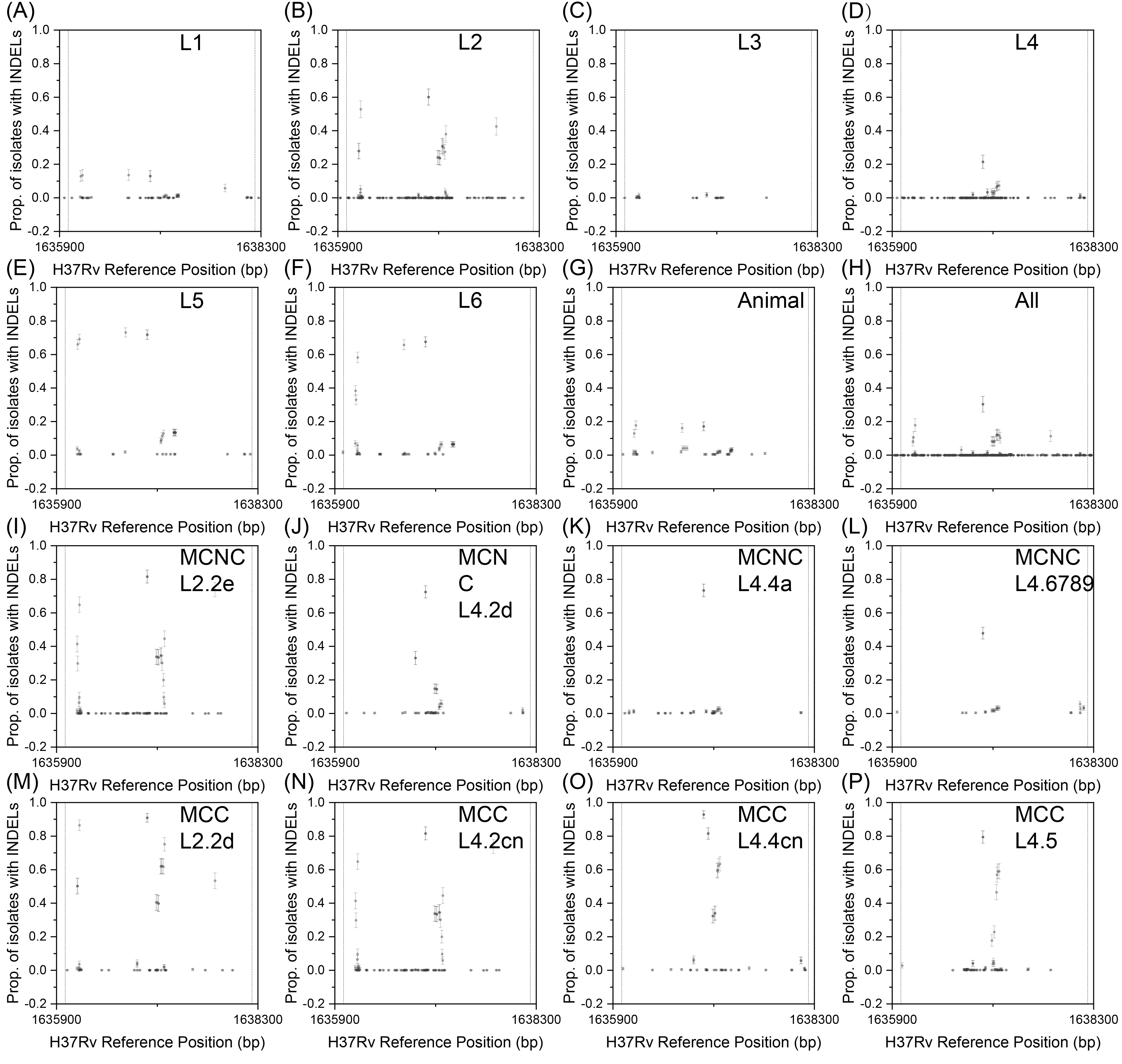


Figure S12. The mean proportion of strains having INDELs at each base site in the gene, *PE_PGRS28*, through random sampling process. (A−F) Randomly sampled strains come from lineages L1−6. (G) Strains from animal lineages. (H) Strains from all 23,873 MTBC strains. (I−L) Strains from four MCNCs (the MTBC clades predominantly found in non-China regions). (M−P) Strains from four MCCs (the MTBC clades predominantly found in China). The range of *PE_PGRS28* represents between the dotted lines.

**Table S1.** Genes with high KL and MKL across epidemic MTBC clades in China.

**Table S2.** Genes with high KL and MKL across China’s epidemic MTBC Clades and their references [1−13].

**Table S3.** PCR amplification system for obtaining the sequence of *Rv0801*.

**Table S4.** The enzyme-digest system of pALACE.

**Table S5.** The ligation system of *Rv0801* and pALACE.

**Data S1. (DataS1.xlsx)**

Collected genotyping results of MTBC. In this table, * represents the province of China. The sources for the data in the table can be found in the references [14−57].

**Data S2. (DataS2.xlsx)**

(1) L2 and L4 strains with whole-genome sequencing data using for phylogenetic construction. (2) L2 and L4 strains with whole-genome sequencing data using for dating and Bayesian phylodynamic analysis. (3) L2 and L4 isolates with whole-genome sequencing data using for Bayesian Skyline Plot analysis.

**Data S3. (DataS3.xlsx)**

L1-9 isolates with whole-genome sequencing data using for reconstructing ancestral whole-genome sequence.

**Data S4. (DataS4.xlsx)**

(1) MTBC strains with whole-genome sequencing data using in this study. (2) MTBC isolates collected from Southwest China with whole-genome sequencing data using in this study.

**Data S5. (DataS5.xlsx)**

Historical temperature anomaly of North, South and Southwest China. The data for the temperature anomaly of North China, South China, and Southwest China respectively come from studies by Zhang et al. [58], Wen et al. [59], and Zhao et al. [60].

**Data S6. (DataS6.xlsx)**

Historical population size in China. In this table, data marked with * come from China's seven national population censuses and represent actual population data, sourced from the public data of China's National Bureau of Statistics (https://www.stats.gov.cn/sj/pcsj). Earlier population data are derived from verified records in ancient Chinese documents or estimated based on historical records and population growth rates, and the raw data are collected from these studies [61−65].

**Data S7. (DataS7.xlsx)**

Historical epidemic outbreaks in China. In this table, "province" represents the modern Chinese provinces to which the regions documented with epidemic outbreaks in ancient Chinese literature geographically belong. If there are two or more recorded outbreaks in the same province within the same year, they are not counted repeatedly. The data are compiled from the series, *Compilation of Three Thousand Years of Epidemic Disasters in China* [66], which contains 34,056 annual records of epidemic from ancient Chinese historical documents.

**Data S8. (DataS8.xlsx)**

1. B and T cell epitopes of MTBC using in this study. (2) Gene groups using in this study.

***Note:** Data S1-8 are available in Github:

https://github.com/WeiWuOpen/MTBCinChina-SupplementalData.

**Supplemental References**

1. Li, Yuanyuan, Lei Fu, Weiyan Zhang, Xi Chen, Yu Lu. 2021. “The Transcription Factor Rv1453 Regulates the Expression of qor and Confers Resistant to Clofazimine in *Mycobacterium tuberculosis*.” *Infection and Drug Resistance* 14: 3937−3948. <https://doi.org/10.2147/IDR.S324043>

2. Zheng, Qianqian, Yunlong Song, Wei Zhang, Neil Shaw, Weihong Zhou, Zihe Rao. 2015. “Structural views of quinone oxidoreductase from *Mycobacterium tuberculosis* reveal large conformational changes induced by the co‐factor.” *The FEBS Journal* 282: 2697−2707. <https://doi.org/10.1111/febs.13312>

3. Velmurugan, Kamalakannan, Bing Chen, Jessica L Miller, Sharon Azogue, Serdar Gurses, Tsungda Hsu, Michael Glickman, William R Jacobs Jr, Steven A Porcelli, Volker Briken. 2007. “*Mycobacterium tuberculosis* nuoG is a virulence gene that inhibits apoptosis of infected host cells.” *PLOS Pathogens* 3: e110. <https://doi.org/10.1371/journal.ppat.0030110>

4. Montoya-Rosales, Alejandra, Roberta Provvedi, Flor Torres-Juarez, Jose A Enciso-Moreno, Rogelio Hernandez-Pando, Riccardo Manganelli, Bruno Rivas-Santiago. 2017. “lysX gene is differentially expressed among *Mycobacterium tuberculosis* strains with different levels of virulence.” *Tuberculosis* 106: 106−117. <https://doi.org/10.1016/j.tube.2017.07.005>

5. Billig, Sandra, Marie Schneefeld, Claudia Huber, Guntram A. Grassl, Wolfgang Eisenreich, Franz-Christoph Bange. 2017. “Lactate oxidation facilitates growth of *Mycobacterium tuberculosis* in human macrophages.” *Scientific Reports* 7: 6484. <https://doi.org/10.1038/s41598-017-05916-7>

6. Stanley, Sydney, Xin Wang, Qingyun Liu, Young Yon Kwon, Abigail M. Frey, Nathan D. Hicks, Andrew J. Vickers, Sheng Hui, Sarah M. Fortune. 2024. “Ongoing evolution of the *Mycobacterium tuberculosis* lactate dehydrogenase reveals the pleiotropic effects of bacterial adaption to host pressure.” *PLOS Pathogens* 20: e1012050. <https://doi.org/10.1371/journal.ppat.1012050>

7. Székely, Rita, Monica Rengifo-Gonzalez, Vinayak Singh, Olga Riabova, Andrej Benjak, Jérémie Piton, Mena Cimino, Etienne Kornobis, Valerie Mizrahi, Kai Johnsson. 2020. “6, 11-Dioxobenzo [f] pyrido [1, 2-a] indoles Kill *Mycobacterium tuberculosis* by Targeting Iron–Sulfur Protein Rv0338c (IspQ), A Putative Redox Sensor.” *ACS Infectious Diseases* 6: 3015−3025. <https://doi.org/10.1021/acsinfecdis.0c00531>

8. Manganelli, Riccardo. 2014. “Sigma Factors: Key Molecules in *Mycobacterium tuberculosis* Physiology and Virulence.” *Microbiology Spectrum* 2: MGM2-0007−2013. <https://doi.org/10.1128/microbiolspec.MGM2-0007-2013>

9. Puri, Rupangi Verma, P Vineel Reddy, Anil K Tyagi. 2013. “Secreted acid phosphatase (SapM) of *Mycobacterium tuberculosis* is indispensable for arresting phagosomal maturation and growth of the pathogen in guinea pig tissues.” *PLOS ONE* 8: e70514. <https://doi.org/10.1371/journal.pone.0070514>

10. Lagutkin, Denis, Anna Panova, Anatoly Vinokurov, Alexandra Gracheva, Anastasia Samoilova, Irina Vasilyeva. 2022. “Genome-Wide Study of Drug Resistant *Mycobacterium tuberculosis* and Its Intra-Host Evolution during Treatment.” *Microorganisms* 10: 1440. <https://doi.org/10.3390/microorganisms10071440>

11. Cao, Tingming, Lingna Lyu, Hongyan Jia, Jinghui Wang, Fengjiao Du, Liping Pan, Zihui Li, et al. 2019. “A Two-Way Proteome Microarray Strategy to Identify Novel *Mycobacterium tuberculosis*-Human Interactors.” *Frontiers in Cellular and Infection Microbiology* 9: 65. <https://doi.org/10.3389/fcimb.2019.00065>

12. Anand, Pragya, Yusuf Akhter. 2022. “A review on enzyme complexes of electron transport chain from *Mycobacterium tuberculosis* as promising drug targets.” *International Journal of Biological Macromolecules* 212: 474−494. <https://doi.org/10.1016/j.ijbiomac.2022.05.124>

13. Sutcliffe, Iain C., Dean J. Harrington. 2004. “Lipoproteins of *Mycobacterium tuberculosis*: an abundant and functionally diverse class of cell envelope components.” *FEMS Microbiology Reviews* 28: 645−659. <https://doi.org/10.1016/j.femsre.2004.06.002>

14. Liu, Qingyun, Aijing Ma, Lanhai Wei, Yu Pang, Beibei Wu, Tao Luo, Yang Zhou, Hong-Xiang Zheng, Qi Jiang, Mingyu Gan. 2018. “China's tuberculosis epidemic stems from historical expansion of four strains of *Mycobacterium tuberculosis*.” *Nature Ecology & Evolution* 2: 1982−1992. <https://doi.org/10.1038/s41559-018-0680-6>

15. Cooksey, Robert C., Said H. Abbadi, Charles L. Woodley, David Sikes, Momtaz Wasfy, Jack T. Crawford, Frank Mahoney. 2002. “Characterization of *Mycobacterium tuberculosis* complex isolates from the cerebrospinal fluid of meningitis patients at six fever hospitals in Egypt.” *Journal of Clinical Microbiology* 40: 1651−1655. <https://doi.org/10.1128/jcm.40.5.1651-1655.2002>

16. Phelan, Jody E., Dodge R. Lim, Satoshi Mitarai, Paola Florez de Sessions, Ma Angelica A. Tujan, Lorenzo T. Reyes, Inez Andrea P. Medado, Alma G. Palparan, Ahmad Nazri Mohamed Naim, Song Jie. 2019. “*Mycobacterium tuberculosis* whole genome sequencing provides insights into the Manila strain and drug-resistance mutations in the Philippines.” *Scientific Reports* 9: 9305. <https://doi.org/10.1038/s41598-019-45566-5>

17. Mendis, Charitha, Vasanthi Thevanesam, Athula Kumara, Susiji Wickramasinghe, Dushantha Madegedara, Chandika Gamage, Stephen V. Gordon, Yasuhiko Suzuki, Champa Ratnatunga, Chie Nakajima. 2019. “Insight into genetic diversity of *Mycobacterium tuberculosis* in Kandy, Sri Lanka reveals predominance of the Euro-American lineage.” *International Journal of Infectious Diseases* 87: 84−91. <https://doi.org/10.1016/j.ijid.2019.07.001>

18. Rajapaksa, U. S., T. C. Victor, A. J. Perera, R. M. Warren, S. M. Senevirathne. 2008. “Molecular diversity of *Mycobacterium tuberculosis* isolates from patients with pulmonary tuberculosis in Sri Lanka.” *Transactions of the Royal Society of Tropical Medicine and Hygiene* 102: 997−1002. <https://doi.org/10.1016/j.trstmh.2008.04.025>

19. Varma-Basil, Mandira, Anshika Narang, Soumitesh Chakravorty, Kushal Garima, Shraddha Gupta, Naresh Kumar Sharma, Astha Giri, et al. 2016. “A snapshot of the predominant single nucleotide polymorphism cluster groups of *Mycobacterium tuberculosis* clinical isolates in Delhi, India.” *Tuberculosis (Edinburgh, Scotland)* 100: 72−81. <https://doi.org/10.1016/j.tube.2016.07.007>

20. Sharma, Pragya, Kiran Katoch, Shilpi Chandra, Devendra Singh Chauhan, Vishnu Dutt Sharma, David Couvin, Nalin Rastogi, Vishwa Mohan Katoch. 2017. “Comparative study of genotypes of *Mycobacterium tuberculosis* from a Northern Indian setting with strains reported from other parts of India and neighboring countries.” *Tuberculosis (Edinburgh, Scotland)* 105: 60−72. <https://doi.org/10.1016/j.tube.2017.04.003>

21. Varma-Basil, Mandira, Anshika Narang, Soumitesh Chakravorty, Kushal Garima, Shraddha Gupta, Naresh Kumar Sharma, Astha Giri, et al. 2016. “A snapshot of the predominant single nucleotide polymorphism cluster groups of *Mycobacterium tuberculosis* clinical isolates in Delhi, India.” *Tuberculosis* 100: 72−81. <https://doi.org/https://doi.org/10.1016/j.tube.2016.07.007>

22. Sharma, Pragya, Kiran Katoch, Shilpi Chandra, Devendra Singh Chauhan, Vishnu Dutt Sharma, David Couvin, Nalin Rastogi, Vishwa Mohan Katoch. 2017. “Comparative study of genotypes of *Mycobacterium tuberculosis* from a Northern Indian setting with strains reported from other parts of India and neighboring countries.” *Tuberculosis* 105: 60−72. <https://doi.org/https://doi.org/10.1016/j.tube.2017.04.003>

23. Manson, Abigail L., Thomas Abeel, James E. Galagan, Jagadish Chandrabose Sundaramurthi, Alex Salazar, Thies Gehrmann, Siva Kumar Shanmugam, et al. 2017. “*Mycobacterium tuberculosis* Whole Genome Sequences From Southern India Suggest Novel Resistance Mechanisms and the Need for Region-Specific Diagnostics.” *Clinical Infectious Diseases* 64: 1494−1501. <https://doi.org/10.1093/cid/cix169>

24. Joseph, Biljo V., Smitha Soman, Indulakshmi Radhakrishnan, Véronique Hill, D. Dhanasooraj, R. Ajay Kumar, Nalin Rastogi, Sathish Mundayoor. 2013. “Molecular epidemiology of *Mycobacterium tuberculosis* isolates from Kerala, India using IS6110-RFLP, spoligotyping and MIRU-VNTRs.” *Infection, Genetics and Evolution* 16: 157−164. <https://doi.org/10.1016/j.meegid.2013.01.012>

25. Gutierrez, M. Cristina, Niyaz Ahmed, Eve Willery, Sujatha Narayanan, Seyed E. Hasnain, Devendra S. Chauhan, Vishwa M. Katoch, Véronique Vincent, Camille Locht, Philip Supply. 2006. “Predominance of ancestral lineages of *Mycobacterium tuberculosis* in India.” *Emerging Infectious Diseases* 12: 1367−1374. <https://doi.org/10.3201/eid1209.050017>

26. Blouin, Yann, Yolande Hauck, Charles Soler, Michel Fabre, Rithy Vong, Céline Dehan, Géraldine Cazajous, et al. 2012. “Significance of the identification in the Horn of Africa of an exceptionally deep branching *Mycobacterium tuberculosis* clade.” *PLOS ONE* 7: e52841. <https://doi.org/10.1371/journal.pone.0052841>

27. Ferdinand, Séverine, Christophe Sola, Suzanne Chanteau, Herimanana Ramarokoto, Tiana Rasolonavalona, Voahangy Rasolofo-Razanamparany, Nalin Rastogi. 2005. “A study of spoligotyping-defined *Mycobacterium tuberculosis* clades in relation to the origin of peopling and the demographic history in Madagascar.” *Infection, Genetics and Evolution* 5: 340−348. <https://doi.org/10.1016/j.meegid.2004.10.002>

28. Bainomugisa, Arnold, Ella M. Meumann, Giri Shan Rajahram, Rick Twee-Hee Ong, Lachlan Coin, Dawn Carmel Paul, Timothy William, Christopher Coulter, Anna P. Ralph. 2021. “Genomic epidemiology of tuberculosis in eastern Malaysia: insights for strengthening public health responses.” *Microbial Genomics* 7: 000573. <https://doi.org/10.1099/mgen.0.000573>

29. Noorizhab Fakhruzzaman, Mohd Nur, Norzuliana Zainal Abidin, Zirwatul Adilah Aziz, Wai Feng Lim, Johari James Richard, Mohd Noordin Noorliza, Mat Hussin Hani, et al. 2019. “Diversified lineages and drug-resistance profiles of clinical isolates of *Mycobacterium tuberculosis* complex in Malaysia.” *International Journal of Mycobacteriology* 8: 320−328. <https://doi.org/10.4103/ijmy.ijmy_144_19>

30. Tan, Joon Liang, Alfred Simbun, Kok-Gan Chan, Yun Fong Ngeow. 2020. “Genome sequence analysis of multidrug-resistant *Mycobacterium tuberculosis* from Malaysia.” *Scientific Data* 7: 135. <https://doi.org/10.1038/s41597-020-0475-x>

31. Gurjav, Ulziijargal, Baasansuren Erkhembayar, Buyankhishig Burneebaatar, Erdenegerel Narmandakh, Oyuntuya Tumenbayar, Grant A. Hill-Cawthorne, Ben J. Marais, Vitali Sintchenko. 2016. “Transmission of multi-drug resistant tuberculosis in Mongolia is driven by Beijing strains of *Mycobacterium tuberculosis* resistant to all first-line drugs.” *Tuberculosis (Edinburgh, Scotland)* 101: 49−53. <https://doi.org/10.1016/j.tube.2016.07.010>

32. Aung, Htin Lin, Thanda Tun, Danesh Moradigaravand, Claudio U. Köser, Wint Wint Nyunt, Si Thu Aung, Thandar Lwin, et al. 2016. “Whole-genome sequencing of multidrug-resistant *Mycobacterium tuberculosis* isolates from Myanmar.” *Journal of Global Antimicrobial Resistance* 6: 113−117. <https://doi.org/10.1016/j.jgar.2016.04.008>

33. Viegas, Sofia O., Adelina Machado, Ramona Groenheit, Solomon Ghebremichael, Alexandra Pennhag, Paula S. Gudo, Zaina Cuna, Paolo Miotto, Véronique Hill, Tatiana Marrufo. 2010. “Research article Molecular diversity of *Mycobacterium tuberculosis* isolates from patients with pulmonary tuberculosis in Mozambique.” *BMC Microbiology* 10: 1−8. <https://doi.org/10.1186/1471-2180-10-195>

34. Stavrum, Ruth, Matsie Mphahlele, Kristi Øvreås, Tshilidzi Muthivhi, P. Bernard Fourie, Karin Weyer, Harleen M. S. Grewal. 2009. “High diversity of *Mycobacterium tuberculosis* genotypes in South Africa and preponderance of mixed infections among ST53 isolates.” *Journal of Clinical Microbiology* 47: 1848−1856. <https://doi.org/10.1128/JCM.02167-08>

35. Perdigão, João, Hugo Silva, Diana Machado, Rita Macedo, Fernando Maltez, Carla Silva, Luisa Jordao, Isabel Couto, Kim Mallard, Francesc Coll. 2014. “Unraveling *Mycobacterium tuberculosis* genomic diversity and evolution in Lisbon, Portugal, a highly drug resistant setting.” *BMC Genomics* 15: Article 991. <https://doi.org/10.1186/1471-2164-15-991>

36. Sekizuka, Tsuyoshi, Akifumi Yamashita, Yoshiro Murase, Tomotada Iwamoto, Satoshi Mitarai, Seiya Kato, Makoto Kuroda. 2015. “TGS-TB: Total Genotyping Solution for *Mycobacterium tuberculosis* Using Short-Read Whole-Genome Sequencing.” *PLOS ONE* 10: e0142951. <https://doi.org/10.1371/journal.pone.0142951>

37. Sharaf Eldin, Ghada S., Imad Fadl-Elmula, Mohammed S. Ali, Ahmed B. Ali, Abdel Latif G. A. Salih, Kim Mallard, Christian Bottomley, Ruth McNerney. 2011. “Tuberculosis in Sudan: a study of *Mycobacterium tuberculosis* strain genotype and susceptibility to anti-tuberculosis drugs.” *BMC Infectious Diseases* 11: 219. <https://doi.org/10.1186/1471-2334-11-219>

38. Nonghanphithak, Ditthawat, Angkana Chaiprasert, Saijai Smithtikarn, Phalin Kamolwat, Petchawan Pungrassami, Virasakdi Chongsuvivatwong, Surakameth Mahasirimongkol, Wipa Reechaipichitkul, Chaniya Leepiyasakulchai, Jody E. Phelan. 2021. “Clusters of drug-resistant *Mycobacterium tuberculosis* detected by whole-genome sequence analysis of nationwide sample, Thailand, 2014–2017.” *Emerging Infectious Diseases* 27: 813−822. <https://doi.org/10.3201/eid2703.204364>

39. Miyahara, Reiko, Nat Smittipat, Tada Juthayothin, Hideki Yanai, Areeya Disratthakit, Worarat Imsanguan, Daranee Intralawan, et al. 2020. “Risk factors associated with large clusters of tuberculosis patients determined by whole-genome sequencing in a high-tuberculosis-burden country.” *Tuberculosis (Edinburgh, Scotland)* 125: 101991. <https://doi.org/10.1016/j.tube.2020.101991>

40. Kibiki, Gibson S., Bert Mulder, Wil M. V. Dolmans, Jessica L. de Beer, Martin Boeree, Noel Sam, Dick van Soolingen, Christophe Sola, Adri G. M. van der Zanden. 2007. “*M. tuberculosis* genotypic diversity and drug susceptibility pattern in HIV-infected and non-HIV-infected patients in northern Tanzania.” *BMC Microbiology* 7: 51. <https://doi.org/10.1186/1471-2180-7-51>

41. Eldholm, Vegard, Mecky Matee, Sayoki G. M. Mfinanga, Manfred Heun, Ulf R. Dahle. 2006. “A first insight into the genetic diversity of *Mycobacterium tuberculosis* in Dar es Salaam, Tanzania, assessed by spoligotyping.” *BMC Microbiology* 6: 76. <https://doi.org/10.1186/1471-2180-6-76>

42. Chaidir, Lidya, C. Carolien Ruesen, Bas E. Dutilh, Ahmad R. Ganiem, Anggriani Andryani, Lika Apriani, Martijn A. Huynen, et al. 2019. “Use of whole-genome sequencing to predict *Mycobacterium tuberculosis* drug resistance in Indonesia.” *Journal of Global Antimicrobial Resistance* 16: 170−177. <https://doi.org/10.1016/j.jgar.2018.08.018>

43. Chaidir, Lidya, Sarah Sengstake, Jessica de Beer, Antonius Oktavian, Hana Krismawati, et al. 2016. “Predominance of modern *Mycobacterium tuberculosis* strains and active transmission of Beijing sublineage in Jayapura, Indonesia Papua.” *Infection, Genetics and Evolution* 39: 187−193. <https://doi.org/10.1016/j.meegid.2016.01.019>

44. Lisdawati, Vivi, Nelly Puspandari, Lutfah Rif’ati, Triyani Soekarno, Melatiwati M., Syamsidar K., Lies Ratnasari, Nur Izzatun, Ida Parwati. 2015. “Molecular epidemiology study of *Mycobacterium tuberculosis* and its susceptibility to anti-tuberculosis drugs in Indonesia.” *BMC Infectious Diseases* 15: 366. <https://doi.org/10.1186/s12879-015-1101-y>

45. Mulenga, Chanda, Isdore C. Shamputa, David Mwakazanga, Nathan Kapata, Françoise Portaels, Leen Rigouts. 2010. “Research article Diversity of *Mycobacterium tuberculosis* genotypes circulating in Ndola, Zambia.” *BMC Infectious Diseases* 10: 177.

46. Blouin, Yann, Yolande Hauck, Charles Soler, Michel Fabre, Rithy Vong, Céline Dehan, Géraldine Cazajous, Pierre-Laurent Massoure, Philippe Kraemer, Akinbowale Jenkins. 2012. “Significance of the identification in the Horn of Africa of an exceptionally deep branching *Mycobacterium tuberculosis* clade.” *PLOS ONE* 7: e52841. <https://doi.org/10.1371/journal.pone.0052841>

47. Comas, Iñaki, Elena Hailu, Teklu Kiros, Shiferaw Bekele, Wondale Mekonnen, Balako Gumi, Rea Tschopp, et al. 2015. “Population Genomics of *Mycobacterium tuberculosis* in Ethiopia Contradicts the Virgin Soil Hypothesis for Human Tuberculosis in Sub-Saharan Africa.” *Current Biology* 25: 3260−3266. <https://doi.org/10.1016/j.cub.2015.10.061>

48. Walker, Timothy M., Thomas A. Kohl, Shaheed V. Omar, Jessica Hedge, Carlos Del Ojo Elias, Phelim Bradley, Zamin Iqbal, Silke Feuerriegel, Katherine E. Niehaus, Daniel J. Wilson. 2015. “Whole-genome sequencing for prediction of *Mycobacterium tuberculosis* drug susceptibility and resistance: a retrospective cohort study.” *The Lancet Infectious Diseases* 15: 1193−1202. <https://doi.org/10.1016/S1473-3099(15)00062-6>

49. Bryant, Josephine M., Anita C. Schürch, Henk van Deutekom, Simon R. Harris, Jessica L. de Beer, Victor de Jager, Kristin Kremer, et al. 2013. “Inferring patient to patient transmission of *Mycobacterium tuberculosis* from whole genome sequencing data.” *BMC Infectious Diseases* 13: 110. <https://doi.org/10.1186/1471-2334-13-110>

50. Casali, Nicola, Vladyslav Nikolayevskyy, Yanina Balabanova, Simon R. Harris, Olga Ignatyeva, Irina Kontsevaya, Jukka Corander, et al. 2014. “Evolution and transmission of drug-resistant tuberculosis in a Russian population.” *Nature Genetics* 46: 279−286. <https://doi.org/10.1038/ng.2878>

51. Stucki, David, Marie Ballif, Thomas Bodmer, Mireia Coscolla, Anne-Marie Maurer, Sara Droz, Christa Butz, et al. 2015. “Tracking a tuberculosis outbreak over 21 years: strain-specific single-nucleotide polymorphism typing combined with targeted whole-genome sequencing.” *The Journal of Infectious Diseases* 211: 1306−1316. <https://doi.org/10.1093/infdis/jiu601>

52. Clark, Taane G., Kim Mallard, Francesc Coll, Mark Preston, Samuel Assefa, David Harris, Sam Ogwang, et al. 2013. “Elucidating emergence and transmission of multidrug-resistant tuberculosis in treatment experienced patients by whole genome sequencing.” *PLOS ONE* 8: e83012. <https://doi.org/10.1371/journal.pone.0083012>

53. Shea, Joseph, Tanya A. Halse, Pascal Lapierre, Matthew Shudt, Donna Kohlerschmidt, Patrick Van Roey, Ronald Limberger, Jill Taylor, Vincent Escuyer, Kimberlee A. Musser. 2017. “Comprehensive Whole-Genome Sequencing and Reporting of Drug Resistance Profiles on Clinical Cases of *Mycobacterium tuberculosis* in New York State.” *Journal of Clinical Microbiology* 55: 1871−1882. <https://doi.org/10.1128/JCM.00298-17>

54. Lin, Dingwen, Zhezhe Cui, Virasakdi Chongsuvivatwong, Prasit Palittapongarnpim, Angkana Chaiprasert, Wuthiwat Ruangchai, Jing Ou, Liwen Huang. 2020. “The geno-spatio analysis of *Mycobacterium tuberculosis* complex in hot and cold spots of Guangxi, China.” *BMC Infectious Diseases* 20: 462. <https://doi.org/10.1186/s12879-020-05189-y>

55. Liu, Mei, Peng Xu, Xingwei Liao, Qing Li, Wei Chen, Qian Gao, Nana Li, Tao Luo, Ling Chen. 2021. “Molecular epidemiology and drug-resistance of tuberculosis in Luodian revealed by whole genome sequencing.” *Infection, Genetics and Evolution* 93: 104979. <https://doi.org/10.1016/j.meegid.2021.104979>

56. Jiang, Qi, Hai-can Liu, Qing-yun Liu, Jody E. Phelan, Li Shi, Min Gao, Xiu-qin Zhao, Jian Wang, Judith R. Glynn, Chong-guang Yang. 2021. “Evolutionary trajectories and transmission dynamics of multidrug-resistant *Mycobacterium tuberculosis* in Tibet, China.” *medRxiv* 2021.02.19.21252065. <https://doi.org/10.1101/2021.02.19.21252065>

57. Dou, Horng-Yunn, Yih-Yuan Chen, Shu-Chen Kou, Ih-Jen Su. 2015. “Prevalence of *Mycobacterium tuberculosis* strain genotypes in Taiwan reveals a close link to ethnic and population migration.” *Journal of the Formosan Medical Association* 114: 484−488. <https://doi.org/10.1016/j.jfma.2014.07.006>

58. Zhang, Can, Cheng Zhao, Aifeng Zhou, Haixia Zhang, Weiguo Liu, Xiaoping Feng, Xiaoshuang Sun, Tianlong Yan, Chengcheng Leng, Ji Shen. 2021. “Quantification of temperature and precipitation changes in northern China during the ‘5000-year’ Chinese History.” *Quaternary Science Reviews* 255: 106819. <https://doi.org/10.1016/j.quascirev.2021.106819>

59. Wen, Fei, Aihua You, Jibin Xue. 2021. “Integrated reconstruction and analysis of temperature and precipitation changes in Southeastern China during the last 2000 years [in Chinese].” *Tropical Geography* 41: 778−789.

60. Zhao, Cheng, Eelco J. Rohling, Zhengyu Liu, Xiaoqiang Yang, Enlou Zhang, Jun Cheng, Zhonghui Liu, Zhisheng An, Xiangdong Yang, Xiaoping Feng. 2021. “Possible obliquity-forced warmth in southern Asia during the last glacial stage.” *Science Bulletin* 66: 1136−1145. <https://doi.org/10.1016/j.scib.2020.11.016>

61. He, Bingli. 1989. “Studies on population of China 1368−1953 [in Chinese].” Shanghai Classics Publishing House.

62. Ge, Jianxiong, Shuji Cao. 1995. “New estimates of the total population during the Ming Dynasty [in Chinese].” *Journal of Chinese Historical Studies* 33−44.

63. Zhu, Yiming. 2012. “An analysis and estimation on population size and its growth rate in early and middle Qing Dynasty [in Chinese].” *Journal of Central South University (Social Science)* 18: 44−52.

64. Guo, Zhiyong. 2012. “Quantitative analysis on the population in Chinese History [in Chinese].” *Journal of North China Institute of Water Conservancy and Hydroelectric Power (Social Science)* 28: 36−39.

65. Wang, Ruiping. 2001. “Exploration of the mystery of population in Ming Dynasty [in Chinese].” *Journal of Zhengzhou University* 34: 62−66.

66. Gong, Shengsheng. 2019. “Compilation of Three Thousand Years of Epidemic Disasters in China [in Chinese].” Shandong Qilu Press Co., Ltd.
